# Patterns of Variation in the Usage of Fatty Acid Chains among Classes of Ester and Ether Neutral Lipids and Phospholipids in the Queensland Fruit Fly

**DOI:** 10.3390/insects14110873

**Published:** 2023-11-14

**Authors:** Shirleen S. Prasad, Matthew C. Taylor, Valentina Colombo, Heng Lin Yeap, Gunjan Pandey, Siu Fai Lee, Phillip W. Taylor, John G. Oakeshott

**Affiliations:** 1 Environment, Commonwealth Scientific and Industrial Research Organisation, Black Mountain, Acton, ACT 2601, Australia; shirleen.prasad@csiro.au (S.S.P.); m.taylor@csiro.au (M.C.T.); vn.colombo@gmail.com (V.C.); henglin.yeap@csiro.au (H.L.Y.); siu.f.lee@gmail.com (S.F.L.); johnoakeshott3@gmail.com (J.G.O.); 2 Applied BioSciences, Macquarie University, North Ryde, NSW 2109, Australia; phil.taylor@mq.edu.au; 3 Australian Research Council Centre for Fruit Fly Biosecurity Innovation, Macquarie University, North Ryde, NSW 2109, Australia; 4 Health and Biosecurity, Commonwealth Scientific and Industrial Research Organisation, Parkville, VIC 3052, Australia; 5 Bio21 Molecular Science and Biotechnology Institute, University of Melbourne, Parkville, VIC 3052, Australia

**Keywords:** *Bactrocera tryoni*, comparative lipidomics, neutral lipids, phospholipids, ether lipids, comparative genomics

## Abstract

**Simple Summary:**

This paper reports the first lipidomic analysis of a tephritid fruit fly. It shows broadly similar lipid profiles to those reported for another dipteran, *Drosophila melanogaster*, but provides greater specification of the individual hydrocarbon chains on particular lipids than has been previously reported in insects. A level of complexity in the configuration of the hydrocarbon chains not previously described in insects is revealed. Genomic analysis reveals a diversity of genes encoding lipid biosynthesis and remodelling enzymes comparable to that seen in mammals, which could account for the complexity of chain configurations observed.

**Abstract:**

Modern lipidomics has the power and sensitivity to elucidate the role of insects’ lipidomes in their adaptations to the environment at a mechanistic molecular level. However, few lipidomic studies have yet been conducted on insects beyond model species such as *Drosophila melanogaster*. Here, we present the lipidome of adult males of another higher dipteran frugivore, *Bactrocera tryoni*. We describe 421 lipids across 15 classes of ester neutral lipids and phospholipids and ether neutral lipids and phospholipids. Most of the lipids are specified in terms of the carbon and double bond contents of each constituent hydrocarbon chain, and more ether lipids are specified to this degree than in any previous insect lipidomic analyses. Class-specific profiles of chain length and (un)saturation are broadly similar to those reported in *D. melanogaster*, although we found fewer medium-length chains in ether lipids. The high level of chain specification in our dataset also revealed widespread non-random combinations of different chain types in several ester lipid classes, including deficits of combinations involving chains of the same carbon and double bond contents among four phospholipid classes and excesses of combinations of dissimilar chains in several classes. Large differences were also found in the length and double bond profiles of the acyl vs. alkyl or alkenyl chains of the ether lipids. Work on other organisms suggests some of the differences observed will be functionally consequential and mediated, at least in part, by differences in substrate specificity among enzymes in lipid synthesis and remodelling pathways. Interrogation of the *B. tryoni* genome showed it has comparable levels of diversity overall in these enzymes but with some gene gain/loss differences and considerable sequence divergence from *D. melanogaster*.

## 1. Introduction

Recent lipidomic analyses of model mammal, yeast and plant species have revealed highly nuanced usage of different acyl/alkyl chains in various classes of lipids [1,2,3,4,5,6,7] and evidence is accumulating that much of the variation could be integral to fundamental aspects of physiology such as the storage and mobilisation of energy reserves, the functioning of membranes and the performance of signalling molecules [3,8,9,10,11,12,13,14]. Lipidomic studies of insects have yet to interrogate their lipid contents in the same level of detail. However, as poikilothermic and metamorphic animals, the demands on such processes in insects might be expected to vary, not just from the mammal and plant models but also within and between species in response to environmental and developmental differences [15,16,17,18,19,20].

Historically, much of the work associating insect lipids with phenotypic and fitness traits was based on total lipid contents or major neutral lipid, phospholipid or fatty acid fractions using a variety of colorimetric, chromatography and mass spectrometry methods. The traits implicated by these analyses have included developmental rates, longevity, quiescence and diapause, flight and long-distance migration, and tolerances to climatic and dietary stresses [21,22,23,24,25,26,27,28]. In some cases, lipid differences between closely related species were found to be associated with differences in their ecology [29,30,31,32] and even pest status [33]. In other cases, inherited differences between strains of a given species were found [34,35,36,37,38]. In still other cases, large environmental effects, often dietary [39] or climatic [40,41,42], were observed.

Recent developments in lipidomics have allowed more detailed dissection of differences in insect lipid profiles due to the various physiological, environmental or genetic factors. Much of the work has focussed on the model dipteran *Drosophila melanogaster* [29,30,31,40,41,42,43,44,45,46,47,48,49,50,51,52,53,54], with some also on other drosophilids [55], mosquitoes, particularly *Aedes* species [56,57,58,59], and the silkworm *Bombyx mori* [60,61]. Differences have been seen in the relative amounts of different lipid classes between tissues or stages/ages [43,47,50,51,52,53,54], or in response to climatic stresses [31,40,41,42,46,47,49,53,54,62], bacterial or viral infections [56,57] or dietary changes [43,49,50,51,58]. Presumptively heritable variation has also been found among strains differing in their tolerances to the various abiotic and biotic stresses, often involving differences in the average chain length or degree of unsaturation of the acyl groups attached to particular classes of neutral lipids or phospholipids, specifically di- and triacyl glycerides and phosphatidylcholines and phosphatidylethanolamines (hereafter DGs, TGs, PCs and PEs, respectively) [43,46,47,50,51,53,54,55,57]. Ether lipids have seldom been analysed in insect lipidomic studies to date, although differences due to diet have been reported [50].

Here, we present the first comprehensive lipidomic analysis of any tephritid fruit fly, the Queensland fruit fly (Qfly), *Bactrocera tryoni*. Like *D. melanogaster*, the Qfly is a highly polyphagous dipteran frugivore. The two species share many host species, although the larvae of *D. melanogaster* feed on rotting fruit, whereas Qfly attacks fresh fruit and, as a consequence, is a major horticultural pest [63,64,65,66]. We focus on Qfly males to avoid issues surrounding lipids that the females deposit in their eggs [48]. However, both newly emerged and sexually mature males are analysed because changes in lipid compositions have been described across equivalent stages of *D. melanogaster* [43].

In this study, we have resolved a total of 421 individual lipids across 15 different classes, including 6 classes of ether lipids, and specified the carbon and double bond contents of individual chains within most of the neutral lipids and phospholipids, and more ether lipids than any previous insect study. This, in turn, has enabled us to examine the frequency distributions of acyl and, in the ether lipids, alkyl and alkenyl chains across most of the lipids in the different classes and to test for non-random combinations of the chains attached to the different headgroups. As in the mammal and plant precedents, our results reveal a highly nuanced usage of different sorts of chains in the different classes. Annotation of the relevant gene–enzyme systems in the Qfly genome shows a comparable capacity to encode these complex patterns, although we also find evidence of some copy number and significant sequence divergence from their *D. melanogaster* homologs. Our findings will facilitate more detailed analyses of lipidomic contributions to important phenotypes, and phenotypic differences, than has previously been possible in insects.

## 2. Materials and Methods

### 2.1. Chemicals

All chemicals used for lipid extraction and analysis were LC-MS-grade compounds from Sigma-Aldrich (Castle Hill, Australia).

### 2.2. Fly Stock and Maintenance

The S06 stock [67] used was collected from infested loquats and mulberries in Sydney, Australia in 2006. Culture conditions were as per the protocol of Popa-Báez et al. [68], as modified by Yeap et al. [69], with the additional modification for recent generations that mini capsicums with lightly scarified surfaces were used as oviposition substrates. Larvae were reared on a mashed gel diet [70] and adults were provided ad libitum white sugar (Woolworths, Yarraville, Australia), yeast hydrolysate (MP Biomedicals, Solon, OH, USA) and water. Adults were kept at a density of ~150 in small cages (17.5 × 17.5 × 17.5 cm^3^) or ~250 in medium-sized cages (32.5 × 32.5 × 32.5 cm^3^) (MegaView Science, Taichung, Taiwan). All life stages were maintained at a temperature of 25 ± 1 °C, relative humidity of 65 ± 5% and a 13:11 h light:dark cycle in which the light hours included an hour of stimulated dusk and dawn using 40 W LED lights (Mecator Voyage, Scoresby, Australia).

### 2.3. Total Lipid Extraction

Lipids were extracted from freshly emerged (i.e., <24 h after eclosion, hereafter Day 1) and sexually mature (i.e., 19–24 days, mainly 19 days, after eclosion, hereafter Day 19) males. Flies were provided water but no food for the first 24 h after eclosion and a normal adult diet after the Day 1 collection. Sixteen replicate males were selected at random for assay from a total of six different cages (at a maximum of three from any one cage) for each day, and the Day 1 and Day 19 flies were drawn from the same six cages. 

Following Zhou et al. [71], males selected for assay were placed individually in 15 mL sterile polypropylene centrifuge tubes (Corning, city, Australia), snap frozen in liquid nitrogen and stored at −80 °C. Subsequently, their wings were removed and they were quickly weighed and placed in 2 mL Eppendorf tubes, which were then covered with perforated parafilm and freeze-dried (Alpha 1-2 LDplus, John Morris Scientific, Chatswood, Australia) at −80 °C for ~72 h. 

Following Guan et al. [52], the freeze-dried flies were homogenised in the Eppendorf tubes using a 5 mm stainless steel bead and a TissueLyser (Qiagen, Clayton, Australia) at 30 Hz for 2 min. The bead was then removed, the homogenates were precipitated by centrifugation at 18,110× *g* for 60 s, and 100 µL of cold methanol: water solution (40:60 *v*/*v*, 4 °C) was added, with vortexing (Ratek Instruments, Knox, Australia) for 10 s. Then, 600 µL of chloroform: methanol solution (1:2 *v*/*v*, 4 °C) was added with vortexing for another 10 s. Samples were then sonicated (Unisonics, Brookvale, Australia) at 40 kHz and 4 °C for 5 min. Following centrifugation at 18,110× *g* for 15 min, the supernatant was pipetted into a new 2 mL Eppendorf tube and 200 µL of chloroform and 300 µL of distilled H_2_O were added. The mixture was vortexed for 10 s and centrifuged at 18,110× *g* for 5 min. The organic phase (~90% of the total volume) was carefully withdrawn with a pipette and transferred to a clean 2 mL standard amber LC-MS vial (Shimadzu Corporation, Kyoto, Japan). Lipids were then re-extracted from the remainder with 400 µL of chloroform and the organic phase (~90% volume) combined with the first extract, which was then completely dried under nitrogen at 30 °C for 10–15 min and stored at −80 °C. All sample manipulations during the extraction procedure were carried out on ice.

### 2.4. Liquid Chromatography–Mass Spectrometry (LC-MS)

The dried extracts were resuspended in butanol:methanol (1:1 *v*/*v*) solution at a ratio of 50 µL per mg of dry tissue. Then, 1 µL of this solution was injected into a Vanquish Horizon ultra-high pressure liquid chromatography (UHPLC) system coupled to an Orbitrap Fusion^TM^ Tribrid^TM^ Mass Spectrometer (2015 version, ThermoFisher, Bremen, Germany) equipped with polarity switching and controlled using Xcalibur software (version 4.2.28.14, ThermoFisher). Conditions for the LC-MS were broadly based on those of Rampler et al. [72]. Chromatographic separation was achieved over 30 min on an Accucore C30 column (150 × 2.1 mm, particle size 2.6 µm, ThermoFisher) held at 40 °C. The mobile phase consisted of a gradient of two solvents: solvent A was acetonitrile:water (3:2 *v*/*v*, 0.1% formic acid, 10 mM ammonium formate, 500 µM phosphoric acid) and solvent B was isopropanol: acetonitrile (9:1 *v*/*v*, 0.1% formic acid, 10 mM ammonium formate). The flow rate was 0.26 µL min^−1^ and elution was achieved with a gradient of solvent B starting at 30% B, with increments to 43% at 2 min, 55% at 2.1 min, 65% at 12 min, 85% at 18 min and, finally, 100% at 20 min, where it was held until 25 min. H-ESI (heated electrospray ionisation) settings were sheath gas, 35 arb units; sweep gas, 5 arb units; auxiliary gas, 1 arb unit; vaporiser temperature, 300 °C. The spray voltage was 3500 V in positive ion mode and 2400 V in negative ion mode and the ion transfer tube temperature was 300 °C. MS spectra were recorded in the scan range 250–1500 *m*/*z* at 120,000 resolution (at 200 *m*/*z*) using the Orbitrap mass analyser with an automated gain control of 4.0 × 10^5^ ions. Data-dependent MS/MS spectra were recorded using the ion trap mass analyser following mass isolation at 0.7 *m*/*z*, with HCD (higher energy collisional dissociation) collision energy stepped between 25, 30 and 35 eV in positive ion mode and held at 30 eV in negative ion mode. In positive ion mode, the PC headgroup daughter ion at 184 *m*/*z* triggered CID (collision-induced dissociation) fragmentation of the parent ion at 32 eV and the neutral loss of fatty acids triggered MS^3^ of TG species at CID 35 eV. 

LipidSearch software (ThermoFisher, version 4.1.3, Waltham, MA, USA) was used to annotate the mass spectra. The product ion search feature was used to identify lipid species using the Orbitrap database with a precursor mass tolerance of 5.0 ppm and product ion tolerance of 0.5 Da. Both positive and negative ionisation data were used to create the compound lists, which were then manually verified. Quantitative analysis was then carried out on all those compounds using online and in-house spectral libraries and annotation tools in the Compound Discoverer software (ThermoFisher, version 3.1, Waltham, USA). These data were then converted to concentrations (ng/µL) with reference to the results for a range of concentrations of several standards that had been included in every run (Table S1).

### 2.5. Statistical Analysis

Statistical analysis was conducted in R version 4.1.2 [73] using the packages tidyverse [74] and combinat [75]. The relative abundances of individual lipids were expressed as percentages of the total concentrations of all the lipids identified in the respective samples.

In order to test for non-random associations among different acyl chains in each of the nine ester lipid classes that we analysed, we first derived expected frequencies for each combination of chain types in each relevant class under the assumption of random association. Broadly following an approach used by Li et al. [76], we achieved this by modelling a multinomial distribution based on the average frequency of each chain type in the class in question. We then using Student’s *t*-tests to compare the observed and expected frequencies of each combination, with Bonferroni corrections of *p*-values to account for multiple testing.

## 3. Results

### 3.1. Overall Lipidome Compositions

We identified and quantified 370–380 lipids at each of the two ages of adult males sampled, 332 of them in both ages, 43 just at Day 1 and 46 just at Day 19 (Table 1 and Table S2 and Supplementary Dataset S1). Most of the lipids identified were fully specified in terms of the carbon and double bond contents of their individual chains (hereafter chain-specified lipids). Most of those that were not chain-specified were ether lipids.

**Figure 1:**
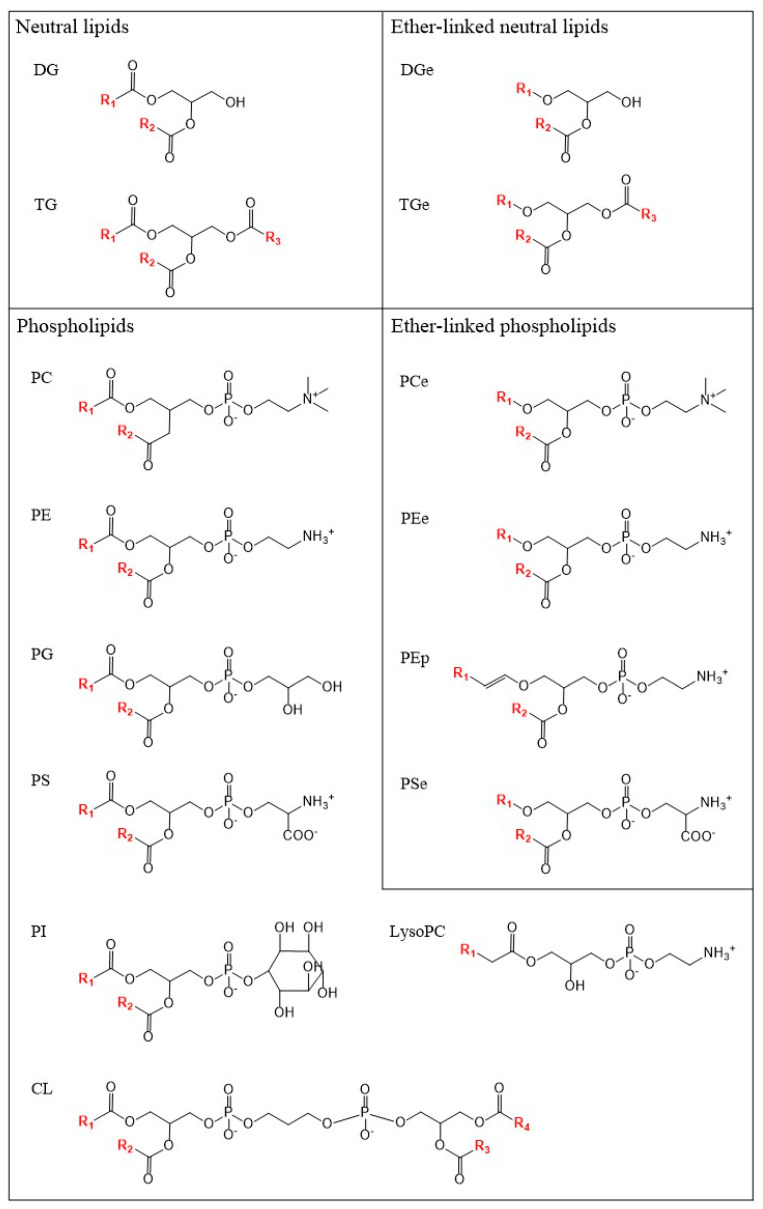
Chemical structures of lipid classes identified in Day 1 and Day 19 males. Hydrocarbon chains are shown in red. Note the alkenyl linkage unique to the PEp.

About half the lipids we identified were TGs, with three classes of phospholipids, namely cardiolipins (CLs), PCs and PEs, accounting for about half of the remainder. The other eleven classes analysed comprised DGs, four additional phospholipid classes, namely phosphatidylglycerols, phosphatidylinositols, phosphatidylserines and lysophosphatidylcholines (PGs, PIs, PSs and LPCs, respectively) and, generally least numerous, six ether lipid classes (plasmanyl TGs, DGs, PCs, PEs and PSs and plasmenyl PEs, hereafter TGes, DGes, PCes, PEes, PSes and PEps, respectively; Figure 1). We also identified several sphingolipids, mainly ceramides, but they were not detected consistently enough for meaningful analysis. The numbers of TGs, CLs and LPCs were at the high end of the range reported in *D. melanogaster* adults, whereas numbers for the other classes were closer to the low end of the *D. melanogaster* range [43,51,52,53,54] (Table S3). Little definitive data on the ether lipids were available in the *D. melanogaster* studies, but fewer TGes were detected in our study than in the one *D. melanogaster* study which could be compared. Only about two-thirds of the Qfly ether lipids were chain-specified, but this was many more than in the *D. melanogaster* precedents.

The TGs, PEs and, to a lesser extent, PCs were the most abundant lipid classes in the Qfly data (Table 1 and Tables S4–S6), which concurs with the findings for *D. melanogaster* [43,51,52,53,54] (Table S7). Our estimates of 3.2–4.2 for the ratio of PE to PC abundances, which is an important determinant of membrane lipid-packing density, and hence membrane fluidity and permeability [10,77,78,79], were also in good agreement with the consensus of values reported from the *D. melanogaster* studies [43,52,53,54] (Table S7). The major differences between the two stages were the lower TG and higher PE values in the Day 19 than Day 1 flies.

Too few abundance data on ether lipids in *D. melanogaster* were available for a meaningful comparison, but our findings that, overall, they comprised around 5% of total lipid amounts are at the low end of the 5–20% generally reported for mammals and the nematode *Caenorhabditis elegans* [3,4,80,81,82]. There was also considerable heterogeneity across Qfly ether lipid classes in their abundances relative to their ester equivalents. For example, the abundance of Qfly DGe was broadly comparable to that of DG, whereas its PCe was manifold less abundant than its PC. Similar class-dependent differences have been reported in other organisms [3,4,80,81,82,83,84,85]. 

Overall, there was a strong positive correlation between the abundances and diversities of the different classes in Qflies at both ages (r^2^ = 0. 68 and 0.73 at Days 1 and 19, respectively; Figure S1).

### 3.2. Average Carbon and Double Bond Contents

As expected, the acyl chains of Qfly neutral lipids and phospholipids contained an average of 16–18 carbons (Table 2). However, within that range (hereafter denoted medium chain length), there were some small but significant differences (i.e., non-overlapping standard errors), which were also consistent across days. Of particular note, TGs had slightly shorter chains than DGs, and PCs had slightly shorter chains than PEs, and indeed those of most other phospholipids. The corresponding data for male *D. melanogaster* showed the same small but consistent difference between the PCs and PEs [43,51,53,54] (Table S8).

None of the DGes or PEes detected were chain-specified, but the data for the chain-specified members of three of the other four ether lipid classes showed significant differences between their acyl and alkyl or alkenyl chains (Table 2). Averages for both the acyl and alkyl groups of the TGes were still in the C16–C18 range. However, the three ether phospholipid classes with some chain-specified members all showed large differences between their acyl and alkyl or alkenyl chains, and most of their lengths lay outside the medium range. The acyl and alkyl chains of the one chain-specified PCe were shorter (~C14) and longer (~C20), respectively, but the larger samples of chain-specified PEes and PEps (8 and 7, respectively) did not show that trend (~C20–21 and ~C23 acyl chains and ~C16 and ~C22–23 alkyl chains, respectively). Few comparable data are available for *D. melanogaster,* but in *C. elegans* and mammalian systems, where more such data exist, both the acyl and alkyl/alkenyl chains of most PCes, PEes and PEps were found to be medium in length [81,82,83,85,86,87,88]. 

The average number of double bonds in the acyl chains of most Qfly neutral lipid and phospholipid classes lay between 1.0 and 1.5. Relatively low values were obtained for the TGs (0.8 and 0.6 on Days 1 and 19, respectively) and, to a lesser extent, PCs (1.0 and 0.9, respectively), and relatively high values in CLs (2.1 and 1.7, respectively) (Table 2). Generally, similar numbers of double bonds have been reported among most of the other neutral lipid and phospholipid classes in *D. melanogaster* [43,51,53,54,89], the (minor) exceptions to this being some lower values for TGs (0.5) and PGs (0.7–0.9) in a few of the latter studies [43,51,53,54] (Table S8).

Double bond numbers in both the acyl and alkyl chains of the chain-specified Qfly TGes (~0.4–1.0) were comparable to those in the acyl chains of TGs (~0.6–0.8) but, as with the carbon content data, there were large differences in those numbers in the acyl vs. alkyl or alkenyl chains of the chain-specified PCe, PEes and PEps (Table 2). Unlike the situation with the carbon content data, however, the direction of differences in the double bond numbers was the same in all three classes. The numbers in their acyl chains were similar or somewhat higher (~1.0–1.4 in the PCe and PEps and ~1.8–2.3 in PEes) than those in the corresponding ester phospholipids, whereas double bonds were rare or absent among their alkyl or alkenyl chains (~0.1–0.2 in PEes and none in the PCe and PEps). Lower double bond numbers in the alkyl/alkenyl than acyl chains of ether phospholipids have also generally been reported in the *C. elegans* and mammalian studies [81,82,83,85,86,87,88].

Although generally not significant in individual phospholipid classes, there was a consistent trend across all the classes of phospholipids, but not the other lipid classes, for there to be slightly fewer double bonds in Day 19 males than Day 1 males (Table 2). This concurs with the small age-related declines in double bond numbers in phospholipid classes reported in adult *D. melanogaster* males [43].

### 3.3. Associations between Carbon and Double Bond Contents

**Figure 2:**
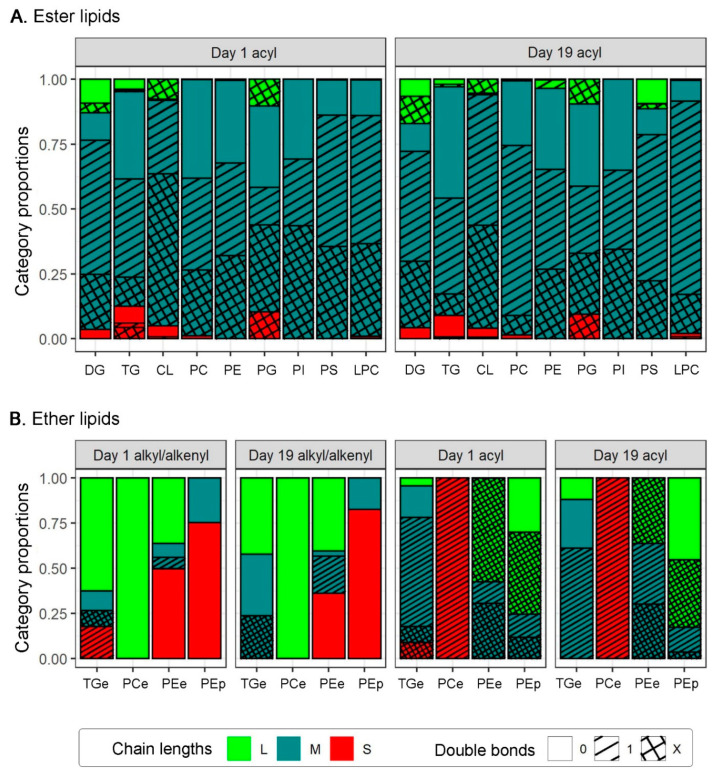
Proportions of chain length and double bond categories in (**A**) the acyl chains of various ester lipid classes, and (**B**) the acyl and alkyl/alkenyl chain length and double bond categories of chain-specified ether lipids, in Day 1 and Day 19 males. Generally, the proportions were based on >8 chain-specified lipid species per class but those for PCe and PEp were only based on 1 and 7, respectively.

To test for any relationship between the average carbon and double bond contents of the chains in each class, we classified each chain in each fully specified lipid both as short, medium or long (S, M, L, for <C16, C16–18 and >C18, respectively) and as saturated, monounsaturated or polyunsaturated (0, 1 and X for zero, one and > one double bond, respectively) (Figure 2). This revealed some clear associations between the two variables in the acyl chains of the neutral lipids and phospholipids. In particular, there was a consistent trend across all nine of these classes for monounsaturation to be disproportionately common in medium-length acyl chains. Short and long chains were generally either saturated or polyunsaturated.

Except in the solitary PCe, whose acyl chain was short and monosaturated, a similar trend was evident in the acyl chains of the chain-specified ether lipids. Once again, monounsaturation was largely confined to the medium acyl chains, with most of the short and long acyl chains again being either saturated or polyunsaturated.

The patterns were more variable across the alkyl/alkenyl chains of the four ether lipid classes analysed. Consistent with the pattern in the acyl chains above, monounsaturation was largely confined to medium alkyl chains in the PEes but it was also seen in some short alkyl chains in the TGes. Polyunsaturated alkyl/alkenyl chains were only seen in the TGes, with most alkyl/alkenyl chains in all four classes being saturated, and with medium alkyl/alkenyl chains of any double bond category generally being rare.

### 3.4. Individually Common Chains

**Figure 3:**
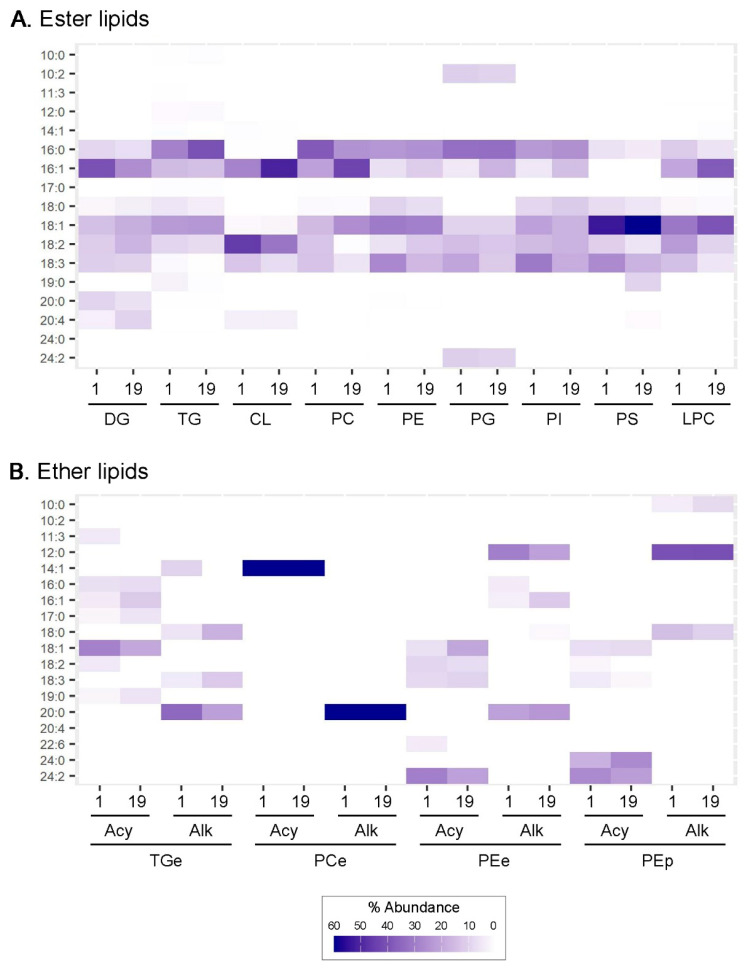
Heatmap showing most common acyl chains in ester lipid classes (**A**) and acyl and alkyl/alkenyl chains in chain-specified members of ether lipid classes (**B**). Common chains in each panel are defined as those occurring at > 5% frequency in at least one class on at least one day. Acy = acyl; Alk = alkyl or alkenyl.

The Qfly dataset contained 18 individually common chains, which we defined as constituting at least 5% of all the chains in at least 1 of the 13 classes for which we had chain-specified members in at least one of the Day 1 or Day 19 datasets (Figure 3A, Tables S9 and S10). These 18 chains ranged in size from 10 to 24 carbons, with all but 3 having even numbers of carbons. Those with odd numbers could reflect dietary intake [30,50] or either the occasional use of propionyl CoA precursors in their synthesis or the addition of (generally) methyl side chains, both of which have been reported, albeit uncommonly, in other insects [90,91,92]. The common chains included five, seven and six that were short, medium and long, respectively, and eight, three and seven that were saturated, mono- and polyunsaturated, respectively. The latter included two heavily polyunsaturated long chains, 20:4 and 22:6.

As found in most other insects (Tables S11 and S12), and indeed most terrestrial organisms that have been characterised [17,18,59,60,91,93,94,95], the six acyl chains most commonly found in all the Qfly neutral lipids and phospholipid classes were the medium chains 16:0, 16:1, 18:0, 18:1, 18:2 and 18:3. The only other acyl chains at 10% or higher frequencies in any of the ester lipid classes were 10:2 and 24:2 in PGs (each at ~10–11% on both days).

The six major medium acyl chains varied in their relative abundances in the different neutral lipid and phospholipid classes, albeit 18:0 was generally among the least common. Each of the other five predominated in at least one class/day. The proportions of 18:2 and 18:3 were generally higher in phospholipids than neutral lipids, which concurs with evidence from various other insects, including in the pre-lipidomic data from two other tephritids, the olive fruit fly *Bactrocera oleae* and Mediterranean fruit fly *Ceratitis capitata* [90,94,95]. However, there was also significant variation in the proportions of certain of the chains between days; for example, the proportions of 16:0, 16:1, 18:2 and 18:3 all differed by more than 10% between days in at least two of the ester lipid classes. Such differences have also been reported previously, both between ages or life stages and between species, including *B. oleae* and *C. capitata* (Tables S11 and S12).

Four of the six medium chains above (mainly 18:1) also contributed the majority of the acyl chains in the chain-specified TGes. However, the alkyl chains of the TGes and both the acyl and alkyl or alkenyl chains of the chain-specified PCe, PEes and PEps were generally short or long, with considerable variation between them as to the specific acyl and alkyl/alkenyl chains that predominated. The most abundant chain was 20:0 among the alkyl chains of the TGes, while 12:0, 14:1, 20:0, 24:0 and 24:2 were each the most abundant acyl or alkyl/alkenyl chain in one or other of the three ether phospholipid classes in question.

Moreover, the frequencies of the specific chains in each of the four ether lipid classes differed radically between their respective acyl and alkyl/alkenyl complements. Despite significant differences in the particular chains involved, the common theme across all four classes was for an essentially complete dichotomy between the acyl and alkyl/alkenyl chain complements of each of them (Figure 3B, Table S10).

We are unaware of any previous reports of the acyl/alkyl chain complements of ether neutral lipids in insects, but in other organisms, the great majority of the alkyl chains of DGes at least are 16:0, 18:0 and 18:1 [4,96]. Two other insect studies, one on adult *D. melanogaster* males [97] and one on larval gut tissue of *B. mori* [98], produced data for six chain-specified ether phospholipids, three PEes and three PEps, respectively. They found only 18:0 in their alkyl/alkenyl chains and 18:1, 18:2 and 18:3 in their acyl chains. More comprehensive studies of these two classes in other organisms generally report high frequencies of 16:0, 18:0 and 18:1, as well as a few 20:0 or 22:0 alkyl and alkenyl moieties, plus high frequencies of 18:2 and 18:3 as well as a few 20:3, 20:4 and 20:5 acyl moieties [81,82]. Thus, the relative paucity of medium acyl or alkyl/alkenyl chains in the Qfly ether phospholipids stands in contrast with the limited data available for other insect species, including *D. melanogaster*. However, our data agree with the limited data available for other insects and the more extensive data for other organisms in finding minimal overlap between the compositions of the acyl vs. alkyl/alkenyl chains of the four ether lipid classes for which we had chain-specified data.

### 3.5. Possible Sampling Biases in the Ether Lipid Data

Given we had found lower proportions of (either acyl or alkyl/alkenyl) medium chains in Qfly ether phospholipids than have been reported for other species, we investigated the possibility that ours was an unrepresentative sample of those lipids in the Qfly lipidome (Table S6). Such a bias could have arisen during either the detection of our ether lipids or, where we could, the resolution of their individual chains.

The scope for detection bias was significant, given that the total number of ether lipids we recovered, 38, may be significantly fewer than the available *D. melanogaster* data suggest may exist (noting that the latter have seldom been chain-specified [51,52]). The scope was greatest for the PCes and PSes, for each of which we only identified a single lipid species, but even for the other four classes, the numbers were only between 6 and 13. 

The scope for bias in chain resolution was significant in the case of the TGes, because the two TGes that were not chain-specified, 32:1e and 34:2e, were also by far the most common (combined frequencies 0.29% at Day 1 and 0.13% at Day 19, cf <0.01% for each of the others on each day they were detected). However, the relatively small aggregate carbon numbers across their three chains (32 and 34) indicated that both included at least one and possibly two short chains. 

None of the six DGes were chain-specified, but five of them each had aggregate carbon numbers of 32–36 that could be explained by individual C16 and C18 chains. However, the sixth, 22:3e, which was also the most common, clearly involved at least one short chain. 

Three of the five unresolved PEes (36:0e, 37:3e, 38:2e, 38:3e, 42:3e) had aggregate carbon numbers implying at least one long chain but the combined frequency of these five PEes was very low anyway (~0.1% on each day). Two of the eight chain-specified PEes each had two medium chains, and the combined frequency of those two was higher (~0.1% at Day 1 and ~0.3% at Day 19). However, the other six each had at least one short or (mainly) long chain, and their combined frequencies were higher still (~1.6% at Day 1 and ~0.9% at Day 19). 

All seven of the PEps detected were resolved; all the chains in four (combined frequencies ~0.6% and 0.4% at Days 1 and 19, respectively) were medium chains, while none of the other three (combined frequencies ~0.8% and ~1.8% at Days 1 and 19, respectively) had any of these chains and each included both a C10 or C12 alkenyl and a C24 acyl chain. The combined frequencies of the first four were lower than those of the last three, although both groups were relatively frequent compared to the other ether phospholipids. 

Thus, relatively low proportions of the six medium chains were recurring features of the TGes, DGes and PEes whether or not they were chain-specified. The situation with the PEps was more complex, with the limited data available suggesting one group only contained such chains and the other only contained short and long combinations. 

The single PCe detected (20:0e_14:1, ~0.01% and ~0.03% at Days 1 and 19, respectively) was chain-specified and had no medium chains, whereas the single PSe (38:5e, ~0.14% and ~0.01% at Days 1 and 19, respectively) was not resolved and both of its chains could have been medium chains. 

In sum, we cannot discount the possibility that the relatively low frequency of medium chains in some of our ether lipid classes was due to biases in their detection. However, for some classes at least, it seems unlikely to be due to the subset that could be chain-specified being a biased sample of those detected.

### 3.6. Non-Random Combinations of Acyl Chains in Ester Lipids

Given the indications in Section 3.3 and Section 3.4 above for very different complements of acyl vs. alkyl/alkenyl chains among Qfly ether lipids, we then applied a statistical test for non-random combinations of carbon and double bond chain types in each multi-chain class of ester lipids. Specifically, we tested whether or not the frequencies of the combinations of chain types in each class were simply products of the individual frequencies of those types in that class. 

About 89% of the comparisons for the nine possible combinations of the three carbon and three double bond categories across the two days showed significant differences between the frequencies observed and those expected under the assumption of random associations (Table S13). Both the carbon and double content differences contributed to the differences; comparisons considering the three carbon content and three double bond categories separately also yielded high frequencies of differences, 79% and 75%, respectively (Tables S14 and S15). The directions of the differences in each analysis were generally consistent across days and high proportions of the differences in every class were significant. 

To screen for the major patterns in the differences, we focussed on 29 large biases in the nine joint carbon and double bond categories (Table 3). For these purposes, we defined large differences as those involving differences greater than 5% between the observed and expected frequency on at least one of the days, and the direction of the effect was the same on both days. These 29 large biases encompassed all eight of the multi-chain ester lipid classes. Fourteen involved excesses ranging in size from 1.5- to 27.4-fold above expectations and 15 involved deficits ranging from 0.7-fold to 0.0-fold below expectations. The strongest pattern involved seven large deficits of two homogeneous combinations among the PCs, PEs, PGs and PIs. Four of these pairings involved two saturated medium-length chains and three involved two polyunsaturated medium-length chains. Offsetting these seven deficits were excesses of various heterogeneous combinations. Most of the latter involved differences in double bond content but, across all 14 large excesses, there were also 5 cases where the pairing involved differences in chain length. Notably also, against the general trend for more homogeneous combinations in the deficits than the excesses, one CL combination in excess involved a homogeneous combination in which all four chains were medium and polyunsaturated.

## 4. Discussion

While the diversities and abundances of the different Qfly lipid classes were generally within the ranges reported for *D. melanogaster*, there were still some classes where our values were at or beyond the extremes of those already quite large ranges. The significance of these differences is unclear, however, given the potentially large contributions of differences between studies in research focus and methodology. 

On the other hand, with a few notable exceptions, the compositional profiles of the different Qfly classes generally fell within the relatively tight ranges of the *D. melanogaster* precedents. This might be expected, given the strong structural constraints associated with their functions in various lipoproteins, lipid droplets and lipid bilayers in membranes [1,9,29,79,99]. Thus, the great majority of the acyl chains in neutral lipids and phospholipids we found in Qfly males were of medium length. Similarly, the six specific chains that were most common in these classes were the major end product of the fatty acid (FA) synthase II system (16:0), its subsequent elongation by FA elongase (18:0) and the desaturation of those two FAs by various desaturases (16:1, 18:1, 18:2 and 18:3). Despite the relatively tight range overall, however, some variations in compositional profiles were seen among the different lipid classes and across the two days, and previous work on other organisms suggests they could have functional significance. 

Most of the differences in the ester neutral lipids and phospholipids involved differences in saturation states. Slightly shorter chains were found in PCs than other phospholipids but proportionately larger differences were seen in average levels of unsaturation, which were relatively low in the TGs and PCs, relatively high in the cardiolipins and, among the phospholipids, consistently higher on Day 1 than Day 19. The relatively low double bond contents of the TG chains were consistent with the findings for *D. melanogaster*, as were the relatively high double bond contents of the cardiolipins and the relatively low carbon, albeit not double bond, contents in the PC chains [43,51,53,54,89].

The higher double bond contents of the phospholipids in the Day 1 compared to Day 19 flies also agreed with differences seen between newly emerged and sexually mature *D. melanogaster* [43] and would be consistent with the younger flies of both species having more flexible membranes while their bodies are still in the process of maturation. Lower levels of saturation in phospholipid chains have been associated with less fluidic membranes in a variety of organisms, including insects, as well as in model membranes [10,40,41,42,78,79]. Notably, this could also affect other functions, such as transmembrane transport [10,40,41,42,78,79].

Many of the physiological effects of acyl chain compositions in PCs and other phospholipids are temperature-dependent and studies in several systems, including *D. melanogaster*, have shown that organisms adjust their membrane phospholipid compositions according to the temperature of their environments (‘homeoviscous adaptation’ [41,47,53,54,77]). Age-related changes in the temperature tolerances have been reported in both *D. melanogaster* [100] and *C. capitata* [101]. We are unaware of any evidence for this in Qflies but inherited differences between strains in various climate stress tolerances have been found in this species [68] and it would now be worthwhile to see whether phospholipid differences contribute to the strain variation. 

The distinct acyl chain profiles we have found in Qfly PCs as compared to several of its other phospholipids may be at least in part a consequence of the deacylation/reacylation processes that underpin phospholipid remodelling [7,102,103,104,105,106]. Elegant work on phospholipid remodelling in a bacterial system [77] has shown that PCs are more often involved in this process than are other phospholipids and the level of (un)saturation introduced varies with temperature. 

Cardiolipins have seldom been studied in insects [50,89,107] but, unlike other phospholipids, they are mainly found in mitochondrial membranes in other organisms [108,109,110,111]. While medium-length chains predominate in the cardiolipins of other species studied, they show higher double bond contents than other phospholipids [109,112]. We also found this to be the case in Qfly males of both ages, with the level again being higher at Day 19 than Day 1. The high level of unsaturation in mammalian cardiolipins has been attributed to structural constraints in the mitochondrial membranes associated with their role in mitochondrial bioenergetics, transport and signalling processes [108].

Some of the lipidomic work on *D. melanogaster* has found differences between early and mid-age adult males in the chain length and double bond contents of TGs [43] and such changes have also been implicated in ageing-related differences in energy storage in other species [113]. Our failure to find such differences between Day 1 and Day 19 Qfly males may reflect the relatively late sexual maturation of Qfly males; *D. melanogaster* males are sexually mature within hours of emergence but Qflies do not begin mating until several days after emergence [114,115]. 

We only recovered a proportion of the ether lipids that studies so far suggest may exist in *D. melanogaster* [51,52,54], whereas our numbers of chain-specified ether lipids, 24 in total, were significantly higher than the precedents in that species or any other insects [97,98]. Historically, ether lipids have been notoriously difficult to extract and separate [81,116] and it may be that our study suffered more from extraction difficulties and less from chain disambiguation difficulties than some of the earlier studies. Notwithstanding these issues, three major trends were evident in the chain composition data for the Qfly ether lipids.

The first major trend was the broader distribution of chain lengths than in the corresponding ester lipid classes. The only chains in the chain-specified ether lipids that did not show this were the acyl chains of ether neutral lipids. This contrasts with the high frequencies of C16 and, in particular, C18 alkyl and alkenyl chains found in both the very limited data from other insects and the larger datasets from mammals and *C. elegans* [59,60,86,94,95]. As noted, it is possible that some detection bias disfavouring ether lipids with medium chains may have been at play in our dataset. However, it may also be relevant that large differences in ether lipid contents due to diet and tissue source have been found in *D. melanogaster* [50].

The second major trend in the chain composition of Qfly ether lipids was the tendency for higher double bond numbers in their acyl than alkyl/alkenyl chains. The trend was particularly strong in their ether phospholipids and, in this respect, our results agree well with studies of other organisms [80,81,82,86]. Interestingly, while not as pronounced as in our data, the polyunsaturated acyl chains in some of the other studies include significant numbers of long chains (e.g., over 20% 20:3–5 in PEps in *C. elegans* [82]). Functional consequences in other organisms include effects on membrane flexibility [86,95,117] and storage of polyunsaturated precursors of various other molecules [86].

The third major trend in the chain composition of Qfly ether lipids, which followed from the first two, was that there was essentially no overlap in the identities of the acyl vs. alkyl or alkenyl chains in individual chain-specified ether lipids. This asymmetry also agrees with the findings from various *C. elegans* and mammalian studies [3,80,83,84,87]. In those studies, the differentiation was heavily dependent on differences in double bond profiles but, consistent with the higher frequencies of short and long alkyl/alkenyl chains in the Qfly ether lipids, differences in chain length profiles also played a role in Qfly.

The functions of insect ether lipids are less well understood than those of their ester equivalents but the available data from other eukaryotes suggest some of the patterns we have found in Qfly ether lipid profiles could impact various physiological functions. These encompass structural effects in membranes that affect their fluidity, which, in turn, has consequences for cell signalling and synapse function in the nervous system, plus some antioxidative effects [3,83,84,118,119]. The precedents from other species also suggest that the properties of the Qfly ether lipids are closely linked to their different positions on the headgroup, with the alkyl/alkenyl groups essentially limited to the *sn1* position [3,11,81,118,120] (see also below).

Our statistical analyses showed that combinations of dissimilar chains were also more frequent than expected simply from their individual frequencies in several classes of ester lipid. Significantly, Li et al. [76], on whose work our statistical analyses were based, also found an excess of dissimilar combinations in the only ester lipid class they analysed in the model plant *Arabidopsis thaliana*; specifically, they reported an excess of TGs with just one elongated acyl chain. And, while not as well established as for the ether lipids (where the acyl vs. alkyl/alkenyl chain distinction facilitates the investigation), there is also a growing body of evidence linking such non-random associations to positional differences on the headgroups of ester lipids in other organisms. For example, Manni et al. [121] found membrane phospholipids in rat brains generally had chains that were unsaturated at *sn1* vs. saturated, monounsaturated or polyunsaturated at *sn2*, where they ranged from 14:0 to 22:6. 

In contrast to the situation in most other classes analysed, Qfly cardiolipins showed an excess of a combination of chains that were all similar in both carbon and double bond contents. In this case, all four of the CL acyl chains were medium in length and polyunsaturated. This concurs with findings in other organisms, including *D. melanogaster*, where there are also largely medium polyunsaturated chains [89,107,109]. Other carbon and double bond contents have been described in the CLs of some other species, but the trend remains for an excess of configurations involving the same acyl chain at each of the four positions on the headgroup [122,123,124]. This apparently widespread trend may reflect the unique role of this particular class of phospholipid in the mitochondrial membranes of many species [89,110,111].

**Figure 4:**
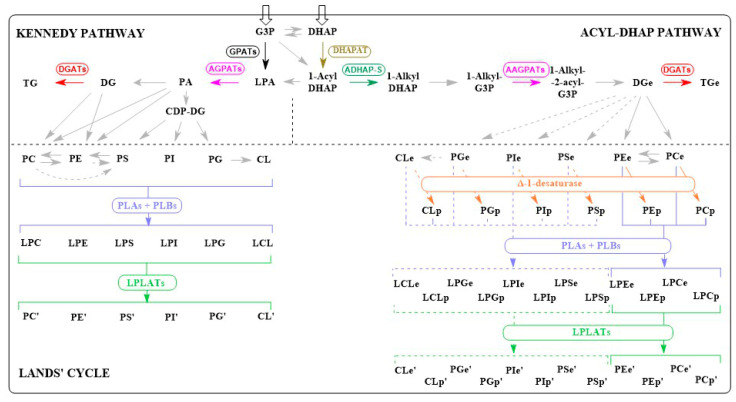
Eight reactions in eukaryote lipid biosynthesis pathways where the substrate specificities of the enzymes will determine the acyl or alkyl/alkenyl chain identities. The Kennedy pathway produces neutral lipids and phospholipids, the ACYL-DHAP pathway produces ether-linked neutral lipids and phospholipids and the Lands’ cycle remodels the acyl/alkyl chain compositions of various lipids. Reactions not directly affecting the acyl/alkyl chain identities are shown in grey and without their cognate enzymes. Reactions with direct effects are shown together with their cognate enzymes and using a different colour for each reaction that involves a different enzyme. Some reactions are indicated with broken lines because direct evidence for the enzymes involved is limited. The GPAT, DHAPAT, PLA and PLB reactions affect chain identities at *sn1*, AGPAT, AAGPAT and PLB those at *sn2*, and DGAT those at *sn3*. Δ1 desaturase converts the ether bond to a vinyl ether linkage. Compound abbreviations not previously explained: CDP-DG—cytidine diphosphate diacylglycerol, DHAP—dihydroxyacetone phosphate, G−3-P—glycerol-3-phosphate, LPA—lysophosphatidic acid, PA—phosphatidic acid. Enzyme abbreviations: DHAPAT—dihydroxyacetone phosphate acyl transferase, ADHAP-S—alkyl dihydroxyacetone phosphate synthase, AAGPAT—alkyl/acyl glycerol-3-phosphate acyltransferase, AGPAT—acyl glycerol-3-phosphate acyltransferase, DGAT—diacylglycerol acyltransferase, PLA—phospholipase A, PLB—phospholipase B. Note that the AGPAT and AAGPAT reactions involve the same enzymes, in the first case, incorporating acyl chains, and in the second case, alkyl chains. For details of the pathways, see [97,103,104,105,106,107,111,115,118,120,125,126,127,128].

Evidence from various organisms has shown that the identities of the chains in each *sn* position on the different lipid headgroups depend in a large part on the substrate specificities of key enzymes in the Kennedy and acyl DHAP lipid biosynthetic pathways for ester and ether phospholipids, respectively, and the Lands’ cycle responsible for remodelling both ester and ether phospholipids [7,105,106,116,120,125,126,127,128]. At least ten enzyme-catalysed steps across these three pathways are directly involved in chain addition or replacement (Figure 4), and in *Homo sapiens,* several of these steps can be catalysed by multiple enzymes with varying substrate specificities and expression profiles [1,6,7,8,11,96,129,130,131,132,133,134,135,136]. Homologues of genes for all ten of the activities occur in both the *D. melanogaster* and Qfly genomes (Table 4), and all the activities for which multiple genes occur in *H. sapiens* also have multiple homologues in the two fruit fly species. Thus, both these species have the genetic potential to produce a variety of chain compositions, and the encoded enzymes may vary in their effects across different classes, depending on differences in either their substrate ranges or expression profiles across tissues or developmental states. 

However, the two fruit fly species also show some presence/absence differences, both from *H. sapiens* and, in some cases, from each other, in the complements of enzymes catalysing particular steps in the pathways (Table 4). Compared to *H. sapiens*, there are fewer glycerolphosphate acyltransferase (GPAT) genes in Qfly but more in *D. melanogaster*, fewer acylglycerolphosphate acyltransferase (AGPAT/AAGPAT) genes in both species, and more diacylglycerol acyltransferase (DGAT) genes in *D. melanogaster*, in the biosynthetic pathways. The two insect species also have more phospholipase A2 (PLA2) but fewer lysophospholipid acyl transferase (LPLAT) genes than *H. sapiens* in the Lands’ cycle. These differences, together with considerable amino acid sequence divergence in all orthologous comparisons, even between the two fruit fly species (>20% in each case; Table 4), also suggest the scope for significant functional divergence between them. 

In conclusion, our analyses have revealed levels of complexity in an insect lipidome comparable to that seen in more intensively studied mammals. Functional data from model systems suggest this complexity could be important for tuning various classes of lipids to suit their diverse functions. Many basic cellular functions will require tight conservation of certain aspects of lipid profiles across species and many aspects of our compositional data indeed closely resemble those reported in *D. melanogaster* and, often also, in more distantly related taxa. On the other hand, some environmentally sensitive functions might be expected to vary between species of metamorphic poikilotherms, in particular. Our comparisons provide some evidence for this, although methodological differences—including, in some cases, potential sampling biases—could also explain much of the variation. However, together with the *D. melanogaster* precedents, our study provides a foundation for future comparative and population lipidomic work, using standardised methodologies and including more life stages and both sexes, to investigate the role which many studies in the pre- and early lipidomic era indicate lipids play in phenotypic variation in ecologically sensitive traits [21,22,23,24,25,26,27,28] (see also Introduction). Because of their pest status, the ecology, nutritional physiology and genomics of several tephritids have been intensively studied [63,64,65,66,68,137,138], so they should be informative models for such analyses.

## Figures and Tables

**Table 1:** Diversity (numbers of lipids detected) and abundance (% by weight of all lipids detected ± SE) of each category and class of lipids in Day 1 and Day 19 males. Category level data are shown in bold.

Class	Diversity		Abundance (±SE)	
	Day 1	Day 19	Day 1	Day 19
**Neutral lipids**	**213**	**196**	**36.5 ± 2.7**	**30.1 ± 2.0**
**DG**	11	16	0.4 ± 0.05	0.4 ± 0.04
**TG**	202	180	36.1 ± 2.7	29.7 ± 2.1
**Phospholipids**	**125**	**148**	**57.6 ± 2.5**	**65.9 ± 1.8**
**CL**	36	34	4.1 ± 0.2	5.1 ± 0.2
**PC**	26	27	9 ± 0.4	8.6 ± 0.2
**PE**	19	27	29.1 ± 1.4	36 ± 1.1
**PG**	9	9	3.5 ± 0.2	4.7 ± 0.1
**PI**	12	15	7.2 ± 0.3	5.4 ± 0.2
**PS**	9	21	3.4 ± 0.2	4.1 ± 0.4
**LPC**	14	15	1.3 ± 0.1	2 ± 0.2
**Ether neutral lipids**	**15**	**15**	**1.6 ± 0.1**	**0.6 ± 0.1**
**DGe**	6	6	1.3 ± 0.1	0.5 ± 0.1
**TGe**	9	9	0.3 ± 0.03	0.2 ± 0.02
**Ether phospholipids**	**22**	**19**	**4.3 ± 0.2**	**3.5 ± 0.2**
**PCe**	1	1	0.01 ± 0	0.03 ± 0
**PEe**	13	11	1.7 ± 0.1	1.2 ± 0.1
**PEp**	7	6	2.4 ± 0.1	2.2 ± 0.1
**PSe**	1	1	0.1 ± 0.01	0.04 ± 0.01

**Table 2:** Average numbers (±SE) of carbons and double bonds per chain in the acyl chains of ester lipids and the various hydrocarbon chains of chain-specified ether lipids in Day 1 and Day 19 males. Lipid categories are shown in bold.

Class	Mean Number of Carbons		Mean Number of Double Bonds	
	Day 1	Day 19	Day 1	Day 19
**Neutral lipids**				
DG	16.8 ± 0.8	17.1 ± 0.6	1.2 ± 0.3	1.5 ± 0.3
TG	16.3 ± 0.3	16.5 ± 0.3	0.8 ± 0.1	0.6 ± 0.1
**Phospholipids**				
CL	17.5 ± 0.2	17.0 ± 0.2	2.1 ± 0.1	1.7 ± 0.1
PC	16.8 ± 0.3	16.6 ± 0.4	1.0 ± 0.3	0.9 ± 0.2
PE	17.4 ± 0.2	17.4 ± 0.3	1.3 ± 0.3	1.1 ± 0.3
PG	17.1 ± 1.1	16.8 ± 0.9	1.4 ± 0.4	1.2 ± 0.3
PI	17.5 ± 0.2	17.2 ± 0.2	1.4 ± 0.3	1.2 ± 0.3
PS	17.9 ± 0.1	18.1 ± 0.2	1.5 ± 0.3	1.2 ± 0.2
LPC	17.3 ± 0.5	17.1 ± 0.7	1.4 ± 0.5	1.1 ± 0.4
**Ether neutral lipids**				
TGe acyl chains	16.9 ± 0.7	17.2 ± 0.3	1.0 ± 0.3	0.6 ± 0.2
TGe alkyl chain	18.5 ± 1.2	18.8 ± 0.5	0.4 ± 0.5	0.7 ± 0.6
**Ether phospholipids**				
PCe acyl chain	14	14	1	1
PCe alkyl chain	20	20	0	0
PEe acyl chain	21.3 ± 1.9	20.2 ± 1.6	2.3 ± 0.8	1.8 ± 0.4
PEe alkyl chain	15.5 ± 2.4	16.2 ± 1.9	0.1 ± 0.2	0.2 ± 0.2
PEp acyl chain	22.5 ± 1.6	23 ± 1.4	1.4 ± 0.6	1.0 ± 0.6
PEp alkenyl chain	13.3 ± 1.7	12.8 ± 1.5	0.0 ± 0.0	0.0 ± 0.0

**Table 3:** Large differences between observed and expected frequencies of combinations of acyl chains in the nine joint carbon/double bond content categories in each ester lipid class. Differences are first expressed as ratios (± SE) of the percentages of observed vs. expected, with the absolute difference given in brackets. Abbreviations for the categories of combinations and large differences are as defined in the text. Lipid categories are shown in bold.

Class	Excess			Deficit		
	Combination	Day 1	Day 19	Combination	Day 1	Day 19
**Neutral lipids**						
DG	S0_LX	27.4 ± 10.8 (7)	9.6 ± 1.3 (7.5)	M0_M1	0.1 ± 0.1 (−10.9)	0.7 ± 0.3 (−2.4)
	M1_L0	1.9 ± 0.3 (9)	2.4 ± 0.3 (7.6)			
TG	S0_M0_M1	1.4 ± 0.2 (1.8)	1.5 ± 0.1 (3.7)	M0_M1_MX	0.3 ± 0.1 (−5.6)	0.9 ± 0.1 (−1.2)
	M0_M0_M1	1.6 ± 0.2 (8.2)	1.3 ± 0.2 (6.4)			
**Phospholipids**						
CL	S0_M1_MX_LX	5.2 ± 0.4 (5.4)	6.1 ± 0.9 (4.5)	M1_MX_MX_LX	0.2 ± 0.0 (−6.8)	0.3 ± 0.0 (−3.8)
	MX_MX_MX_MX	2.1 ± 0.1 (13.5)	3.4 ± 0.2 (5.9)			
PC	M0_MX	1.5 ± 0.0 (9.8)	1.7 ± 0.1 (2.7)	M0_M0	0.4 ± 0.0 (−9.1)	0.6 ± 0.0 (−2.4)
				M1_MX	0.3 ± 0.0 (−12)	0.5 ± 0.0 (−5.1)
PE	M0_MX	1.4 ± 0.0 (8.7)	2.3 ± 0.1 (20.9)	M0_M0	0.1 ± 0.0 (−9.5)	0.0 ± 0.0 (−9.3)
				M1_MX	0.7 ± 0.0 (−7.4)	0.4 ± 0.0 (−12.6)
				MX_MX	0.9 ± 0.1 (−0.7)	0.5 ± 0.0 (−3.2)
PG	SX_LX	9.7 ± 0.9 (18.4)	10.6 ± 0.7 (17.1)	SX_M0	0.0 ± 0.0 (−6.5)	0.0 ± 0.0 (−6)
	M0_M1	1.5 ± 0.1 (4.6)	1.8 ± 0.1 (13.4)	M0_M0	0.0 ± 0.0 (−9.9)	0.0 ± 0.0 (−10.1)
	M0_MX	2.3 ± 0.1 (28.1)	2.3 ± 0.1 (18.9)	M0_LX	0.0 ± 0.0 (−6.5)	0.0 ± 0.0 (−6)
				MX_MX	0.2 ± 0.0 (−9.3)	0.2 ± 0.0 (−4.3)
PI	M0_MX	1.6 ± 0.1 (17.1)	1.6 ± 0.1 (15.4)	M0_M0	0.0 ± 0.0 (−9.4)	0.0 ± 0.0 (−12.3)
				M1_M1	0.2 ± 0.0 (−5.4)	0.7 ± 0.0 (−3.1)
				MX_MX	0.3 ± 0.0 (−13.1)	0.5 ± 0.0 (−6.1)
PS	M0_M1	1.8 ± 0.1 (11.7)	1.7 ± 0.1 (7.4)			
	MX_MX	1.4 ± 0.1 (4.6)	2.5 ± 0.2 (7.4)			

**Table 4:** Gene–enzyme systems in the Kennedy, ACYL-DHAP and Lands’ pathways with direct effects on the identities of the acyl chains at the *sn1*, *sn2* and *sn3* positions. Enzymes are primarily classified on the basis of their Enzyme Commission (EC) numbers. Mammalian and insect gene–enzyme systems are represented by *H. sapiens* and *D. melanogaster*, respectively, and the top blastp matches for the latter in the *B. tryoni* reference proteins are also given. Human reference sequences for GPATs, LPLATs and DGAT1 reported in Valentine et al. [7] were used to identify their top blastp matches in *D. melanogaster*. Additional members of these enzyme classifications in *D. melanogaster* were extracted from the “Gene Group Lists” in Flybase (https://flybase.org/lists/FBgg/; 17 November 2022, Garapati et al. [132]). Dashes indicate the absence of identifiable orthologous sequences. Interspecific pairwise amino acid identity was calculated based on the optimal overall alignment between two full-length homologues using EMBOSS Needle (https://www.ebi.ac.uk/Tools/psa/emboss_needle/; 17 November 2022). See Table S16 for further details. *Hs* = *Homo sapiens*, *Dm* = *Drosophila melanogaster, Bt* = *Bactrocera tryoni*. Fine lines are used to help distinguish 1:1 relationship between enzymes/isoforms in the different species from cases involving more complex relationships.

Enzyme (EC,*sn*#)	*H. sapiens*	*D. melanogaster*		*B. tryoni*		
	*Hs* gene-isoform (NM#)	*Dm* gene-isoform	*Dm*/*Hs* % ID	*Bt* gene (LOC#)	*Bt* isoform (XP#)	*Dm*/*Bt* % ID
GPAT (2.3.1.15,*sn1*)	GPAT4 (178819)	Gpat4-PB	46	120770141	39953264	77
	GPAT3 (001256421)	Gpat4-PC	46		39953263	74
		CG15450-PA	39		39953264	37
	GPAT1 (001244949)	mino-PA	25	120777159	39964647	62
	GPAT2 (207328)	mino-PC	21		39965461	63
DHAPAT (2.3.1.42,*sn1*)	GNPAT (001316350)	Gnpat-PC	23	120775111	39961065	57
ADHAP-S (2.5.1.26,*sn1*)	AGPS (003659)	ADPS-PA	47	120771366	39955259	66
AGPAT/AAGPAT (2.3.1.51,*sn2*)	LPLAP1/APGAT1 (032741)	Agpat1-PA	32	120775056	39960970	68
	LPLAP2/APGAT2 (006412)		28			
	-	Agpat2-PD	-	120781541	39969707	62
	LPLAP3/APGAT3 (001037553)	Agpat3-PE	39	120777449	39964700	61
	LPLAP4/APGAT4 (020133)		37			
	LPLAP5/APGAT5 (018361)	Agpat4-PB	22			57
DGAT (2.3.1.20,*sn3*)	DGAT1 (012079.6)	mdy-PE	34	120767901	39950180	76
	MOGAT2 (025098)	Dgat2-PA	38	120772092	39956430	62
		CG1941-PC	36			62
		CG1946-PA	38			62
Delta-1-desaturase (1.14.19.77,*sn1*)	PEDS1/TMEM189 (199129)	Kua-PA	48	120768982	39951731	69
PLA1 (3.1.1.32,*sn1*)	DDHD2 (015214)	PAPLA1-PD	14	120775130	39961092	43
PLA2 (3.1.1.4,*sn2*) Ca2+ independent	PLB1 (001170585)	CG7365-PA	10	120778470	39966215	63
		CG11029-PA	10	120769296	39952159	46
	PLA2G6 (001004426)	iPLA2-VIA-PB	32	120777471	39964729	77
PLA2 (3.1.1.4,*sn2*) secreted	-	CG3009-PD	-	120775860	39962164	79
	PLA2G1B (000928)	CG14507-PC	13	120773488	39958366	44
	-	CG30503-PA	-	120771404	39955313	40
	-	CG42237-PA	-	120775514	39961656	60
	PLA2G3 (015715)	GIIIspla2-PB	15	120774912	39960718	31
	PLA2G12A (030821)	GXIVsPLA2-PA	22	120779188	39967384	72
	-	sPLA2-PB	-	120771404	39955313	37
PLB (3.1.1.5,*sn1,2*)	OVCA2 (080822)	CG5412-PA	28	120773391	39958163	70
	ABHD12 (015600)	CG15111-PA	31	120772418	39956961	61
	PNPLA7 (152286)	sws-PA	40	120773795	39958830	64
LPLAT (2.3.1.-,*sn1*)	LPLAP6/LCLAT1 (182551)	Agpat4-PB	24	120777449	39964700	57
	LPLAP7/LPGAT1 (014873)	-	-	-	-	-
	LPLAP8/LPCAT1 (024830)	LPCAT-PB	33	120775046	39960955	67
	LPLAP9/LPCAT2 (017839)		33			
	LPLAP10/LPCAT4 (153613)		30			
	LPLAP11/MBOAT7 (024298)	frj-PB	32	120769360	39952255	69
	LPLAP12/LPCAT3 (005768)	nes-PA	34	120776802	39963730	52
	LPLAP13/MBOAT2 (138799)	oys-PA	29	120772337	39956816	69
	LPLAP14/MBOAT1(001080480)		27			

## Data Availability

Raw data and codes for the association test are provided at https://github.com/shirleen20/Qfly_FA_chains_MS, 5 September 2023.

## Supplementary Materials

The following supporting information can be downloaded at: https://www.mdpi.com/article/10.3390/insects14110873/s1, Supplementary Dataset S1: Peak areas of different compounds detected in each class in Day 1 and Day 19 males, Figure S1: Correlations between the abundances and diversities of the various lipid classes in Day 1 and Day 19 males, Table S1: Standards and regression equations used to convert lipid peak areas to concentrations, Table S2: Lipid species found in each class and day in males, Table S3: Comparison of lipid diversities in *B. tryoni* with those reported in lipidomic analyses of *D. melanogaster*, the mosquito *Aedes aegypti* and the moth *Bombyx mori*, Table S4: Neutral lipids and their percentage abundances and numbers of replicates in which they were found on both days, Table S5: Phospholipids and their percentage abundances and numbers of replicates in which they were found on both days. Table S6: Ether lipids and their percentage abundances and numbers of replicates in which they were found on both days, Table S7: Comparison of lipid compositions in *B. tryoni* with those reported for *D. melanogaster* and the lepidopterans *B. mori* and *Samia cynthia*, Table S8: Comparison of mean carbon chain lengths and double bonds (before and after the dash, respectively) of acyl chains in ester lipid classes of *B. tryoni* males with those reported for *D. melanogaster*, Table S9: Common chains and their percentage abundances in ester lipids in Day 1 and Day 19 males, Table S10: Common acyl and alkyl/alkenyl chains and their percentage abundances in ether lipids in Day 1 and Day 19 males, Table S11: Comparison of the relative abundances of different acyl chains in ester neutral lipids of *B. tryoni* with their abundances in two other tephritids, *B. oleae* and *C. capitata*, plus *D. melanogaster*, the mosquito *Aedes cinerius* and the lepidopteran *S. cynthia*, Table S12: Comparison of the relative abundances of different acyl chains in ester phospholipids of *B. tryoni* with their abundances in two other tephritids, *B. oleae* and *C. capitata*, plus *D. melanogaster*, the mosquito *Aedes cinerius* and the lepidopteran *S. cynthia*. Table S13: Differences between observed and expected frequencies of combinations of acyl chains in the nine joint carbon length/double bond categories in each ester lipid class, Table S14: Differences between observed and expected frequencies of combinations of acyl chains in the three chain length categories in each ester lipid class, Table S15: Differences between observed and expected frequencies of combinations of acyl chains in the three double bond categories in each ester lipid class, Table S16: Gene–enzyme systems in the Kennedy, ACYL-DHAP and Lands’ pathways with direct effects on the identities of the acyl chains at the *sn1*, *sn2* and *sn3* positions.
