# Correction: Prasad et al. Patterns of Variation in the Usage of Fatty Acid Chains among Classes of Ester and Ether Neutral Lipids and Phospholipids in the Queensland Fruit Fly. *Insects* 2023, *14*, 873

**DOI:** 10.3390/insects15070538

**Published:** 2024-07-18

**Authors:** Shirleen S. Prasad, Matthew C. Taylor, Valentina Colombo, Heng Lin Yeap, Gunjan Pandey, Siu Fai Lee, Phillip W. Taylor, John G. Oakeshott

**Affiliations:** 1 Environment, Commonwealth Scientific and Industrial Research Organisation, Black Mountain, Acton, ACT 2601, Australia; shirleen.prasad@csiro.au (S.S.P.); m.taylor@csiro.au (M.C.T.); vn.colombo@gmail.com (V.C.); henglin.yeap@csiro.au (H.L.Y.); siu.f.lee@gmail.com (S.F.L.); johnoakeshott3@gmail.com (J.G.O.); 2 Applied BioSciences, Macquarie University, North Ryde, NSW 2109, Australia; phil.taylor@mq.edu.au; 3 Australian Research Council Centre for Fruit Fly Biosecurity Innovation, Macquarie University, North Ryde, NSW 2109, Australia; 4 Health and Biosecurity, Commonwealth Scientific and Industrial Research Organisation, Parkville, VIC 3052, Australia; 5 Bio21 Molecular Science and Biotechnology Institute, University of Melbourne, Parkville, VIC 3052, Australia

## Error in Table

Supplementary Table S3 in our recent publication [1] contained errors arising from the misinterpretation of a supplementary data file in another publication [2].

## Text Correction

As a consequence of the corrections made to Table S3, three main text sentences have also been revised.

In the original paper, Section 3. Results, Sub-section 3.1. Overall Lipidome Compositions, Paragraph 2, the sentence “While fewer ether lipids were detected than in the *D. melanogaster* studies, and only about a third were chain-specified, many more ether lipids were chain-specified in Qfly than in the *D. melanogaster* precedents” has been modified in the revised version to “Little definitive data on the ether lipids were available in the *D. melanogaster* studies, but fewer TGes were detected in our study than in the one *D. melanogaster* study which could be compared. Only about two-thirds of the Qfly ether lipids were chain-specified, but this was many more than in the *D. melanogaster* precedents”.

In the original paper, Section 3. Results, Sub-section 3.5. Possible Sampling Biases in the Ether Lipid Data, Paragraph 2, the sentence “The scope for detection bias was significant, given that the total number of ether lipids we recovered, 38, was only about a third as many as have so far been reported in *D. melanogaster*, even though those in the latter were seldom chain-specified [51,52].” has been modified in the revised version to “The scope for detection bias was significant, given that the total number of ether lipids we recovered, 38, may be significantly fewer than the available *D. melanogaster* data suggest may exist (noting that the latter have seldom been chain-specified [51,52])”.

In the original paper, Section 4. Discussion, Paragraph 9, the sentence “We only recovered about a third as many ether lipids as have so far been reported in *D. melanogaster* [51,52,54], whereas our numbers of chain-specified ether lipids, 24 in total, were significantly higher than the precedents in that species, or any other insects [97,98].” has been changed to “We only recovered a proportion of the ether lipids that studies so far suggest may exist in *D. melanogaster* [51,52,54], whereas our numbers of chain-specified ether lipids, 24 in total, were significantly higher than the precedents in that species or any other insects [97,98]”.

The authors state that the scientific conclusions are unaffected. This correction was approved by the Academic Editor. The original publication has also been updated.
