# Supplementary material for: Patterns of Variation in the Usage of Fatty Acid Chains among Classes of Ester and Ether Neutral Lipids and Phospholipids in the Queensland Fruit Fly
Source: Insects. 2023 Nov 14;14(11):873. doi: 10.3390/insects14110873 (PMC10672513; doi:10.3390/insects14110873)

Supplementary Tables and Figures

Title

Patterns of variation in the usage of fatty acid chains among classes of ester and ether neutral and phospholipids in the Queensland fruit fly

Authors

Shirleen S. Prasad[1,2,3], Matthew C. Taylor[1], Valentina Colombo[1], Heng Lin Yeap[1,4,5], Gunjan Pandey[1,2], Siu Fai Lee[1,2,3], Phillip W. Taylor[2,3] and John G. Oakeshott[1,2]

Affiliations

[1]Environment, Commonwealth Scientific and Industrial Research Organisation, Black Mountain, Acton, ACT 2601, Australia

[2]Applied BioSciences, Macquarie University, North Ryde, NSW 2109, Australia

[3]Australian Research Council Centre for Fruit Fly Biosecurity Innovation, Macquarie University, North Ryde, NSW 2109, Australia

[4]Health and Biosecurity, Commonwealth Scientific and Industrial Research Organisation, Parkville, VIC 3052, Australia.

[5]Bio21 Molecular Science and Biotechnology Institute, University of Melbourne, Parkville, VIC 3052, Australia

Corresponding Author

Gunjan Pandey

Gunjan.Pandey@csiro.au

Table S1: **Standards and regression equations used to convert lipid peak areas to concentrations.** Units for the concentrations tested were ng/µl, which were $\log_{10}$ transformed for the regressions. The arbitrary units for the peak areas were also $\log_{10}$ transformed for the regressions.

Class	Compounds tested	Concentrations tested	Regression statistics		
			Intercept	Slope	R2
**DG**	18:1_18:1	0.1, 1, 10, 50	5.58	1.33	0.98
**TG**	18:1_18:1_18:1	0.1, 1, 10, 50	6.79	0.96	0.98
**CL**	72:4	0.01, 0.1, 1, 10	6.00	1.52	0.99
**PC**	18:1_18:1	0.1, 1, 10, 50	6.78	1.17	0.97
**PE**	16:0_18:1	0.1, 1, 10, 50	5.98	1.18	0.98
**PG**	18:0_18:1	0.1, 1, 10, 50	5.25	1.18	0.97
**PI**	34:2	0.1, 1, 10, 50	5.68	1.23	0.99
**PS**	34:0_17:0	0.1, 1, 10, 50	5.73	1.23	0.98
**LPC**	18:1	0.1, 1, 10, 50	6.37	1.21	0.98
**PEp**	18:0_18:1	0.1, 1, 10, 50	6.18	1.23	0.98

Table S2: Lipid species found in each class and Day in males. Species found in both Days are shown in black, and those found in Day 1 only and Day 19 only are shown in blue and red, respectively. The chains in each lipid are listed in ascending order of side chain sizes. Note that in some cases the identities of individual chains could not be unambiguously determined, so just the total numbers of carbons and double bonds summed across the component chains are given.

**Neutral lipids**								
DG	TG	TG	TG	TG	TG	TG	TG	TG
4:0_20:4	4:0_11:1_21:0	4:0_18:1_16:0	9:0_18:1_16:0	15:0_16:0_16:1	16:0_16:0_16:1	16:0_18:1_6:0	16:1_11:1_18:2	16:1_18:1_18:1
16:0_16:1	4:0_11:1_23:0	4:0_18:1_16:1	10:0_18:1_16:0	16:0_10:0_18:1	16:0_16:0_18:1	16:0_18:2_10:0	16:1_12:0_16:0	16:1_18:1_24:0
16:0_18:1	4:0_14:0_16:0	4:0_18:1_17:0	10:0_18:3_16:0	16:0_10:3_18:1	16:0_16:0_18:2	16:0_18:2_10:3	16:1_12:0_16:1	16:1_18:1_6:0
16:0_18:2	4:0_14:1_16:0	4:0_18:1_18:0	11:0_10:0_22:6	16:0_11:1_18:1	16:0_16:1_13:0	16:0_18:2_13:0	16:1_12:2_11:3	16:1_18:1_8:0
16:0_18:3	4:0_14:1_16:1	4:0_18:1_18:2	11:0_12:2_17:0	16:0_11:2_14:3	16:0_16:1_14:0	16:0_18:2_18:1	16:1_14:1_16:1	16:1_18:2_18:2
16:1_16:1	4:0_14:2_22:3	4:0_18:1_18:4	12:0_11:4_20:4	16:0_11:2_21:0	16:0_16:1_16:0	16:0_18:3_10:0	16:1_14:4_11:2	16:1_18:3_11:1
16:1_18:1	4:0_16:0_16:0	4:0_18:1_20:0	12:0_18:2_16:0	16:0_11:3_12:2	16:0_16:1_16:1	16:0_18:3_11:1	16:1_16:1_16:0	16:1_18:3_18:3
16:1_18:2	4:0_16:0_18:0	4:0_18:2_16:1	12:0_18:2_18:2	16:0_11:4_14:2	16:0_16:1_18:1	16:0_18:3_12:0	16:1_16:1_16:1	16:1_6:0_18:2
16:1_20:4	4:0_16:0_18:1	4:0_18:2_18:2	12:0_18:3_18:2	16:0_11:4_14:3	16:0_16:1_8:0	16:0_18:3_18:3	16:1_16:1_17:1	16:2_10:4_16:0
18:0_18:1	4:0_16:0_18:3	4:0_18:2_18:3	14:0_11:3_12:2	16:0_12:2_11:2	16:0_18:1_10:2	16:0_18:3_20:2	16:1_16:1_18:1	16:2_11:4_16:1
18:0_18:2	4:0_16:0_20:5	4:0_18:3_16:1	14:0_14:0_14:1	16:0_12:2_11:3	16:0_18:1_10:3	16:0_23:1_18:1	16:1_16:1_18:2	16:3_11:3_18:1
18:1_18:1	4:0_16:0_22:5	4:0_18:3_18:0	14:0_14:0_16:0	16:0_14:0_12:0	16:0_18:1_16:1	16:0_6:0_18:1	16:1_16:1_18:3	16:4_11:1_18:0
18:1_18:2	4:0_16:1_14:0	4:0_18:3_18:3	14:0_18:2_11:1	16:0_14:0_14:1	16:0_18:1_18:0	16:0_8:0_16:1	16:1_17:0_16:0	16:4_11:3_18:1
18:1_18:3	4:0_16:1_16:0	6:0_11:3_23:0	14:1_11:4_18:0	16:0_14:0_16:0	16:0_18:1_18:1	16:0_9:0_11:2	16:1_17:1_16:0	16:4_19:0_19:0
18:1_20:5	4:0_16:1_16:1	6:0_12:1_18:3	14:1_11:4_18:1	16:0_14:2_11:3	16:0_18:1_18:2	16:1_10:0_16:0	16:1_18:1_12:0	16:4_6:0_20:0
18:2_18:3	4:0_16:1_18:1	6:0_18:1_18:2	15:0_10:2_13:0	16:0_14:4_11:2	16:0_18:1_21:0	16:1_10:0_16:1	16:1_18:1_16:0	17:0_18:1_16:0
20:0_16:1	4:0_16:1_18:3	8:0_11:2_22:6	15:0_11:3_14:3	16:0_16:0_16:0	16:0_18:1_22:0	16:1_11:1_16:0	16:1_18:1_17:1	17:1_14:4_11:2
**Neutral lipids**					**Phospholipids**			
TG	TG	TG	TG	TG	CL	CL	CL	PC
18:0_11:2_14:3	18:1_12:2_11:3	18:2_11:1_18:0	18:4_16:1_16:1	26:0_16:0_18:1	14:0_20:4_16:1_16:1	18:3_18:2_14:0_22:6	74:14#	6:0_22:1
18:0_12:2_11:2	18:1_12:2_12:2	18:2_11:1_18:1	18:4_6:0_16:0	26:0_16:0_24:1	16:1_16:1_16:1_16:1	18:3_18:2_14:1_22:6	74:15#	6:0_24:2
18:0_16:0_16:0	18:1_14:0_14:0	18:2_11:4_14:3	19:0_12:2_18:2	26:1_16:0_18:1	18:1_16:1_16:1_16:1	18:3_18:2_16:1_18:2		14:0_18:3
18:0_16:0_18:0	18:1_14:4_11:2	18:2_12:2_12:2	19:0_18:1_16:0	26:1_18:1_16:1	18:1_16:1_16:1_18:1	18:3_18:2_18:2_18:2		16:0_14:0

18:0_16:0_18:2	18:1_17:1_16:0	18:2_14:2_11:4	19:0_19:0_14:4	26:1_18:1_18:1	18:1_16:1_18:2_18:2	18:4_16:1_14:0_20:4		16:0_16:0
18:0_16:0_20:4	18:1_18:1_16:0	18:2_17:1_16:0	20:0_10:2_11:4	28:0_16:0_18:1	18:2_14:1_22:6_18:2	18:4_16:1_16:1_16:1		16:0_16:1
18:0_18:0_16:0	18:1_18:1_17:0	18:2_17:1_16:1	20:0_11:3_14:2	28:0_18:1_18:1	18:2_16:0_16:1_18:2	18:4_16:1_16:1_18:1		16:0_18:1
18:0_18:0_18:1	18:1_18:1_17:1	18:2_17:1_18:1	20:0_14:3_11:3	28:1_18:1_16:0	18:2_16:1_16:1_16:1	18:4_18:2_16:1_16:1		16:0_18:2
18:0_18:0_18:2	18:1_18:1_18:0	18:2_18:2_18:2	20:0_16:0_16:1	28:1_18:1_18:1	18:2_16:1_16:1_18:2	18:4_18:2_16:1_18:2		16:0_18:3
18:0_18:1_16:0	18:1_18:1_18:1	18:3_12:2_11:3	20:0_16:0_18:1	30:0_18:1_16:0	18:2_16:1_18:2_18:2	18:4_18:2_16:1_22:6		16:0_20:4
18:0_18:1_20:0	18:1_18:1_18:2	18:3_13:0_12:4	20:0_18:2_18:1	41:6#	18:2_18:2_16:1_18:1	18:4_18:2_18:2_18:2		16:1_14:0
18:0_18:3_20:1	18:1_18:1_20:0	18:3_18:2_18:2	20:1_18:1_18:3		18:2_18:2_18:2_18:2	20:5_18:2_16:1_18:2		16:1_16:1
18:1_11:1_16:0	18:1_18:1_6:0	18:3_18:3_18:1	20:5_11:2_12:2		18:3_16:1_14:0_20:4	22:6_14:1_16:1_18:2		16:1_18:1
18:1_11:1_18:1	18:1_18:2_6:0	18:3_18:3_18:2	20:5_11:3_12:2		18:3_16:1_14:0_22:6	70:11#		16:1_18:3
18:1_11:2_14:2	18:1_18:3_11:1	18:4_11:3_14:4	21:1_12:2_12:4		18:3_16:1_16:1_20:4	72:12#		17:0_18:1
18:1_11:2_18:1	18:1_24:0_16:0	18:4_14:0_16:1	22:4_10:1_11:3		18:3_16:1_18:2_16:1	72:13#		17:0_18:3
18:1_11:3_14:4	18:2_11:1_16:0	18:4_16:0_16:1	25:0_6:0_11:1		18:3_18:1_18:2_18:2	74:12#		17:1_18:1
**Phospholipids**								
PC	PE	PE	PE	PG	PI	PI	PS	PS
18:0_16:0	16:0_16:0	17:1_18:1	20:0_18:1	16:0_16:1	16:0_16:1	18:1_18:3	16:0_18:1	18:3_18:3
18:0_18:1	16:0_16:1	18:0_14:3	20:0_18:2	16:0_18:1	16:0_18:1	18:3_18:2	16:1_20:1	18:3_20:5
18:3_18:2	16:0_18:1	18:0_16:0	20:0_18:3	16:0_18:2	16:0_18:2	18:3_18:3	16:2_18:0	19:0_18:1
18:3_18:3	16:0_18:2	18:0_18:1	25:1_11:0	16:0_18:3	16:0_18:3	30:1#	18:0_18:1	33:00:00
18:4_16:0	16:0_18:3	18:0_18:3	38:7#	16:1_16:1	16:1_16:1		18:0_18:3	34:04:00
18:4_18:1	16:0_22:6	18:1_18:1		16:1_18:3	16:1_18:3		18:1_18:1	38:05:00
25:1_8:0	16:1_16:1	18:1_18:3		18:1_18:3	16:1_18:3		18:1_18:2	38:06:00
33:04:00	16:1_18:2	18:1_21:1		18:3_18:3	18:0_18:1		18:1_18:3	39:01:00
33:08:00	16:1_18:3	18:3_18:2		24:2_10:2	18:0_18:2		18:1_20:2	39:03:00
34:05:00	16:1_20:1	18:3_18:3			18:0_18:3		18:1_20:4	39:05:00
34:06:00	17:0_18:1	18:4_20:5			18:1_18:2		18:3_18:2	
**Phospholipids**		**Ether neutral lipids**		**Ether phospholipids**				
LPC	LPC	DGe	TGe	PCe	PEe	PEe	PEp	PSe
12:0	18:2	22:3e	14:1e_11:3_18:2	20:0e_14:1	12:0e_24:2	38:2e	10:0p_24:0	38:5e

14:0	18:3	32:3e	18:0e_16:0_16:1	16:0e_22:6	38:3e	12:0p_24:0
14:1	18:4	34:3e	18:0e_16:0_18:1	16:1e_18:1	42:3e	12:0p_24:2
16:0	20:1	34:4e	18:0e_16:1_18:1	18:0e_18:2		18:0p_16:1
16:1	20:4	34:5e	18:3e_17:0_19:0	18:2e_19:0		18:0p_18:1
16:2		36:3e	20:0e_16:0_18:1	20:0e_18:1		18:0p_18:2
17:0			20:0e_16:1_18:1	20:0e_18:2		18:0p_18:3
17:1			20:0e_18:1_18:1	20:0e_18:3		
18:0			32:1e	36:0e		
18:1			34:2e	37:3e		

Table S3: Comparison of lipid diversities in *B. tryoni* with those reported in lipidomic analyses of *D. melanogaster*, the mosquito *Aedes aegypti* and the moth *Bombyx mori*. The dash indicates the class was not reported in the study. The values in brackets for *B. tryoni* refer to the diversities of disambiguated chains only. "Other lipids" includes various cholesterols, prenols (e.g. ubiquinone) and fatty acyls (e.g. carnitine). LPE = lysophosphatidylethanolamine, LPG = lysophosphatidylglycerol, LPI = lysophosphatidylinositol, LPA = lysophosphatidic acid, LPS = lysophosphatidylserine, Sph = sphingosine, Cer = ceramide, CerPE = ceramide phosphatidylethanolamine, SM = sphingomyelin, PCp = alkenyl phosphatidylcholine, PGe = alkyl phosphatidylglycerol, PIe = alkyl phosphatidylinositol, PIp = alkenyl phosphatidylinositol and PSp = alkenyl phosphatidylserine. *

Class	Present study		Carvalho *et al.* [51]		Guan *et al.* [52]		Colinet *et al.* [53]	Ko *et al.* [54]	Hofbauer *et al.* [43]	Pinch *et al.* [58]	Zhu *et al.* [61]
	B. tryoni (whole body extract)		*D. melanogaster (whole body extract)*							*A. aegypti (25 fat bodies)*	*B. mori (whole body extract)*
	**Day 1 ♂**	**Day 19 ♂**	**Day 9 ♀**	**Day 9 ♂**	**Day 1 ♂**	**Day 20 ♂**	**Day 6 ♀**	**Virgin ♀**	**Day 7 ♂**	**Day 7-10 ♀**	**Larvae**
**Neutral lipids**	**213**	**196**	**89**	**88**	**55**	**40**	**1**	**46**	**149**	**52**	**179**
DG	11 (9)	16 (11)	16	15	—	—	—	21	19	9	49
TG	202 (129)	180 (126)	73	73	55	40	—	25	130	43	126
Others	—	—	—	—	—	—	1	—	—	—	4
**Phospholipids**	**125**	**148**	**86**	**85**	**169-232**	**169-232**	**100**	**198**	**69**	**54**	**326**
CL	36 (27)	34 (26)	—	—	13	13	—	—	—	1	1
PC	26 (24)	27 (25)	23	23	29-53	29-53	23	39	30	10	100
PE	19 (19)	27 (25)	28	28	36-63	36-63	22	39	23	17	97
PG	9 (8)	9 (8)	8	7	17	17	14	36	—	2	25
PI	12 (11)	15 (13)	14	14	27-39	27-39	15	27	10	8	32
PS	9 (7)	21 (18)	11	11	29	29	7	26	6	—	42
PA	—	—	2	2	18	18	—	9	—	3	14
LPC	14 (14)	15 (15)	—	—	—	—	6	12	—	4	—
LPE	—	—	—	—	—	—	7	10	—	3	—
LPG	—	—	—	—	—	—	3	—	—	2	—
LPI	—	—	—	—	—	—	3	—	—	2	—
LPA	—	—	—	—	—	—	—	—	—	1	—

LPS	–	–	–	–	–	–	–	–	–	1	–
Others	–	–	–	–	–	–	–	–	–	–	15
**Sphingolipids**	**0**	**0**	**40**	**41**	**156**	**152**	**0**	**0**	**0**	**5**	**111**
Sph	–	–	–	–	–	–	–	–	–	1	4
Cer	–	–	27	27	63	63	–	–	–	3	30
Cer PE	–	–	13	14	93	89	–	–	–	–	37
SM	–	–	–	–	–	–	–	–	–	1	23
Others	–	–	–	–	–	–	–	–	–	–	17
**Ether neutral lipids**	**15**	**15**	**0**	**0**	**0**	**0**	**0**	**24**	**0**	**–**	**1**
DGe	6 (6)	6 (6)	–	–	–	–	–	–	–	–	1
TGe	9 (9)	9 (9)	–	–	–	–	–	24	–	–	–
**Ether phospholipids**	**0**	**0**	**11**	**12**	**≤110**	**≤110**	**0**	**0**	**0**	**0**	**64**
PCe	1 (1)	1 (1)	–	–	≤24	≤24	–	–	–	–	15
PCp	–	–	–	–	≤18	≤18	–	–	–	–	3
PEe	13 (10)	11 (8)	11	12	≤27	≤27	–	–	–	–	20
PEp	7 (6)	6 (6)	–	–	≤20	≤20	–	–	–	–	15
PGe	–	–	–	–	–	–	–	–	–	–	2
PIe	–	–	–	–	≤12	≤12	–	–	–	–	2
PIp	–	–	–	–	≤9	≤9	–	–	–	–	2
PSe	1 (1)	1 (1)	–	–	–	–	–	–	–	–	3
PSp	–	–	–	–	–	–	–	–	–	–	2
**Other lipids**	**–**	**–**	**–**	**–**	**12**	**12**	**–**	**–**	**–**	**12**	**428**
**SUM**	**375**	**378**	**247**	**247**	**≤502-565**	**≤483-546**	**101**	**268**	**218**	**117**	**905**

*The analytical software used by Guan *et al*. [52] and Scheitz *et al*. [30] recognised extensive ambiguities between several, mainly ester and ether phospholipid, classes. We therefore only present the data for Guan *et al*. [52] and show a range of values from that study for the classes in question. The data for Tuthill *et al.* [50] are not included in this and other relevant supplementary tables below because they were based on analyses of individual tissues.

Table S4: Neutral lipids and their percentage abundances and numbers of replicates in which they were found on both Days. The data for species found in both Days are shown in black, with the Day 1 and Day 19 abundance data before and after the slash, respectively, and those found in Day 1 only and Day 19 only are shown in blue and red, respectively.

DG		TG		TG		TG		TG		TG	
**Identity**	**Abundance**	**Identity**	**Abundance**	**Identity**	**Abundance**	**Identity**	**Abundance**	**Identity**	**Abundance**	**Identity**	**Abundance**
4:0_20:4	0.02,7/0.03,16	10:0_18:1_16:0	0.08,4/0.49,10	16:0_10:0_18:1	<0.01,1/<0.01,4	16:0_16:1_16:1	1.46,7/1.79,6	16:0_18:3_20:2	0.51,10/0.04,16	16:1_18:1_24:0	0.05,15/0.13,16
16:0_18:2	0.10,6/0.03,7	10:0_18:3_16:0	0.06,13/0.01,13	16:0_10:3_18:1	0.03,15/<0.01,12	16:0_16:1_18:1	<0.01,2/<0.01,1	16:0_23:1_18:1	0.06,15/0.02,16	16:1_18:1_6:0	0.56,15/0.44,15
16:0_18:3	0.01,1/0.02,6	11:0_10:0_22:6	0.01,9/<0.01,6	16:0_11:1_18:1	<0.01,3/<0.01,2	16:0_16:1_8:0	0.01,3/0.14,11	16:0_9:0_11:2	0.04,9/0.02,2	16:1_18:1_8:0	0.12,15/0.04,16
16:1_16:1	0.08,12/0.03,6	11:0_12:2_17:0	<0.01,6/0.01,4	16:0_11:2_14:3	<0.01,1/0.01,16	16:0_18:1_10:2	0.01,6/<0.01,6	16:1_10:0_16:1	<0.01,5/0.02,15	16:1_18:2_18:2	0.67,10/0.05,15
16:1_18:1	0.03,7/0.01,3	12:0_11:4_20:4	0.04,10/<0.01,16	16:0_12:2_11:2	0.05,14/<0.01,3	16:0_18:1_16:1	<0.01,15/<0.01,10	16:1_11:1_16:0	0.19,12/0.02,16	16:1_18:3_18:3	0.03,15/<0.01,9
18:0_18:1	<0.01,2/0.01,7	12:0_18:2_16:0	0.83,13/0.92,10	16:0_12:2_11:3	0.10,3/0.01,15	16:0_18:1_18:1	3.78,12/2.59,14	16:1_12:2_11:3	0.09,15/<0.01,11	16:1_6:0_18:2	0.01,14/<0.01,4
18:0_18:2	0.01,9/0.01,4	12:0_18:2_18:2	0.20,15/0.03,16	16:0_14:0_12:0	0.02,12/0.03,16	16:0_18:1_18:2	<0.01,4/1.84,16	16:1_16:1_16:0	<0.01,5/<0.01,4	16:3_11:3_18:1	0.01,15/<0.01,1
18:1_18:3	0.06,9/0.03,12	12:0_18:3_18:2	0.03,15/<0.01,12	16:0_14:0_16:0	0.03,13/0.20,15	16:0_18:1_21:0	0.10,15/0.05,16	16:1_16:1_16:1	1.06,15/0.39,4	16:4_11:1_18:0	0.01,15/<0.01,2
18:2_18:3	<0.01,1/0.01,15	14:0_11:3_12:2	0.02,15/<0.01,4	16:0_14:2_11:3	0.01,13/<0.01,14	16:0_18:1_22:0	0.20,15/0.12,15	16:1_16:1_17:1	0.16,15/0.06,16	16:4_19:0_19:0	0.66,15/0.05,16
20:0_16:1	0.06,15/0.04,16	14:0_14:0_14:1	0.15,11/0.13,11	16:0_14:4_11:2	0.47,13/<0.01,9	16:0_18:1_6:0	0.46,15/0.11,12	16:1_16:1_18:1	<0.01,6/0.02,13	16:4_6:0_20:0	<0.01,14/<0.01,9
16:0_16:1	<0.01,1	14:0_14:0_16:0	0.03,12/0.09,14	16:0_16:0_16:0	0.15,13/0.34,15	16:0_18:2_10:0	0.30,15/0.21,16	16:1_16:1_18:2	0.69,15/0.07,12	17:0_18:1_16:0	0.32,15/0.25,16
16:0_18:1	0.01,2	14:0_18:2_11:1	0.07,15/<0.01,14	16:0_16:0_16:1	0.01,3/<0.01,2	16:0_18:2_10:3	0.01,12/<0.01,10	16:1_16:1_18:3	0.15,11/0.02,16	18:0_12:2_11:2	0.02,12/<0.01,2
16:1_18:2	0.03,12	14:1_11:4_18:0	0.13,10/<0.01,5	16:0_16:0_18:1	2.87,9/3.48,13	16:0_18:2_13:0	0.13,15/0.10,16	16:1_17:0_16:0	0.22,15/0.26,16	18:0_16:0_16:0	0.11,10/0.09,11
16:1_20:4	0.06,3	14:1_11:4_18:1	0.33,15/<0.01,16	16:0_16:0_18:2	2.05,6/2.91,8	16:0_18:2_18:1	1.23,8/<0.01,2	16:1_17:1_16:0	0.29,15/0.29,16	18:0_16:0_18:0	0.02,5/0.03,13
18:1_18:1	<0.01,3	15:0_10:2_13:0	0.01,13/0.01,6	16:0_16:1_13:0	0.05,9/0.08,16	16:0_18:3_11:1	0.02,12/<0.01,10	16:1_18:1_12:0	<0.01,2/<0.01,5	18:0_16:0_18:2	0.03,1/0.10,2
18:1_18:2	0.04,11	15:0_11:3_14:3	0.01,15/<0.01,9	16:0_16:1_14:0	1.04,14/2.01,14	16:0_18:3_12:0	0.12,14/0.17,16	16:1_18:1_17:1	0.22,14/0.08,16	18:0_16:0_20:4	0.01,15/0.01,14
18:1_20:5	0.01,4	15:0_16:0_16:1	0.13,15/0.20,16	16:0_16:1_16:0	2.43,15/2.87,11	16:0_18:3_18:3	0.11,13/<0.01,13	16:1_18:1_18:1	0.32,8/<0.01,1	18:0_18:0_16:0	0.01,4/<0.01,2
**TG**		**TG**		**TG**		**TG**		**TG**		**TG**	
**Identity**	**Abundance**	**Identity**	**Abundance**	**Identity**	**Abundance**	**Identity**	**Abundance**	**Identity**	**Abundance**	**Identity**	**Abundance**
18:0_18:0_18:1	0.94,15/0.76,16	18:2_12:2_12:2	0.01,12/<0.01,8	26:0_16:0_18:1	0.05,15/0.17,16	4:0_16:1_16:1	0.07,12/0.14,13	16:0_8:0_16:1	<0.01,2	18:3_12:2_11:3	0.01,11
18:0_18:0_18:2	1.17,15/0.63,15	18:2_17:1_16:1	0.11,15/<0.01,14	26:0_16:0_24:1	<0.01,12/0.03,16	4:0_16:1_18:3	0.02,5/0.06,10	16:0_11:2_21:0	<0.01,2	18:3_13:0_12:4	<0.01,1
18:0_18:1_16:0	0.96,11/0.49,10	18:2_17:1_18:1	0.10,15/<0.01,16	26:1_16:0_18:1	0.04,15/0.21,16	4:0_18:1_16:0	0.30,1/0.01,2	16:0_11:4_14:3	<0.01,2	18:4_11:3_14:4	<0.01,13
18:0_18:3_20:1	0.04,15/<0.01,16	18:2_18:2_18:2	0.17,8/0.01,16	26:1_18:1_16:1	0.03,15/0.17,16	4:0_18:1_16:1	0.60,14/0.22,12	16:0_14:0_14:1	<0.01,1	20:5_11:3_12:2	0.02,14

18:1_11:1_16:0	0.19,8/0.03,16	18:3_18:2_18:2	0.06,13/<0.01,16	26:1_18:1_18:1	0.04,15/0.16,13	4:0_18:1_17:0	0.06,15/0.02,13	16:0_18:1_10:3	<0.01,1	21:1_12:2_12:4	<0.01,1
18:1_11:1_18:1	<0.01,6/0.01,7	18:3_18:3_18:2	0.02,15/<0.01,10	28:0_16:0_18:1	0.02,15/0.07,16	4:0_18:1_18:2	<0.01,2/<0.01,3	16:0_18:3_10:0	0.01,3	22:4_10:1_11:3	<0.01,5
18:1_11:2_14:2	0.11,15/<0.01,16	18:4_14:0_16:1	0.02,15/0.04,12	28:0_18:1_18:1	0.01,15/0.04,16	4:0_18:1_18:4	0.02,12/0.02,12	16:1_10:0_16:0	0.01,2	41:6#	<0.01,2
18:1_11:2_18:1	0.07,15/<0.01,5	18:4_16:0_16:1	0.01,7/0.03,11	28:1_18:1_16:0	0.03,15/0.11,16	4:0_18:2_16:1	0.10,14/<0.01,7	16:1_11:1_18:2	0.05,11	4:0_16:0_18:1	<0.01,1
18:1_12:2_11:3	0.15,9/<0.01,7	18:4_16:1_16:1	0.02,13/0.02,11	28:1_18:1_18:1	0.01,15/0.06,16	4:0_18:2_18:2	0.05,13/<0.01,16	16:1_12:0_16:0	<0.01,1	4:0_18:1_18:0	<0.01,1
18:1_12:2_12:2	0.01,7/0.01,16	18:4_6:0_16:0	0.02,3/<0.01,2	30:0_18:1_16:0	0.01,15/0.03,16	4:0_18:3_16:1	0.03,12/<0.01,16	16:1_12:0_16:1	<0.01,1	16:0_11:3_12:2	<0.01,5
18:1_17:1_16:0	0.42,15/0.23,16	19:0_12:2_18:2	<0.01,6/<0.01,3	4:0_11:1_21:0	<0.01,13/0.01,13	4:0_18:3_18:0	0.06,7/<0.01,9	16:1_14:1_16:1	<0.01,1	16:0_11:4_14:2	<0.01,2
18:1_18:1_17:0	0.17,15/0.09,16	19:0_18:1_16:0	0.24,15/0.16,16	4:0_11:1_23:0	0.01,9/0.02,6	4:0_18:3_18:3	0.01,6/<0.01,15	16:1_14:4_11:2	0.08,14	16:0_18:1_18:0	<0.01,1
18:1_18:1_17:1	0.14,15/0.04,15	19:0_19:0_14:4	0.59,14/0.16,15	4:0_14:0_16:0	0.01,15/0.06,16	6:0_12:1_18:3	0.01,11/0.01,6	16:1_18:1_16:0	<0.01,1	18:0_11:2_14:3	<0.01,1
18:1_18:1_18:1	0.95,15/0.67,16	20:0_10:2_11:4	0.01,12/<0.01,3	4:0_14:1_16:0	0.03,11/0.10,12	6:0_18:1_18:2	<0.01,14/<0.01,13	16:1_18:3_11:1	0.01,11	18:0_18:1_20:0	<0.01,1
18:1_18:1_20:0	0.25,15/0.19,16	20:0_11:3_14:2	0.03,14/<0.01,1	4:0_14:1_16:1	0.01,13/0.03,16	8:0_11:2_22:6	<0.01,5/<0.01,15	16:2_10:4_16:0	0.01,14	18:1_14:0_14:0	<0.01,1
18:1_18:1_6:0	0.04,12/0.01,4	20:0_14:3_11:3	0.01,10/<0.01,4	4:0_14:2_22:3	0.01,12/<0.01,16	9:0_18:1_16:0	0.04,12/0.02,16	16:2_11:4_16:1	0.01,6	18:1_18:1_16:0	0.19,4
18:1_18:2_6:0	0.04,15/<0.01,13	20:0_16:0_16:1	<0.01,14/<0.01,7	4:0_16:0_16:0	0.11,15/0.10,16	4:0_16:0_20:5	0.02,15	16:4_11:3_18:1	0.01,15	18:1_18:1_18:0	0.02,5
18:1_18:3_11:1	0.03,13/<0.01,7	20:0_16:0_18:1	0.02,3/0.010,2	4:0_16:0_18:0	0.03,14/<0.01,11	4:0_16:1_18:1	<0.01,7	17:1_14:4_11:2	0.01,14	18:1_18:1_18:2	<0.01,1
18:1_24:0_16:0	0.07,15/0.12,16	20:0_18:2_18:1	0.11,15/0.07,16	4:0_16:0_18:3	0.01,2/0.01,9	4:0_18:1_20:0	<0.01,2	18:1_11:3_14:4	0.01,7	18:2_17:1_16:0	<0.01,1
18:2_11:1_16:0	0.14,15/0.01,16	20:1_18:1_18:3	0.01,15/<0.01,14	4:0_16:0_22:5	0.03,8/<0.01,9	4:0_18:2_18:3	0.01,14	18:1_14:4_11:2	0.08,11	18:3_18:3_18:1	<0.01,1
18:2_11:1_18:0	0.14,15/0.01,15	20:5_11:2_12:2	0.05,14/<0.01,5	4:0_16:1_14:0	0.01,5/0.03,7	6:0_11:3_23:0	<0.01,1	18:2_11:4_14:3	0.01,15		
18:2_11:1_18:1	0.02,8/<0.01,15	25:0_6:0_11:1	<0.01,5/<0.01,3	4:0_16:1_16:0	0.26,8/0.25,6	16:0_6:0_18:1	<0.01,4	18:2_14:2_11:4	0.02,8		

Table S5: Phospholipids and their percentage abundances and numbers of replicates in which they were found on both Days. The data for species found in both Days are shown in black, with the Day 1 and Day 19 abundance data before and after the slash, respectively, and those found in Day 1 only and Day 19 only are shown in blue and red, respectively.

CL		CL		PC		PC		PE		PE	
**Lipid identity**	**Abundance**	**Lipid identity**	**Abundance**	**Lipid identity**	**Abundance**	**Lipid identity**	**Abundance**	**Lipid identity**	**Abundance**	**Lipid identity**	**Abundance**
14:0_20:4_16:1_16:1	0.08,15/0.32,16	18:3_18:2_18:2_18:2	0.36,15/0.17,16	14:0_18:3	0.04,15/0.02,16	18:4_18:1	0.01,2/0.08,14	16:0_16:0	0.12,15/0.08,16	20:0_18:2	0.09,15/0.02,11
16:1_16:1_16:1_16:1	0.07,14/0.59,16	18:4_16:1_14:0_20:4	0.01,14/0.04,16	16:0_14:0	0.05,15/0.04,16	25:1_8:0	<0.01,3/0.02,8	16:0_16:1	1.61,15/2.34,16	16:0_22:6	0.01,2
18:1_16:1_16:1_16:1	0.06,14/0.22,16	18:4_16:1_16:1_16:1	0.01,13/0.06,16	16:0_16:0	0.39,15/0.24,15	33:8#	<0.01,5/<0.01,9	16:0_18:1	6.70,15/5.05,16	16:1_18:2	<0.01,1
18:1_16:1_16:1_18:1	0.04,15/0.07,16	18:4_16:1_16:1_18:1	<0.01,4/0.02,13	16:0_16:1	1.24,15/1.42,16	34:5#	0.01,8/<0.01,10	16:0_18:2	1.82,15/7.71,16	16:1_20:1	0.07,6
18:1_16:1_18:2_18:2	0.10,15/0.07,16	18:4_18:2_16:1_16:1	0.02,15/0.06,16	16:0_18:1	1.69,15/1.43,16	34:6#	<0.01,13/<0.01,6	16:0_18:3	2.42,15/1.74,16	18:1_21:1	2.44,7
18:2_14:1_22:6_18:2	0.04,13/0.04,16	18:4_18:2_16:1_18:2	0.02,8/0.06,16	16:0_18:3	1.00,15/0.48,16	6:0_22:1	0.01,15/0.01,16	16:1_16:1	0.57,15/2.37,16	18:4_20:5	<0.01,5
18:2_16:0_16:1_18:2	0.06,15/0.12,16	18:4_18:2_16:1_22:6	0.06,15/0.03,16	16:1_14:0	0.08,15/0.12,16	6:0_24:2	0.01,14/0.03,16	16:1_18:3	1.03,15/0.73,16	20:0_18:3	0.02,7
18:2_16:1_16:1_16:1	0.14,15/0.59,16	18:4_18:2_18:2_18:2	0.41,15/0.13,16	16:1_16:1	0.54,15/1.30,16	16:0_18:2	1.55,15	17:0_18:1	0.05,15/0.03,14	25:1_11:0	0.03,2
18:2_16:1_16:1_18:2	0.22,15/0.52,16	20:5_18:2_16:1_18:2	0.02,6/0.04,16	16:1_18:1	0.61,15/2.48,16	16:0_20:4	0.03,8	17:1_18:1	0.04,15/0.07,16	38:7#	0.05,7
18:2_16:1_18:2_18:2	0.26,15/0.32,16	22:6_14:1_16:1_18:2	0.03,15/0.05,16	16:1_18:3	0.56,15/0.32,16	33:4#	0.03,14	18:0_14:3	0.04,15/0.02,16		
18:2_18:2_18:2_18:2	0.19,15/0.12,16	70:11#	0.02,15/0.03,16	17:0_18:1	0.05,9/0.06,9			18:0_16:0	0.06,15/0.06,16		
18:3_16:1_14:0_20:4	0.10,15/0.16,16	72:12#	0.14,15/0.05,16	17:0_18:3	0.07,15/0.02,16			18:0_18:1	1.16,15/1.13,16		
18:3_16:1_14:0_22:6	0.15,15/0.07,16	72:13#	0.02,15/0.01,12	17:1_18:1	0.05,15/0.07,16			18:0_18:3	4.15,15/4.02,16		
18:3_16:1_16:1_20:4	0.32,15/0.20,16	74:12#	0.05,15/0.02,16	18:0_16:0	0.09,15/0.08,15			18:1_18:1	2.56,15/4.47,16		
18:3_16:1_18:2_16:1	0.22,15/0.43,16	74:14#	0.05,15/0.02,16	18:0_18:1	0.15,13/0.08,6			18:1_18:3	3.64,15/2.10,16		
18:3_18:2_14:0_22:6	0.31,15/0.09,16	74:15#	0.03,14/0.01,11	18:3_18:2	0.74,14/0.15,16			18:3_18:2	1.66,15/0.65,16		
18:3_18:2_14:1_22:6	0.04,15/0.03,16	18:2_18:2_16:1_18:1	<0.01,1	18:3_18:3	0.01,2/0.01,2			18:3_18:3	1.28,15/0.73,16		
18:3_18:2_16:1_18:2	0.39,15/0.35,16	18:3_18:1_18:2_18:2	<0.01,1	18:4_16:0	0.02,7/0.06,15			20:0_18:1	0.12,15/0.12,16		

PG		PI		PS		PS		LPC	
**Lipid identity**	**Abundance**	**Lipid identity**	**Abundance**	**Lipid identity**	**Abundance**	**Lipid identity**	**Abundance**	**Lipid identity**	**Abundance**
16:0_16:1	0.23,15/0.82,16	16:0_16:1	0.37,15/0.66,16	16:0_18:1	0.43,15/0.30,16	34:4#	0.04,7	12:0#	<0.01,11/0.01,16
16:0_18:1	0.22,15/0.57,16	16:0_18:1	0.72,15/0.74,16	16:1_20:1	0.02,3/0.01,1	38:5#	0.01,3	14:0#	0.01,15/0.02,16
16:0_18:2	0.99,15/1.14,16	16:0_18:2	1.14,15/0.94,16	18:0_18:1	0.44,15/0.33,16	38:6#	0.04,7	14:1#	<0.01,15/0.02,16

16:0_18:3	0.79,15/0.46,16	16:0_18:3	0.92,15/0.22,16	18:0_18:3	0.08,4/0.04,4	39:1#	0.35,7	16:0#	0.14,15/0.12,16
16:1_16:1	0.02,15/0.26,16	16:1_16:1	0.08,15/0.34,16	18:1_18:1	0.74,13/0.90,12	39:3#	0.16,7	16:1#	0.22,15/0.71,16
16:1_18:3	0.07,15/0.19,16	16:1_18:3	0.21,15/0.14,16	18:1_18:2	0.48,15/0.28,16	39:5#	0.03,7	16:2#	<0.01,14/<0.01,15
18:1_18:3	0.44,15/0.31,16	18:0_18:1	0.13,15/0.23,16	18:1_18:3	0.66,15/0.31,16			17:0#	0.01,15/0.01,16
18:3_18:3	0.08,14/0.06,16	18:0_18:2	0.39,15/0.54,16	18:3_18:2	0.18,15/0.10,16			17:1#	0.01,15/0.02,16
24:2_10:2	0.69,15/0.90,16	18:0_18:3	0.76,15/0.43,16	18:3_18:3	0.40,15/0.32,16			18:0#	0.02,15/0.03,16
		18:1_18:2	0.52,11/0.24,7	16:2_18:0	<0.01,1			18:1#	0.35,15/0.74,16
		18:1_18:3	1.54,15/0.57,16	18:1_20:2	0.05,7			18:2#	0.27,15/0.18,16
		18:3_18:3	0.44,15/0.23,16	18:1_20:4	0.07,7			18:3#	0.18,15/0.10,16
		16:1_18:3	<0.01,1	18:3_20:5	0.02,7			18:4#	<0.01,13/<0.01,7
		18:3_18:2	0.07,7	19:0_18:1	0.73,7			20:1#	<0.01,15/0.01,16
		30:1#	0.01,7	33:0#	0.04,7			20:4#	<0.01,0/<0.01,1

Table S6: Ether lipids and their percentage abundances and numbers of replicates in which they were found on both Days. The data for species found in both Days are shown in black, with the Day 1 and Day 19 abundance data before and after the slash, respectively, and those found in Day 1 only and Day 19 only are shown in blue and red, respectively.

DGe		TGe		TGe		PCe	
**Lipid identity**	**Abundance**	**Lipid identity**	**Abundance**	**Lipid identity**	**Abundance**	**Lipid identity**	**Abundance**
22:3e	0.34,15/0.10,7	18:0e_16:0_16:1	<0.01,2/<0.01,4	32:1e	0.13,14/0.09,16	20:0e_14:1	0.01,15/0.03,16
32:3e	0.12,15/0.09,7	18:0e_16:1_18:1	<0.01,1/<0.01,3	34:2e	0.16,14/0.04,8		
34:3e	0.28,15/0.09,7	18:3e_17:0_19:0	<0.01,4/<0.01,4	14:1e_11:3_18:2	<0.01,7		
34:4e	0.27,15/0.09,6	20:0e_16:0_18:1	<0.01,8/<0.01,4	18:0e_16:0_18:1	<0.01,1		
34:5e	0.19,15/0.04,7	20:0e_16:1_18:1	<0.01,2/<0.01,2				
36:3e	0.11,15/0.04,7	20:0e_18:1_18:1	<0.01,13/<0.01,4				

PEe		PEe		PEp		PSe	
**Lipid identity**	**Abundance**	**Lipid identity**	**Abundance**	**Lipid identity**	**Abundance**	**Lipid identity**	**Abundance**
12:0e_24:2	0.84,15/0.42,16	37:3e	<0.01,7/0.01,16	10:0p_24:0	0.17,15/0.30,16	38:5e	0.14,15/0.04,7
16:1e_18:1	0.11,15/0.23,16	38:2e	<0.01,8/0.07,16	12:0p_24:0	0.54,15/0.67,16		
18:0e_18:2	<0.01,6/0.04,9	38:3e	0.01,8/<0.01,1	12:0p_24:2	1.10,15/0.80,16		
20:0e_18:1	0.10,15/0.15,16	42:3e	<0.01,4/<0.01,2	18:0p_16:1	<0.01,4/<0.01,1		
20:0e_18:2	0.27,15/0.12,16	16:0e_22:6	0.14,14	18:0p_18:1	0.31,15/0.30,16		
20:0e_18:3	0.25,15/0.18,16	18:2e_19:0	<0.01,6	18:0p_18:3	0.20,15/0.07,16		
36:0e	0.01,14/0.02,15			18:0p_18:2	0.08,8		

Table S7: Comparison of lipid compositions in *B. tryoni* with those reported for *D. melanogaster* and the lepidopterans *B. mori* and *Samia cynthia*. The dash indicates the class was not reported in the study in question. All compositions are expressed as relative concentrations (i.e., in percentages of all lipids measured, by weight or, in the case of Carvalho *et al*. [51], Guan *et al*. [52] and Ko *et al*. [54], by mols). *

Class	Present study		Carvalho *et al.* [51]		Guan *et al.* [52]		Colinet *et al.* [53]	Ko *et al.* [54]	Hofbauer *et al.* [43]	Zhu *et al.* [61]	Ravinder *et al.* [60]
	B. tryoni		*D. melanogaster*							*B. mori*	*S. cynthia*
	Adult males		Larvae	Pupae	Adult males		Adult females	Virgin females	Adult males	Larvae	Pupae
	Day 1	Day 19			Day 1	Day 20	Day 6		Day 7		
**Neutral lipids**											
**DG**	0.4±0.05	0.4±0.04	—	—	—	—	—	*	2.9	—	—
**TG**	36.1±2.7	29.7±2.1	—	—	—	—	—	*	54.9	—	—
**Phospholipids**											
**CL**	4.1±0.2	5.1±0.2	—	—	~0.4	~0.9	—	—	—	—	9.05
**PC**	9±0.4	8.6±0.2	17	8	~15.0	~14.3	50.1	18.9	14.1	52.7	19.36
**PE**	29.1±1.4	36±1.1	33	32	~64.2	~62.1	38.7	60.4	20	21.1	64.97
**PG**	3.5±0.2	4.7±0.1	—	—	~2.3	~2.6	3.8	0.9	—	0.4	—
**PI**	7.2±0.3	5.4±0.2	18	16	~6.9	~7.3	4.6	9	6.9	1.8	6.37
**PS**	3.4±0.2	4.1±0.4	22	38	~2.9	~2.7	1.7	2.8	1.1	0.9	—
**PA**	—	—	—	—	~0.29	~0.27	—	0.5	—	0.3	—
**Lyso PC**	1.3±0.1	2±0.2	—	—	—	—	0.5	3.3	—	—	—
**Lyso PE**	—	—	—	—	—	—	0.5	4.2	—	—	—
**Lyso PG**	—	—	—	—	—	—	0.02	—	—	—	—
**Lyso PI**	—	—	—	—	—	—	0.01	—	—	—	—

**Sphingolipids**	–	–	–	–	~5.4	~5.9	–	–	–	11.6	–
**Ether neutral lipids**											
**DGe**	1.3±0.1	0.5±0.1	–	–	–	–	–	–	–	–	–
**TGe**	0.3±0.03	0.2±0.02	–	–	–	–	–	–	–	–	–
**Ether phospholipids**											
**PCe**	0.01±0	0.03±0	–	–	–	–	–	–	–	–	–
**PEe**	1.7±0.1	1.2±0.1	–	–	–	–	–	–	–	–	–
**PEp**	2.4±0.1	2.2±0.1	–	–	–	–	–	–	–	–	–
**PSe**	0.1±0.01	0.04±0.01	–	–	–	–	–	–	–	–	–

*As per **Table S3** the data of Scheitz *et al*. [30] were not presented in this table. Early studies based on quantification of thin layer chromatography spots in *D. melanogaster* respectively were also not considered, nor were some *Drosophila* studies that did use mass spectrometry but only focussed on a few specific lipids or tissues. However, it is noted that one such study, Overgaard *et al*. [42], reported PE/PC ratios as 1.1 and 1.4, depending on temperature.

Table S8: **Comparison of mean carbon chain lengths and double bond numbers (before and after the dash respectively) of acyl chains in ester lipid classes of *B. tryoni* males with those reported for *D. melanogaster.***

Class	Mean number of carbons/double bonds					
	Present study		Carvalho *et al.* [51]	Colinet *et al.* [53]	Ko *et al.* [54]	Hofbauer *et al.* [43]
	B. tryoni males		*D. melanogaster* males		*D. melanogaster* females	*D. melanogaster* males
	Day 1	Day 19	Day 9	Day 6	Virgin	Day 7
**Neutral lipids**						
DG	16.8/1.2	17.1/1.5	15.2/0.5	–	*	15.5/0.6
TG	16.3/0.8	16.5/0.6	15.3/0.5	–	*	15.3/0.5
**Phospholipids**						
PC	16.8/1.0	16.6/0.9	16.5/0.9	16.6/0.9	16.9/1.1	17.0/1.2
PE	17.4/1.3	17.4/1.1	17.0/1.0	16.8/0.9	17.3/1.2	17.4/1.3
PG	17.1/1.4	16.8/1.2	16.5/0.7	16.7/0.9	16.5/0.9	-
PI	17.5/1.4	17.2/1.2	16.8/1.0	16.9/1.1	16.9/1.0	17.4/1.3
PS	17.9/1.5	18.1/1.2	17.7/1.0	18.0/1.4	17.5/1.3	17.9/1.1
LPC	17.3/1.4	17.1/1.1	–	16.6/0.9	17.2/1.2	–

Table S9: Common chains and their percentage abundances in ester lipids in Day 1 and Day 19 males. See text and **Figure 3** for the definition of common chains.

	**10:0**	**10:2**	**11:3**	**12:0**	**14:1**	**16:0**	**16:1**	**17:0**	**18:0**	**18:1**	**18:2**	**18:3**	**19:0**	**20:0**	**20:4**	**24:0**	**24:2**
**Neutral lipids**																	
**Day 1 DG**	0	0	0	0	0	8.8	38.0	0	1.8	13.6	10.9	10.3	0	9.2	3.6	0	0
**Day 19 DG**	0	0	0	0	0	7.1	24.6	0	3.8	17.7	16.0	9.7	0	6.6	9.5	0	0
**Day 1 TG**	0.5	0.02	0.5	1.2	0.7	27.4	14.6	0.8	5.5	21.7	9.0	1.5	2.9	0.4	0.05	0.1	0
**Day 19 TG**	0.8	0.01	0.01	1.4	0.3	38.1	13.6	0.8	4.1	22.4	7.8	0.4	0.7	0.3	0.02	0.3	0
**Phospholipids**																	
**Day 1 CL**	0	0	0	0	0.8	0.4	26.7	0	0	1.7	42.7	12.4	0	0	3.4	0	0
**Day 19 CL**	0	0	0	0	0.6	0.6	48.1	0	0	2.3	30.2	7.5	0	0	3.6	0	0
**Day 1 PC**	0	0	0	0	0	36.0	20.4	0.6	1.3	14.6	12.6	12.7	0	0	0	0	0.1
**Day 19 PC**	0	0	0	0	0	23.6	40.7	0.4	1.0	24.5	0.9	5.8	0	0	0.2	0	0.1
**Day 1 PE**	0	0	0	0	0	22.4	6.8	0.1	9.4	28.9	6.2	25.7	0	0.4	0	0	0
**Day 19 PE**	0	0	0	0	0	23.8	10.9	0.0	7.3	27.6	11.7	15.0	0	0.2	0	0	0
**Day 1 PG**	0	10.3	0	0	0	31.4	5.1	0	0	9.3	14.0	19.6	0	0	0	0	10.3
**Day 19 PG**	0	9.5	0	0	0	31.8	16.4	0	0	9.5	12.1	11.3	0	0	0	0	9.5
**Day 1 PI**	0	0	0	0	0	22.0	5.3	0	8.6	20.4	14.6	29.0	0	0	0	0	0
**Day 19 PI**	0	0	0	0	0	24.0	13.8	0	11.1	16.7	16.7	17.8	0	0	0	0	0
**Day 1 PS**	0	0	0	0	0	6.2	0.2	0	7.5	50.3	10.4	25.2	0	0	0	0	0
**Day 19 PS**	0	0	0	0	0	4.5	0.2	0	5.6	56.1	5.7	16.6	9.3	0	1.0	0	0
**Day 1 LPC**	0	0	0	0.1	0.2	11.2	19.2	0.5	1.9	29.6	21.5	13.7	0	0	0	0	0
**Day 19 LPC**	0	0	0	0.3	0.8	6.0	36.0	0.7	1.3	37.6	9.4	5.5	0	0	0.1	0	0

Table S10: Common acyl and akyl/alkenyl chains and their percentage abundances in ether lipids in Day 1 and Day 19 males. See text and **Figure 3** for the definition of common chains.

	**10:0**	**11:3**	**12:0**	**14:1**	**16:0**	**16:1**	**17:0**	**18:0**	**18:1**	**18:2**	**18:3**	**19:0**	**20:0**	**22:6**	**24:0**	**24:2**
**alkyl neutral lipids**																
**Day 1 TGe_acyl**	0	8.3	0	0	12.2	8.0	4.1	0	48.7	8.3	0	4.1	0	0	0	0
**Day 1 TGe_alkyl**	0	0	0	16.6	0	0	0	10.2	0	0	8.2	0	58.7	0	0	0
**Day 19 TGe acyl**	0	0	0	0	13.3	20.1	10.3	0	33.5	0	0	10.3	0	0	0	0
**Day 19 TGe_alkyl**	0	0	0	0	0	0	0	29.9	0	0	20.7	0	37.0	0	0	0
**alkyl phospholipids**																
**Day 1 PCe_acyl**	0	0	0	100	0	0	0	0	0	0	0	0	0	0	0	0
**Day 1 PCe_alkyl**	0	0	0	0	0	0	0	0	0	0	0	0	100	0	0	0
**Day 19 PCe_acyl**	0	0	0	100	0	0	0	0	0	0	0	0	0	0	0	0
**Day 19 PCe_alkyl**	0	0	0	0	0	0	0	0	0	0	0	0	100	0	0	0
**Day 1 PEe_acyl**	0	0	0	0	0	0	0	0	11.9	16.0	14.6	0.03	0	7.7	0	49.7
**Day 1 PEe_alkyl**	0	0	49.7	0	7.7	6.3	0	0.1	0	0.03	0	0	36.1	0	0	0
**Day 19 PEe_acyl**	0	0	0	0	0	0	0	0	33.8	13.5	16.6	0	0	0	0	36.2
**Day 19 PEe_alkyl**	0	0	36.2	0	0	20.6	0	2.9	0	0	0	0	40.3	0	0	0
**Day 1 PEp _acyl**	0	0	0	0	0	0.10	0	0	12.8	3.8	8.0	0	0	0	30.1	45.3
**Day 1 PEp_alkenyl**	7.2	0	68.1	0	0	0	0	24.7	0	0	0	0	0	0	0	0
**Day_19 PEp_acyl**	0	0	0	0	0	0.002	0	0	13.9	0	3.5	0	0	0	45.2	37.5
**Day 19 PEp_alkenyl**	14.0	0	68.6	0	0	0	0	17.3	0	0	0	0	0	0	0	0

Table S11: Comparison of the relative abundances of different acyl chains in ester neutral lipids of *B. tryoni* with their abundances in two other tephritids, *B. oleae* and *C. capitata*, plus *D. melanogaster*, the mosquito *Aedes cinerius* and the lepidopteran *S. cynthia*. The numbers in each cell are, in order: whole body extracts of Day1 and Day 19 adult males for *B. tryoni*; whole body extracts of larvae, young adults and old adults for *B. oleae*; whole body extracts of larvae, young adults and old adults for *C. capitata*; lysosomal fractions of whole body extracts of larvae (126 hr) for *D. melanogaster*; whole body extracts of larvae and adults for *Ae. cinerius*; and whole body extracts of larvae fed castor or tapioca leaves for *S. cynthia*. The data for *B. oleae*, *C. capitata* and *Ae. cinerius* are also for TGs only. Dashes represent not detected or, in the case of *C. capitata*, either not detected or <0.1%. tr = trace.

Acyl chains	Present Study	Madariaga *et al.* [94]	Pagani *et al.*, [95]	Jones *et al.* [93]	Suschick *et al.* [59]	Ravinder *et al.* [60]
	B. tryoni	***B. oleae***	***C. capitata***	***D. melanogaster***	***Ae. cinerius***	***S. cynthia***
	wt %	wt %	wt %	mol %	mg/g of wet weight	wt %
**10:0#**	0.4, 0.8	–	0.4, 3.6, 2.8	–	–	–
**11:1#**	0.9, <0.2	–	–	–	–	–
**11:2#**	1, < 0.2%	–	–	–	–	–
**11:3#**	0.5, < 0.2%	–	–	–	–	–
**12:0#**	1, 1	0.2, 0.3, 0.3	2.2, 0.4, –	–	0.5, 0.3	–
**14:0#**	1, 3	1.2, 1.0, 2.4	–	22.4	1.7, 2.2	0.4, 0.3
**14:1#**	0.7, 0.3	0.3, 0.4, 0.4	0.9, 0.5, 0.5	–	0.3, 0.4	–
**14:4#**	1, 0.2%	–	–	–	–	–
**16:0#**	27, 38	24.8, 23.0, 31.2	39.2, 36.6, 39.7	20.9	18.3, 24.3	28.8, 24.9
**16:1#**	15, 14	4.5, 5.7, 21.6	28.5, 31.3, 21.1	23.1	12.7, 15.1	1.9, 1.1
**17:0#**	0.8, 0.8	–	–	–	–	–
**18:0#**	5, 4	3.5, 3.0, 2.6	3.4, 3.4, 2.5	0.7	3.7, 3.2	3.7, 4.5
**18:1#**	21, 22	50.5, 49.6, 33.3	16.4, 16.0, 20	19	11.4, 18.8	19.1, 13.2
**18:2#**	9, 8	11.5, 11.0, 4.5	3.6, 2.8, 8.6	6.8	4.1, 4.8	5.6, 4.3
**18:3#**	2, 0.5	0.4, 0.3, tr	–	1.3	6.5, 4.9	40.3, 51.5
**19:0#**	3, 0.7	–	–	–	–	–
**20:0#**	0.5, 0.4	–	–	–	–	0.1, 0.2
**24:0#**	< 0.2%, 0.3	–	–	–	–	–

Table S12: Comparison of the relative abundances of different acyl chains in ester phospholipids of *B. tryoni* with their abundances in two other tephritids, *B. oleae* and *C. capitata*, plus *D. melanogaster*, the mosquito *Aedes cinerius* and the lepidopteran *S. cynthia*. The numbers in each cell are, in order: whole body extracts of Day1 and Day 19 adult males for *B. tryoni*; whole body extracts of larvae, young adults and old adults for *B. oleae*; whole body extracts of larvae, young adults and old adults for *C. capitata*; lysosomal fractions of whole-body extracts of larvae (126 hr), and prepupae for *D. melanogaster*; whole body extracts of larvae and adults for *Ae. cinerius*; and whole body extracts of larvae fed castor or tapioca leaves for *S. cynthia*. The data for *B. oleae*, *C. capitata* and *Ae. cinerius* are also for TGs only. Dashes represent not detected or, in the case of *C. capitata*, either not detected or <0.1%. tr = trace.

**Acyl chains**	**Present Study**	**Madariaga *et al.* [94]**	**Pagani *et al*. [95]**	**Jones *et al*. [93]**	**Suschick *et al*. [59]**	**Ravinder *et al*. [60]**
	B. tryoni	*B. oleae*	*C. capitata*	*D. melanogaster*	*Ae. cinerius*	*S. racini*
	wt %	wt %	wt %	mol %	mg/g of wet weight	wt %
**12:0#**	< 0.2, < 0.2	tr, 0.2, tr	–	–	–	–
**14:0#**	0.7, 0.6	0.5, 0.8, 0.3	0.6, 0.3, 0.2	–	0.5, 0.3	0.3, 0.3
**14:1#**	< 0.2, < 0.2	tr, tr, tr	–	–	–	–
**16:0#**	21, 20	23.2, 20.0, 13.0	19.9, 11, 24	13.2, 15.6	7.9, 13.1	17.8, 18.1
**16:1#**	11, 20	4.2, 6.4, 16.8	21.5, 21.6, 13.5	7.5,6.7	10.3, 7.5	0.6, 0.6
**17:0#**	0.2, < 0.2	–	–	–	–	–
**18:0#**	6, 5	4.6, 3.0, 2.6	4.2, 4, 3	3.7, 3.4	3.0, 4.0	15.3, 15.3
**18:1#**	22, 23	39.0, 29.4, 31.0	27.5, 32.5, 25.7	27.2, 27.3	18.9, 16.4	20.7, 15.8
**18:2#**	14, 13	23.0, 28.5, 27.7	22.2, 23.4, 26.7	32.2, 29.1	8.1, 9.9	12.2, 8.4
**18:3#**	22, 13	3.0, 9.9, 6.0	1.9, 5.2, 5.4	3.9, 5.2	13.7, 13.4	30.5, 38.6
**19:0#**	–, 0.5	–	–	–	–	–
**20:0#**	0.2, < 0.2	–	1.2, 1.7, 1.3	–	–	1.6, 1.7
**21:1#**	0,2	–	–	–	–	–
**24:2#**	0.6, 0.7	–	–	–	–	–

Table S13: Differences between observed and expected frequencies of combinations of acyl chains in the nine joint chain length/ double bond categories in each ester lipid class. Differences in the percentages are first expressed as ratios (± SE) of the percentages of observed vs expected, with the absolute difference given in brackets. Category abbreviations are as defined in the text. * Bonferroni-corrected P < 0.05; ** Bonferroni-corrected P < 0.01; *** Bonferroni-corrected P < 0.001.

Class	Category	Day 1	Day 19
		**Neutral lipids**	
DG	S0_S0	0.00000 ± 0.00000*** (-0.13313)	0.00000 ± 0.00000*** (-0.17581)
DG	S0_M0	0.00000 ± 0.00000*** (-0.77865)	0.00000 ± 0.00000*** (-0.91039)
DG	S0_M1	0.00000 ± 0.00000*** (-3.76726)	0.00000 ± 0.00000*** (-3.54261)
DG	S0_MX	0.00000 ± 0.00000*** (-1.54579)	0.00000 ± 0.00000*** (-2.15498)
DG	S0_L0	0.00000 ± 0.00000*** (-0.67328)	0.00000 ± 0.00000*** (-0.54928)
DG	S0_LX	27.40658 ± 10.83693 (7.03125)	9.56093 ± 1.29507*** (7.50889)
DG	M0_M0	0.00000 ± 0.00000*** (-1.13849)	0.00000 ± 0.00000*** (-1.17855)
DG	M0_M1	0.07728 ± 0.07335*** (-10.16518)	0.73610 ± 0.28253 (-2.42051)
DG	M0_MX	4.53254 ± 1.51406 (15.96832)	2.68136 ± 0.81380 (9.3811)
DG	M0_L0	0.00000 ± 0.00000*** (-1.96887)	0.00000 ± 0.00000*** (-1.42215)
DG	M0_LX	0.00000 ± 0.00000*** (-0.77865)	0.00000 ± 0.00000*** (-2.27093)
DG	M1_M1	1.26611 ± 0.19239 (7.09185)	0.63514 ± 0.17630 (-6.51122)
DG	M1_MX	0.75261 ± 0.22450 (-5.41051)	1.35576 ± 0.13987 (7.72396)
DG	M1_L0	1.93709 ± 0.27194** (8.92653)	2.36718 ± 0.27162*** (7.566)
DG	M1_LX	0.00000 ± 0.00000*** (-3.76726)	1.41820 ± 0.68250 (3.6956)
DG	MX_MX	0.60357 ± 0.52657 (-1.77878)	0.52993 ± 0.07201*** (-3.10412)
DG	MX_L0	0.00000 ± 0.00000*** (-3.90866)	0.00000 ± 0.00000*** (-3.36635)
DG	MX_LX	0.00000 ± 0.00000*** (-1.54579)	0.00000 ± 0.00000*** (-5.37548)
DG	L0_L0	0.00000 ± 0.00000*** (-0.85122)	0.00000 ± 0.00000*** (-0.42903)
DG	L0_LX	0.00000 ± 0.00000*** (-0.67328)	0.00000 ± 0.00000*** (-1.37016)
DG	LX_LX	0.00000 ± 0.00000*** (-0.13313)	0.00000 ± 0.00000*** (-1.09396)
TG	S0_S0_S0	0.00000 ± 0.00000*** (-0.02607)	0.00000 ± 0.00000*** (-0.05433)
TG	S0_S0_S1	25.35896 ± 7.40783~ (0.5068)	51.11366 ± 10.95296*** (0.42949)
TG	S0_S0_SX	0.35584 ± 0.07174*** (-0.03446)	5.47102 ± 2.14708 (0.02898)
TG	S0_S0_M0	0.33523 ± 0.05782*** (-0.27449)	0.70710 ± 0.07605** (-0.25138)
TG	S0_S0_M1	0.06415 ± 0.02590*** (-0.43292)	0.18790 ± 0.08924*** (-0.59814)
TG	S0_S0_MX	0.00000 ± 0.00000*** (-0.13858)	0.00000 ± 0.00000*** (-0.16673)
TG	S0_S0_L0	0.00000 ± 0.00000*** (-0.04676)	0.00000 ± 0.00000*** (-0.03846)
TG	S0_S0_L1	0.00000 ± 0.00000*** (-0.00311)	0.00000 ± 0.00000*** (-0.01809)
TG	S0_S0_LX	3.09861 ± 1.20512 (0.01651)	0.12531 ± 0.05325*** (-0.00129)
TG	S0_S1_S1	0.00000 ± 0.00000*** (-0.00553)	0.00000 ± 0.00000*** (-0.00045)
TG	S0_S1_SX	0.00000 ± 0.00000*** (-0.02846)	0.00000 ± 0.00000*** (-0.00068)
TG	S0_S1_M0	0.37732 ± 0.09797*** (-0.13678)	3.55114 ± 0.89447 (0.23024)
TG	S0_S1_M1	0.09415 ± 0.02033*** (-0.22294)	1.51035 ± 0.18658 (0.03953)
TG	S0_S1_MX	2.90979 ± 0.61087 (0.1408)	1.92737 ± 0.63173 (0.01626)
TG	S0_S1_L0	1.48571 ± 0.95861 (0.01208)	29.97119 ± 11.77950 (0.11717)
TG	S0_S1_L1	0.00000 ± 0.00000*** (-0.00166)	0.00000 ± 0.00000*** (-0.0019)
TG	S0_S1_LX	0.00000 ± 0.00000*** (-0.00419)	0.00000 ± 0.00000*** (-0.00016)
TG	S0_SX_SX	2.15313 ± 0.37497 (0.04218)	5.69543 ± 1.38920~ (0.00121)

TG	S0_SX_M0	0.16197 ± 0.04822*** (-0.47327)	1.25104 ± 0.68231 (0.01714)
TG	S0_SX_M1	0.00000 ± 0.00000*** (-0.63271)	0.00000 ± 0.00000*** (-0.05857)
TG	S0_SX_MX	0.01024 ± 0.01024*** (-0.1876)	0.00000 ± 0.00000*** (-0.01326)
TG	S0_SX_L0	0.00725 ± 0.00725*** (-0.06349)	0.00000 ± 0.00000*** (-0.00306)
TG	S0_SX_L1	0.00000 ± 0.00000*** (-0.00426)	0.00000 ± 0.00000*** (-0.00144)
TG	S0_SX_LX	12.87229 ± 2.23090*** (0.12776)	181.75578 ± 20.14453*** (0.02126)
TG	S0_M0_M0	0.20565 ± 0.02664*** (-1.73148)	0.24346 ± 0.02549*** (-3.41856)
TG	S0_M0_M1	1.36755 ± 0.23209 (1.79519)	1.48455 ± 0.13410* (3.75808)
TG	S0_M0_MX	3.02463 ± 0.32374*** (2.9623)	2.95174 ± 0.49228** (3.42677)
TG	S0_M0_L0	0.00000 ± 0.00000*** (-0.49369)	0.00000 ± 0.00000*** (-0.40498)
TG	S0_M0_L1	0.00000 ± 0.00000*** (-0.03289)	0.00000 ± 0.00000*** (-0.19048)
TG	S0_M0_LX	1.59825 ± 0.45288 (0.0497)	0.09504 ± 0.02612*** (-0.01409)
TG	S0_M1_M1	1.40469 ± 0.19574 (1.10723)	0.89922 ± 0.08590 (-0.3354)
TG	S0_M1_MX	0.37850 ± 0.04704*** (-1.01878)	0.29608 ± 0.03107*** (-1.06065)
TG	S0_M1_L0	0.02130 ± 0.01460*** (-0.54133)	0.00000 ± 0.00000*** (-0.34755)
TG	S0_M1_L1	0.00000 ± 0.00000*** (-0.03684)	0.00000 ± 0.00000*** (-0.16347)
TG	S0_M1_LX	0.00000 ± 0.00000*** (-0.09307)	0.00000 ± 0.00000*** (-0.01337)
TG	S0_MX_MX	3.43937 ± 0.45824*** (0.59894)	0.60787 ± 0.04505*** (-0.06688)
TG	S0_MX_L0	0.07358 ± 0.01644*** (-0.1535)	0.01118 ± 0.00305*** (-0.0778)
TG	S0_MX_L1	0.00000 ± 0.00000*** (-0.01104)	0.00000 ± 0.00000*** (-0.03701)
TG	S0_MX_LX	0.00000 ± 0.00000*** (-0.02788)	0.00000 ± 0.00000*** (-0.00303)
TG	S0_L0_L0	0.00000 ± 0.00000*** (-0.02795)	0.00000 ± 0.00000*** (-0.00907)
TG	S0_L0_L1	0.00000 ± 0.00000*** (-0.00372)	0.00000 ± 0.00000*** (-0.00854)
TG	S0_L0_LX	0.00000 ± 0.00000*** (-0.00941)	0.00000 ± 0.00000*** (-7e-04)
TG	S0_L1_L1	0.00000 ± 0.00000*** (-0.00012)	0.00000 ± 0.00000*** (-0.00201)
TG	S0_L1_LX	0.00000 ± 0.00000*** (-0.00063)	0.00000 ± 0.00000*** (-0.00033)
TG	S0_LX_LX	0.00000 ± 0.00000*** (-0.00079)	0.00000 ± 0.00000*** (-1e-05)
TG	S1_S1_S1	0.00000 ± 0.00000*** (-0.00049)	0.00000 ± 0.00000*** (-1e-05)
TG	S1_S1_SX	0.00000 ± 0.00000*** (-0.00379)	0.00000 ± 0.00000*** (-2e-05)
TG	S1_S1_M0	0.00000 ± 0.00000*** (-0.02922)	0.00000 ± 0.00000*** (-0.00237)
TG	S1_S1_M1	0.00000 ± 0.00000*** (-0.03274)	0.00000 ± 0.00000*** (-0.00204)
TG	S1_S1_MX	0.00000 ± 0.00000*** (-0.00981)	0.00000 ± 0.00000*** (-0.00046)
TG	S1_S1_L0	0.00000 ± 0.00000*** (-0.00331)	0.00000 ± 0.00000*** (-0.00011)
TG	S1_S1_L1	0.00000 ± 0.00000*** (-0.00022)	0.00000 ± 0.00000*** (-5e-05)
TG	S1_S1_LX	0.00000 ± 0.00000*** (-0.00056)	0.00000 ± 0.00000*** (0)
TG	S1_SX_SX	0.00000 ± 0.00000*** (-0.00973)	0.00000 ± 0.00000*** (-1e-05)
TG	S1_SX_M0	3.28295 ± 0.88670 (0.34297)	0.69191 ± 0.39223 (-0.00111)
TG	S1_SX_M1	6.29731 ± 0.92097*** (0.8916)	2.01995 ± 0.35246 (0.00314)
TG	S1_SX_MX	0.00000 ± 0.00000*** (-0.05042)	0.00000 ± 0.00000*** (-7e-04)
TG	S1_SX_L0	0.00000 ± 0.00000*** (-0.01701)	0.00000 ± 0.00000*** (-0.00016)
TG	S1_SX_L1	0.00000 ± 0.00000*** (-0.00113)	0.00000 ± 0.00000*** (-8e-05)
TG	S1_SX_LX	0.55796 ± 0.22582 (-0.00126)	0.00000 ± 0.00000*** (-1e-05)
TG	S1_M0_M0	0.00000 ± 0.00000*** (-0.57985)	0.00000 ± 0.00000*** (-0.23759)
TG	S1_M0_M1	0.87067 ± 0.15583 (-0.16804)	0.39448 ± 0.03716*** (-0.24692)
TG	S1_M0_MX	2.93782 ± 0.84015 (0.75423)	0.50734 ± 0.09653*** (-0.04549)
TG	S1_M0_L0	0.00000 ± 0.00000*** (-0.13133)	0.00000 ± 0.00000*** (-0.02129)

TG	S1_M0_L1	0.00000 ± 0.00000*** (-0.00875)	0.00000 ± 0.00000*** (-0.01002)
TG	S1_M0_LX	0.00000 ± 0.00000*** (-0.0221)	0.00000 ± 0.00000*** (-0.00082)
TG	S1_M1_M1	0.00158 ± 0.00104*** (-0.72667)	0.17636 ± 0.05888*** (-0.14412)
TG	S1_M1_MX	0.87710 ± 0.27329 (-0.05359)	0.11779 ± 0.02222*** (-0.06989)
TG	S1_M1_L0	0.00000 ± 0.00000*** (-0.14714)	0.00000 ± 0.00000*** (-0.01827)
TG	S1_M1_L1	0.00000 ± 0.00000*** (-0.0098)	0.00000 ± 0.00000*** (-0.0086)
TG	S1_M1_LX	0.00000 ± 0.00000*** (-0.02476)	0.00000 ± 0.00000*** (-7e-04)
TG	S1_MX_MX	0.00000 ± 0.00000*** (-0.06532)	0.00000 ± 0.00000*** (-0.00897)
TG	S1_MX_L0	0.00000 ± 0.00000*** (-0.04408)	0.00000 ± 0.00000*** (-0.00414)
TG	S1_MX_L1	0.00000 ± 0.00000*** (-0.00294)	0.00000 ± 0.00000*** (-0.00195)
TG	S1_MX_LX	0.00000 ± 0.00000*** (-0.00742)	0.00000 ± 0.00000*** (-0.00016)
TG	S1_L0_L0	0.00000 ± 0.00000*** (-0.00744)	0.00000 ± 0.00000*** (-0.00048)
TG	S1_L0_L1	0.00000 ± 0.00000*** (-0.00099)	0.00000 ± 0.00000*** (-0.00045)
TG	S1_L0_LX	0.00000 ± 0.00000*** (-0.0025)	0.00000 ± 0.00000*** (-4e-05)
TG	S1_L1_L1	0.00000 ± 0.00000*** (-3e-05)	0.00000 ± 0.00000*** (-0.00011)
TG	S1_L1_LX	0.00000 ± 0.00000*** (-0.00017)	0.00000 ± 0.00000*** (-2e-05)
TG	S1_LX_LX	0.00000 ± 0.00000*** (-0.00021)	0.00000 ± 0.00000*** (0)
TG	SX_SX_SX	0.00000 ± 0.00000*** (-0.00834)	0.00000 ± 0.00000*** (0)
TG	SX_SX_M0	10.87781 ± 2.37592** (1.90749)	61.01902 ± 9.27497*** (0.08144)
TG	SX_SX_M1	8.13766 ± 1.18524*** (1.54423)	31.20618 ± 2.92511*** (0.03518)
TG	SX_SX_MX	3.17383 ± 0.68659 (0.14089)	1.76037 ± 0.53648 (2e-04)
TG	SX_SX_L0	6.16549 ± 1.36648* (0.11296)	81.36369 ± 38.61540 (0.00489)
TG	SX_SX_L1	0.11564 ± 0.11564*** (-0.00129)	0.00000 ± 0.00000*** (-3e-05)
TG	SX_SX_LX	63.75591 ± 18.00854* (0.23093)	72.80081 ± 37.30531 (0.00017)
TG	SX_M0_M0	0.00000 ± 0.00000*** (-1.49068)	0.00000 ± 0.00000*** (-0.17968)
TG	SX_M0_M1	0.03661 ± 0.00486*** (-3.21789)	0.03340 ± 0.01120*** (-0.2981)
TG	SX_M0_MX	0.05220 ± 0.01289*** (-0.94838)	0.00656 ± 0.00200*** (-0.06936)
TG	SX_M0_L0	0.00019 ± 0.00016*** (-0.33756)	0.00000 ± 0.00000*** (-0.0161)
TG	SX_M0_L1	0.00000 ± 0.00000*** (-0.02249)	0.00000 ± 0.00000*** (-0.00757)
TG	SX_M0_LX	0.00000 ± 0.00000*** (-0.05681)	0.00000 ± 0.00000*** (-0.00062)
TG	SX_M1_M1	0.12457 ± 0.04227*** (-1.638)	0.00910 ± 0.00459*** (-0.13114)
TG	SX_M1_MX	0.05538 ± 0.01101*** (-1.05896)	0.00011 ± 0.00011*** (-0.05991)
TG	SX_M1_L0	0.00000 ± 0.00000*** (-0.37826)	0.00000 ± 0.00000*** (-0.01382)
TG	SX_M1_L1	0.00000 ± 0.00000*** (-0.0252)	0.00000 ± 0.00000*** (-0.0065)
TG	SX_M1_LX	0.00000 ± 0.00000*** (-0.06365)	0.00000 ± 0.00000*** (-0.00053)
TG	SX_MX_MX	0.00000 ± 0.00000*** (-0.16791)	0.00000 ± 0.00000*** (-0.00678)
TG	SX_MX_L0	0.01058 ± 0.00524*** (-0.11211)	0.55482 ± 0.36132 (-0.00139)
TG	SX_MX_L1	0.00000 ± 0.00000*** (-0.00755)	0.00000 ± 0.00000*** (-0.00147)
TG	SX_MX_LX	0.00000 ± 0.00000*** (-0.01907)	0.00000 ± 0.00000*** (-0.00012)
TG	SX_L0_L0	94.09978 ± 30.62882 (1.77979)	1542.08948 ±149.54094*** (0.55604)
TG	SX_L0_L1	0.00000 ± 0.00000*** (-0.00255)	0.00000 ± 0.00000*** (-0.00034)
TG	SX_L0_LX	0.00000 ± 0.00000*** (-0.00643)	0.00000 ± 0.00000*** (-3e-05)
TG	SX_L1_L1	0.00000 ± 0.00000*** (-8e-05)	0.00000 ± 0.00000*** (-8e-05)
TG	SX_L1_LX	0.00000 ± 0.00000*** (-0.00043)	0.00000 ± 0.00000*** (-1e-05)
TG	SX_LX_LX	0.00000 ± 0.00000*** (-0.00054)	0.00000 ± 0.00000*** (0)
TG	M0_M0_M0	0.18586 ± 0.03258*** (-3.12281)	0.20943 ± 0.02995*** (-6.26967)

TG	M0_M0_M1	1.63672 ± 0.17333* (8.20864)	1.31265 ± 0.17599 (6.38359)
TG	M0_M0_MX	2.25080 ± 0.58577 (4.83066)	2.35957 ± 0.59377 (6.28414)
TG	M0_M0_L0	0.00000 ± 0.00000*** (-1.30313)	0.00000 ± 0.00000*** (-1.06613)
TG	M0_M0_L1	0.00000 ± 0.00000*** (-0.08681)	0.00000 ± 0.00000*** (-0.50146)
TG	M0_M0_LX	0.20429 ± 0.04722*** (-0.17448)	1.35026 ± 0.26536 (0.01436)
TG	M0_M1_M1	1.02274 ± 0.15723 (0.32852)	0.99233 ± 0.15198 (-0.13436)
TG	M0_M1_MX	0.34468 ± 0.14274*** (-5.67099)	0.85019 ± 0.07894 (-1.18847)
TG	M0_M1_L0	0.66895 ± 0.05142*** (-0.96663)	1.39721 ± 0.16581 (0.72686)
TG	M0_M1_L1	1.95384 ± 0.16362*** (0.18553)	1.40908 ± 0.21194 (0.35209)
TG	M0_M1_LX	0.00000 ± 0.00000*** (-0.49133)	0.00000 ± 0.00000*** (-0.07037)
TG	M0_MX_MX	0.23434 ± 0.05604*** (-0.99244)	0.00690 ± 0.00118*** (-0.89178)
TG	M0_MX_L0	0.00000 ± 0.00000*** (-0.87472)	0.00000 ± 0.00000*** (-0.41425)
TG	M0_MX_L1	1.97962 ± 0.36833 (0.05708)	0.08593 ± 0.01438*** (-0.1781)
TG	M0_MX_LX	9.18052 ± 1.93182** (1.20406)	9.01043 ± 0.89744*** (0.12761)
TG	M0_L0_L0	0.00000 ± 0.00000*** (-0.14757)	0.00000 ± 0.00000*** (-0.04777)
TG	M0_L0_L1	0.53203 ± 0.10101*** (-0.0092)	2.18535 ± 0.49674 (0.05327)
TG	M0_L0_LX	0.00000 ± 0.00000*** (-0.04966)	0.00000 ± 0.00000*** (-0.00367)
TG	M0_L1_L1	0.00000 ± 0.00000*** (-0.00065)	0.00000 ± 0.00000*** (-0.01057)
TG	M0_L1_LX	0.00000 ± 0.00000*** (-0.00331)	0.00000 ± 0.00000*** (-0.00173)
TG	M0_LX_LX	0.00000 ± 0.00000*** (-0.00418)	0.00000 ± 0.00000*** (-7e-05)
TG	M1_M1_M1	1.66050 ± 0.22903 (3.56277)	0.89573 ± 0.13387 (-0.52267)
TG	M1_M1_MX	0.62192 ± 0.08060*** (-1.83277)	0.12470 ± 0.02006*** (-2.9797)
TG	M1_M1_L0	0.56043 ± 0.03611*** (-0.719)	1.59814 ± 0.15573* (0.46966)
TG	M1_M1_L1	1.99973 ± 0.20877*** (0.10893)	3.75381 ± 0.68911** (1.01705)
TG	M1_M1_LX	0.00000 ± 0.00000*** (-0.27523)	0.00000 ± 0.00000*** (-0.0302)
TG	M1_MX_MX	1.23515 ± 0.30782 (0.34148)	0.22296 ± 0.02290*** (-0.59882)
TG	M1_MX_L0	0.35670 ± 0.04383*** (-0.63043)	0.72535 ± 0.08852 (-0.09765)
TG	M1_MX_L1	0.60236 ± 0.19065 (-0.02596)	0.01509 ± 0.00441*** (-0.16469)
TG	M1_MX_LX	0.00000 ± 0.00000*** (-0.1649)	0.00000 ± 0.00000*** (-0.01367)
TG	M1_L0_L0	0.00000 ± 0.00000*** (-0.16533)	0.00000 ± 0.00000*** (-0.041)
TG	M1_L0_L1	0.00000 ± 0.00000*** (-0.02203)	0.00000 ± 0.00000*** (-0.03857)
TG	M1_L0_LX	0.00000 ± 0.00000*** (-0.05564)	0.00000 ± 0.00000*** (-0.00315)
TG	M1_L1_L1	0.00000 ± 0.00000*** (-0.00073)	0.00000 ± 0.00000*** (-0.00907)
TG	M1_L1_LX	0.00000 ± 0.00000*** (-0.00371)	0.00000 ± 0.00000*** (-0.00148)
TG	M1_LX_LX	0.00000 ± 0.00000*** (-0.00468)	0.00000 ± 0.00000*** (-6e-05)
TG	MX_MX_MX	5.03169 ± 1.36258 (0.58463)	0.43141 ± 0.11778*** (-0.03306)
TG	MX_MX_L0	0.00000 ± 0.00000*** (-0.14679)	0.00000 ± 0.00000*** (-0.04024)
TG	MX_MX_L1	0.00000 ± 0.00000*** (-0.00978)	0.00000 ± 0.00000*** (-0.01893)
TG	MX_MX_LX	0.00000 ± 0.00000*** (-0.0247)	0.00000 ± 0.00000*** (-0.00155)
TG	MX_L0_L0	44.74349 ± 9.00328*** (2.16655)	19.74291 ± 2.55754*** (0.17396)
TG	MX_L0_L1	0.00000 ± 0.00000*** (-0.0066)	0.00000 ± 0.00000*** (-0.00873)
TG	MX_L0_LX	0.00000 ± 0.00000*** (-0.01667)	0.00000 ± 0.00000*** (-0.00071)
TG	MX_L1_L1	0.00000 ± 0.00000*** (-0.00022)	0.00000 ± 0.00000*** (-0.00205)
TG	MX_L1_LX	0.00000 ± 0.00000*** (-0.00111)	0.00000 ± 0.00000*** (-0.00034)
TG	MX_LX_LX	0.00000 ± 0.00000*** (-0.0014)	0.00000 ± 0.00000*** (-1e-05)
TG	L0_L0_L0	0.00000 ± 0.00000*** (-0.00557)	0.00000 ± 0.00000*** (-0.00071)

TG	L0_L0_L1	0.00000 ± 0.00000*** (-0.00111)	0.00000 ± 0.00000*** (-0.00101)
TG	L0_L0_LX	0.00000 ± 0.00000*** (-0.00281)	0.00000 ± 0.00000*** (-8e-05)
TG	L0_L1_L1	0.00000 ± 0.00000*** (-7e-05)	0.00000 ± 0.00000*** (-0.00047)
TG	L0_L1_LX	0.00000 ± 0.00000*** (-0.00037)	0.00000 ± 0.00000*** (-8e-05)
TG	L0_LX_LX	0.00000 ± 0.00000*** (-0.00047)	0.00000 ± 0.00000*** (0)
TG	L1_L1_L1	0.00000 ± 0.00000*** (0)	0.00000 ± 0.00000*** (-7e-05)
TG	L1_L1_LX	0.00000 ± 0.00000*** (-1e-05)	0.00000 ± 0.00000*** (-2e-05)
TG	L1_LX_LX	0.00000 ± 0.00000*** (-3e-05)	0.00000 ± 0.00000*** (0)
TG	LX_LX_LX	0.00000 ± 0.00000*** (-3e-05)	0.00000 ± 0.00000*** (0)
**Phospholipids**			
CL	S0_S0_S0_S0	0.00000 ± 0.00000*** (-0.00032)	0.00000 ± 0.00000*** (-0.00014)
CL	S0_S0_S0_S1	0.00000 ± 0.00000*** (-0.00024)	0.00000 ± 0.00000*** (-1e-04)
CL	S0_S0_S0_M0	0.00000 ± 0.00000*** (-0.00014)	0.00000 ± 0.00000*** (-1e-04)
CL	S0_S0_S0_M1	0.00000 ± 0.00000*** (-0.00867)	0.00000 ± 0.00000*** (-0.00817)
CL	S0_S0_S0_MX	0.00000 ± 0.00000*** (-0.01788)	0.00000 ± 0.00000*** (-0.00644)
CL	S0_S0_S0_LX	0.00000 ± 0.00000*** (-0.00232)	0.00000 ± 0.00000*** (-0.00087)
CL	S0_S0_S1_S1	0.00000 ± 0.00000*** (-6e-05)	0.00000 ± 0.00000*** (-2e-05)
CL	S0_S0_S1_M0	0.00000 ± 0.00000*** (-7e-05)	0.00000 ± 0.00000*** (-5e-05)
CL	S0_S0_S1_M1	0.00000 ± 0.00000*** (-0.00472)	0.00000 ± 0.00000*** (-0.00423)
CL	S0_S0_S1_MX	0.00000 ± 0.00000*** (-0.00974)	0.00000 ± 0.00000*** (-0.00333)
CL	S0_S0_S1_LX	0.00000 ± 0.00000*** (-0.00127)	0.00000 ± 0.00000*** (-0.00045)
CL	S0_S0_M0_M0	0.00000 ± 0.00000*** (-2e-05)	0.00000 ± 0.00000*** (-3e-05)
CL	S0_S0_M0_M1	0.00000 ± 0.00000*** (-0.00276)	0.00000 ± 0.00000*** (-0.00432)
CL	S0_S0_M0_MX	0.00000 ± 0.00000*** (-0.00569)	0.00000 ± 0.00000*** (-0.0034)
CL	S0_S0_M0_LX	0.00000 ± 0.00000*** (-0.00074)	0.00000 ± 0.00000*** (-0.00046)
CL	S0_S0_M1_M1	0.00000 ± 0.00000*** (-0.08697)	0.00000 ± 0.00000*** (-0.1796)
CL	S0_S0_M1_MX	0.00000 ± 0.00000*** (-0.35886)	0.00000 ± 0.00000*** (-0.28316)
CL	S0_S0_M1_LX	0.00000 ± 0.00000*** (-0.04662)	0.00000 ± 0.00000*** (-0.03813)
CL	S0_S0_MX_MX	0.00000 ± 0.00000*** (-0.3702)	0.00000 ± 0.00000*** (-0.1116)
CL	S0_S0_MX_LX	0.00000 ± 0.00000*** (-0.0962)	0.00000 ± 0.00000*** (-0.03005)
CL	S0_S0_LX_LX	0.00000 ± 0.00000*** (-0.00625)	0.00000 ± 0.00000*** (-0.00202)
CL	S0_S1_S1_S1	0.00000 ± 0.00000*** (-1e-05)	0.00000 ± 0.00000*** (0)
CL	S0_S1_S1_M0	0.00000 ± 0.00000*** (-1e-05)	0.00000 ± 0.00000*** (-1e-05)
CL	S0_S1_S1_M1	0.00000 ± 0.00000*** (-0.00086)	0.00000 ± 0.00000*** (-0.00073)
CL	S0_S1_S1_MX	0.00000 ± 0.00000*** (-0.00177)	0.00000 ± 0.00000*** (-0.00057)
CL	S0_S1_S1_LX	0.00000 ± 0.00000*** (-0.00023)	0.00000 ± 0.00000*** (-8e-05)
CL	S0_S1_M0_M0	0.00000 ± 0.00000*** (-1e-05)	0.00000 ± 0.00000*** (-1e-05)
CL	S0_S1_M0_M1	0.00000 ± 0.00000*** (-0.001)	0.00000 ± 0.00000*** (-0.00149)
CL	S0_S1_M0_MX	0.00000 ± 0.00000*** (-0.00207)	0.00000 ± 0.00000*** (-0.00117)
CL	S0_S1_M0_LX	0.00000 ± 0.00000*** (-0.00027)	0.00000 ± 0.00000*** (-0.00016)
CL	S0_S1_M1_M1	0.00000 ± 0.00000*** (-0.03159)	0.00000 ± 0.00000*** (-0.06193)
CL	S0_S1_M1_MX	0.00000 ± 0.00000*** (-0.13035)	0.00000 ± 0.00000*** (-0.09764)
CL	S0_S1_M1_LX	0.00000 ± 0.00000*** (-0.01694)	0.00000 ± 0.00000*** (-0.01315)
CL	S0_S1_MX_MX	0.00000 ± 0.00000*** (-0.13447)	0.00000 ± 0.00000*** (-0.03848)
CL	S0_S1_MX_LX	0.00000 ± 0.00000*** (-0.03494)	0.00000 ± 0.00000*** (-0.01036)
CL	S0_S1_LX_LX	0.00000 ± 0.00000*** (-0.00227)	0.00000 ± 0.00000*** (-7e-04)

CL	S0_M0_M0_M0	0.00000 ± 0.00000*** (0)	0.00000 ± 0.00000*** (0)
CL	S0_M0_M0_M1	0.00000 ± 0.00000*** (-0.00029)	0.00000 ± 0.00000*** (-0.00076)
CL	S0_M0_M0_MX	0.00000 ± 0.00000*** (-6e-04)	0.00000 ± 0.00000*** (-6e-04)
CL	S0_M0_M0_LX	0.00000 ± 0.00000*** (-8e-05)	0.00000 ± 0.00000*** (-8e-05)
CL	S0_M0_M1_M1	0.00000 ± 0.00000*** (-0.01844)	0.00000 ± 0.00000*** (-0.06322)
CL	S0_M0_M1_MX	0.00000 ± 0.00000*** (-0.07608)	0.00000 ± 0.00000*** (-0.09967)
CL	S0_M0_M1_LX	0.00000 ± 0.00000*** (-0.00988)	0.00000 ± 0.00000*** (-0.01342)
CL	S0_M0_MX_MX	0.00000 ± 0.00000*** (-0.07849)	0.00000 ± 0.00000*** (-0.03928)
CL	S0_M0_MX_LX	0.00000 ± 0.00000*** (-0.02039)	0.00000 ± 0.00000*** (-0.01058)
CL	S0_M0_LX_LX	0.00000 ± 0.00000*** (-0.00132)	0.00000 ± 0.00000*** (-0.00071)
CL	S0_M1_M1_M1	0.00000 ± 0.00000*** (-0.38784)	0.00000 ± 0.00000*** (-1.75376)
CL	S0_M1_M1_MX	0.00000 ± 0.00000*** (-2.40058)	0.00000 ± 0.00000*** (-4.1474)
CL	S0_M1_M1_LX	7.32839 ± 0.66833*** (1.97378)	11.62641 ± 0.47273*** (5.93405)
CL	S0_M1_MX_MX	0.00000 ± 0.00000*** (-4.95288)	0.00000 ± 0.00000*** (-3.26933)
CL	S0_M1_MX_LX	5.22303 ± 0.37960*** (5.43501)	6.09769 ± 0.86656*** (4.48799)
CL	S0_M1_LX_LX	0.00000 ± 0.00000*** (-0.08361)	0.00000 ± 0.00000*** (-0.05927)
CL	S0_MX_MX_MX	0.00000 ± 0.00000*** (-3.40625)	0.00000 ± 0.00000*** (-0.85906)
CL	S0_MX_MX_LX	5.99683 ± 0.82622*** (6.63408)	5.43716 ± 0.73467*** (1.5397)
CL	S0_MX_LX_LX	0.00000 ± 0.00000*** (-0.17249)	0.00000 ± 0.00000*** (-0.04672)
CL	S0_LX_LX_LX	0.00000 ± 0.00000*** (-0.00747)	0.00000 ± 0.00000*** (-0.0021)
CL	S1_S1_S1_S1	0.00000 ± 0.00000*** (0)	0.00000 ± 0.00000*** (0)
CL	S1_S1_S1_M0	0.00000 ± 0.00000*** (0)	0.00000 ± 0.00000*** (0)
CL	S1_S1_S1_M1	0.00000 ± 0.00000*** (-5e-05)	0.00000 ± 0.00000*** (-4e-05)
CL	S1_S1_S1_MX	0.00000 ± 0.00000*** (-0.00011)	0.00000 ± 0.00000*** (-3e-05)
CL	S1_S1_S1_LX	0.00000 ± 0.00000*** (-1e-05)	0.00000 ± 0.00000*** (0)
CL	S1_S1_M0_M0	0.00000 ± 0.00000*** (0)	0.00000 ± 0.00000*** (0)
CL	S1_S1_M0_M1	0.00000 ± 0.00000*** (-9e-05)	0.00000 ± 0.00000*** (-0.00013)
CL	S1_S1_M0_MX	0.00000 ± 0.00000*** (-0.00019)	0.00000 ± 0.00000*** (-1e-04)
CL	S1_S1_M0_LX	0.00000 ± 0.00000*** (-2e-05)	0.00000 ± 0.00000*** (-1e-05)
CL	S1_S1_M1_M1	0.00000 ± 0.00000*** (-0.00287)	0.00000 ± 0.00000*** (-0.00534)
CL	S1_S1_M1_MX	0.00000 ± 0.00000*** (-0.01184)	0.00000 ± 0.00000*** (-0.00842)
CL	S1_S1_M1_LX	0.00000 ± 0.00000*** (-0.00154)	0.00000 ± 0.00000*** (-0.00113)
CL	S1_S1_MX_MX	0.00000 ± 0.00000*** (-0.01221)	0.00000 ± 0.00000*** (-0.00332)
CL	S1_S1_MX_LX	0.00000 ± 0.00000*** (-0.00317)	0.00000 ± 0.00000*** (-0.00089)
CL	S1_S1_LX_LX	0.00000 ± 0.00000*** (-0.00021)	0.00000 ± 0.00000*** (-6e-05)
CL	S1_M0_M0_M0	0.00000 ± 0.00000*** (0)	0.00000 ± 0.00000*** (0)
CL	S1_M0_M0_M1	0.00000 ± 0.00000*** (-5e-05)	0.00000 ± 0.00000*** (-0.00013)
CL	S1_M0_M0_MX	0.00000 ± 0.00000*** (-0.00011)	0.00000 ± 0.00000*** (-1e-04)
CL	S1_M0_M0_LX	0.00000 ± 0.00000*** (-1e-05)	0.00000 ± 0.00000*** (-1e-05)
CL	S1_M0_M1_M1	0.00000 ± 0.00000*** (-0.00335)	0.00000 ± 0.00000*** (-0.0109)
CL	S1_M0_M1_MX	0.00000 ± 0.00000*** (-0.01382)	0.00000 ± 0.00000*** (-0.01718)
CL	S1_M0_M1_LX	0.00000 ± 0.00000*** (-0.0018)	0.00000 ± 0.00000*** (-0.00231)
CL	S1_M0_MX_MX	0.00000 ± 0.00000*** (-0.01425)	0.00000 ± 0.00000*** (-0.00677)
CL	S1_M0_MX_LX	0.00000 ± 0.00000*** (-0.0037)	0.00000 ± 0.00000*** (-0.00182)
CL	S1_M0_LX_LX	0.00000 ± 0.00000*** (-0.00024)	0.00000 ± 0.00000*** (-0.00012)
CL	S1_M1_M1_M1	0.00000 ± 0.00000*** (-0.07044)	0.00000 ± 0.00000*** (-0.30236)

CL	S1_M1_M1_MX	0.00000 ± 0.00000*** (-0.43598)	0.00000 ± 0.00000*** (-0.71504)
CL	S1_M1_M1_LX	0.00000 ± 0.00000*** (-0.05664)	0.00000 ± 0.00000*** (-0.09628)
CL	S1_M1_MX_MX	0.00000 ± 0.00000*** (-0.89952)	0.00000 ± 0.00000*** (-0.56366)
CL	S1_M1_MX_LX	3.40604 ± 0.13701*** (0.56238)	6.63996 ± 0.32161*** (0.85607)
CL	S1_M1_LX_LX	0.00000 ± 0.00000*** (-0.01518)	0.00000 ± 0.00000*** (-0.01022)
CL	S1_MX_MX_MX	0.00000 ± 0.00000*** (-0.61863)	0.00000 ± 0.00000*** (-0.14811)
CL	S1_MX_MX_LX	9.47975 ± 0.53778*** (2.04468)	22.77155 ± 1.44350*** (1.3025)
CL	S1_MX_LX_LX	0.00000 ± 0.00000*** (-0.03133)	0.00000 ± 0.00000*** (-0.00806)
CL	S1_LX_LX_LX	0.00000 ± 0.00000*** (-0.00136)	0.00000 ± 0.00000*** (-0.00036)
CL	M0_M0_M0_M0	0.00000 ± 0.00000*** (0)	0.00000 ± 0.00000*** (0)
CL	M0_M0_M0_M1	0.00000 ± 0.00000*** (-1e-05)	0.00000 ± 0.00000*** (-4e-05)
CL	M0_M0_M0_MX	0.00000 ± 0.00000*** (-2e-05)	0.00000 ± 0.00000*** (-4e-05)
CL	M0_M0_M0_LX	0.00000 ± 0.00000*** (0)	0.00000 ± 0.00000*** (0)
CL	M0_M0_M1_M1	0.00000 ± 0.00000*** (-0.00098)	0.00000 ± 0.00000*** (-0.00556)
CL	M0_M0_M1_MX	0.00000 ± 0.00000*** (-0.00403)	0.00000 ± 0.00000*** (-0.00877)
CL	M0_M0_M1_LX	0.00000 ± 0.00000*** (-0.00052)	0.00000 ± 0.00000*** (-0.00118)
CL	M0_M0_MX_MX	0.00000 ± 0.00000*** (-0.00416)	0.00000 ± 0.00000*** (-0.00346)
CL	M0_M0_MX_LX	0.00000 ± 0.00000*** (-0.00108)	0.00000 ± 0.00000*** (-0.00093)
CL	M0_M0_LX_LX	0.00000 ± 0.00000*** (-7e-05)	0.00000 ± 0.00000*** (-6e-05)
CL	M0_M1_M1_M1	0.00000 ± 0.00000*** (-0.04111)	0.00000 ± 0.00000*** (-0.30865)
CL	M0_M1_M1_MX	0.00000 ± 0.00000*** (-0.25448)	0.00000 ± 0.00000*** (-0.72992)
CL	M0_M1_M1_LX	0.00000 ± 0.00000*** (-0.03306)	0.00000 ± 0.00000*** (-0.09828)
CL	M0_M1_MX_MX	3.42617 ± 0.53312*** (1.27383)	4.20501 ± 0.34737*** (1.84412)
CL	M0_M1_MX_LX	0.00000 ± 0.00000*** (-0.13643)	0.00000 ± 0.00000*** (-0.15495)
CL	M0_M1_LX_LX	0.00000 ± 0.00000*** (-0.00886)	0.00000 ± 0.00000*** (-0.01043)
CL	M0_MX_MX_MX	0.00000 ± 0.00000*** (-0.36108)	0.00000 ± 0.00000*** (-0.15119)
CL	M0_MX_MX_LX	0.00000 ± 0.00000*** (-0.14074)	0.00000 ± 0.00000*** (-0.06107)
CL	M0_MX_LX_LX	0.00000 ± 0.00000*** (-0.01829)	0.00000 ± 0.00000*** (-0.00822)
CL	M0_LX_LX_LX	0.00000 ± 0.00000*** (-0.00079)	0.00000 ± 0.00000*** (-0.00037)
CL	M1_M1_M1_M1	7.51238 ± 0.95533*** (4.22401)	2.78724 ± 0.15779*** (11.47736)
CL	M1_M1_M1_MX	0.79658 ± 0.09717 (-1.08888)	0.67052 ± 0.05055*** (-6.67168)
CL	M1_M1_M1_LX	0.00000 ± 0.00000*** (-0.69546)	0.00000 ± 0.00000*** (-2.72642)
CL	M1_M1_MX_MX	0.92481 ± 0.06733 (-1.24556)	0.90796 ± 0.02148** (-2.20376)
CL	M1_M1_MX_LX	1.92339 ± 0.10430*** (3.97484)	0.61221 ± 0.10340* (-2.50031)
CL	M1_M1_LX_LX	0.00000 ± 0.00000*** (-0.27964)	0.00000 ± 0.00000*** (-0.43407)
CL	M1_MX_MX_MX	0.79204 ± 0.03198*** (-4.7386)	1.15805 ± 0.03827** (1.98863)
CL	M1_MX_MX_LX	0.23623 ± 0.01089*** (-6.78325)	0.25717 ± 0.01306*** (-3.77545)
CL	M1_MX_LX_LX	0.00000 ± 0.00000*** (-1.15389)	0.00000 ± 0.00000*** (-0.68434)
CL	M1_LX_LX_LX	0.00000 ± 0.00000*** (-0.04997)	0.00000 ± 0.00000*** (-0.03071)
CL	MX_MX_MX_MX	2.14993 ± 0.11107*** (13.5151)	3.39618 ± 0.22658*** (5.94172)
CL	MX_MX_MX_LX	0.00000 ± 0.00000*** (-6.10794)	0.00000 ± 0.00000*** (-1.3355)
CL	MX_MX_LX_LX	0.00000 ± 0.00000*** (-1.19035)	0.00000 ± 0.00000*** (-0.26973)
CL	MX_LX_LX_LX	0.00000 ± 0.00000*** (-0.1031)	0.00000 ± 0.00000*** (-0.02421)
CL	LX_LX_LX_LX	0.00000 ± 0.00000*** (-0.00335)	0.00000 ± 0.00000*** (-0.00081)
PC	S0_S0	0.00000 ± 0.00000*** (-0.01128)	0.00000 ± 0.00000*** (-0.02024)
PC	S0_M0	0.70266 ± 0.01690*** (-0.23947)	0.67707 ± 0.02064*** (-0.22914)

PC	S0_M1	1.16704 ± 0.04912** (0.12558)	0.74857 ± 0.02633*** (-0.46901)
PC	S0_MX	0.81266 ± 0.05348** (-0.10151)	1.07889 ± 0.04693 (0.01686)
PC	S0_L1	94.15673 ± 7.03738*** (0.0883)	70.29526 ± 16.39599*** (0.4346)
PC	S0_LX	94.15673 ± 9.46792*** (0.14967)	29.98236 ± 2.07887*** (0.28715)
PC	M0_M0	0.37088 ± 0.01566*** (-9.04444)	0.61046 ± 0.04694*** (-2.42267)
PC	M0_M1	1.32281 ± 0.04205*** (8.66365)	1.06993 ± 0.02272* (2.2868)
PC	M0_MX	1.50432 ± 0.03230*** (9.75589)	1.71343 ± 0.09012*** (2.6719)
PC	M0_L1	0.00000 ± 0.00000*** (-0.03384)	0.00000 ± 0.00000*** (-0.10995)
PC	M0_LX	0.00000 ± 0.00000*** (-0.05736)	2.29957 ± 0.63882 (0.22573)
PC	M1_M1	1.13021 ± 0.07238 (1.631)	1.04725 ± 0.02232 (2.03108)
PC	M1_MX	0.33731 ± 0.02370*** (-11.96611)	0.47852 ± 0.03485*** (-5.1343)
PC	M1_L1	0.00000 ± 0.00000*** (-0.03159)	0.00000 ± 0.00000*** (-0.28904)
PC	M1_LX	0.00000 ± 0.00000*** (-0.05354)	0.00000 ± 0.00000*** (-0.45662)
PC	MX_MX	1.18234 ± 0.15646 (1.18655)	3.24453 ± 0.18910*** (1.26548)
PC	MX_L1	0.00000 ± 0.00000*** (-0.02277)	0.00000 ± 0.00000*** (-0.0331)
PC	MX_LX	0.00000 ± 0.00000*** (-0.03859)	0.00000 ± 0.00000*** (-0.0523)
PC	L1_L1	0.00000 ± 0.00000*** (-2e-05)	0.00000 ± 0.00000*** (-0.00049)
PC	L1_LX	0.00000 ± 0.00000*** (-7e-05)	0.00000 ± 0.00000*** (-0.00154)
PC	LX_LX	0.00000 ± 0.00000*** (-6e-05)	0.00000 ± 0.00000*** (-0.00121)
PE	S0_S0	-	0.00000 ± 0.00000*** (-1e-05)
PE	S0_SX	-	0.00000 ± 0.00000*** (-2e-05)
PE	S0_M0	-	0.00000 ± 0.00000*** (-0.02368)
PE	S0_M1	-	0.00000 ± 0.00000*** (-0.02932)
PE	S0_MX	-	0.00000 ± 0.00000*** (-0.02027)
PE	S0_L0	-	0.00000 ± 0.00000*** (-0.00016)
PE	S0_L1	-	30.62047 ± 21.06087 (0.0735)
PE	S0_LX	-	0.00000 ± 0.00000*** (-1e-05)
PE	SX_SX	0.00000 ± 0.00000*** (-5e-05)	0.00000 ± 0.00000*** (-1e-05)
PE	SX_M0	3.13956 ± 0.15896*** (0.09789)	3.20861 ± 0.14250*** (0.0429)
PE	SX_M1	0.00000 ± 0.00000*** (-0.05137)	0.00000 ± 0.00000*** (-0.02405)
PE	SX_MX	0.00000 ± 0.00000*** (-0.0459)	0.00000 ± 0.00000*** (-0.01663)
PE	SX_L0	0.00000 ± 0.00000*** (-0.00051)	0.00000 ± 0.00000*** (-0.00013)
PE	SX_L1	-	0.00000 ± 0.00000*** (-0.00204)
PE	SX_LX	-	0.00000 ± 0.00000*** (-1e-05)
PE	M0_M0	0.05989 ± 0.00438*** (-9.53772)	0.04036 ± 0.00189*** (-9.32128)
PE	M0_M1	1.45774 ± 0.02849*** (10.42849)	0.99571 ± 0.03298 (-0.10311)
PE	M0_MX	1.43116 ± 0.02380*** (8.77696)	2.25566 ± 0.06027*** (20.88441)
PE	M0_L0	0.00000 ± 0.00000*** (-0.2279)	0.00000 ± 0.00000*** (-0.13491)
PE	M0_L1	-	0.00000 ± 0.00000*** (-2.03564)
PE	M0_LX	-	2.37327 ± 1.66384 (0.01258)
PE	M1_M1	0.87770 ± 0.03644** (-1.56429)	1.29087 ± 0.03194*** (4.33043)
PE	M1_MX	0.67657 ± 0.03337*** (-7.39251)	0.38851 ± 0.03311*** (-12.59123)
PE	M1_L0	1.56259 ± 0.06145*** (0.14397)	1.97453 ± 0.13103*** (0.16277)
PE	M1_L1	-	2.56155 ± 0.75246 (3.93541)
PE	M1_LX	-	0.00000 ± 0.00000*** (-0.01134)
PE	MX_MX	0.93020 ± 0.06969 (-0.71278)	0.54347 ± 0.03160*** (-3.25042)

PE	MX_L0	1.38056 ± 0.11447** (0.08701)	0.89245 ± 0.14324 (-0.01243)
PE	MX_L1	-	0.00000 ± 0.00000*** (-1.74283)
PE	MX_LX	-	0.97568 ± 0.39048 (-0.00019)
PE	L0_L0	0.00000 ± 0.00000*** (-0.00128)	0.00000 ± 0.00000*** (-0.00047)
PE	L0_L1	-	0.00000 ± 0.00000*** (-0.01414)
PE	L0_LX	-	0.00000 ± 0.00000*** (-6e-05)
PE	L1_L1	-	0.00000 ± 0.00000*** (-0.10665)
PE	L1_LX	-	0.00000 ± 0.00000*** (-0.00096)
PE	LX_LX	-	0.00000 ± 0.00000*** (0)
PG	SX_SX	0.00000 ± 0.00000*** (-1.05563)	0.00000 ± 0.00000*** (-0.89723)
PG	SX_M0	0.00000 ± 0.00000*** (-6.45117)	0.00000 ± 0.00000*** (-6.01944)
PG	SX_M1	0.00000 ± 0.00000*** (-2.95901)	0.00000 ± 0.00000*** (-4.89579)
PG	SX_MX	0.00000 ± 0.00000*** (-6.91606)	0.00000 ± 0.00000*** (-4.44029)
PG	SX_LX	9.73295 ± 0.86605*** (18.4375)	10.55719 ± 0.74180*** (17.14997)
PG	M0_M0	0.00000 ± 0.00000*** (-9.85612)	0.00000 ± 0.00000*** (-10.09599)
PG	M0_M1	1.50640 ± 0.11932*** (4.57865)	1.81370 ± 0.05459*** (13.36311)
PG	M0_MX	2.32666 ± 0.07497*** (28.03593)	2.26674 ± 0.08359*** (18.86776)
PG	M0_LX	0.00000 ± 0.00000*** (-6.45117)	0.00000 ± 0.00000*** (-6.01944)
PG	M1_M1	0.26952 ± 0.04212*** (-1.51473)	0.84019 ± 0.07065 (-1.06727)
PG	M1_MX	1.45071 ± 0.08148*** (4.36881)	0.88138 ± 0.07281 (-1.43699)
PG	M1_LX	0.00000 ± 0.00000*** (-2.95901)	0.00000 ± 0.00000*** (-4.89579)
PG	MX_MX	0.18022 ± 0.02885*** (-9.28631)	0.22181 ± 0.03807*** (-4.27509)
PG	MX_LX	0.00000 ± 0.00000*** (-6.91606)	0.00000 ± 0.00000*** (-4.44029)
PG	LX_LX	0.00000 ± 0.00000*** (-1.05563)	0.00000 ± 0.00000*** (-0.89723)
PI	M0_M0	0.00000 ± 0.00000*** (-9.42136)	0.00000 ± 0.00000*** (-12.3215)
PI	M0_M1	1.10747 ± 0.07262 (1.69616)	1.43260 ± 0.04013*** (9.24911)
PI	M0_MX	1.64067 ± 0.05456*** (17.14657)	1.63661 ± 0.06156*** (15.39389)
PI	M1_M1	0.17905 ± 0.01803*** (-5.42596)	0.67257 ± 0.02693*** (-3.03677)
PI	M1_MX	1.40844 ± 0.09265*** (9.15577)	0.84863 ± 0.07598 (-3.17556)
PI	MX_MX	0.30809 ± 0.02278*** (-13.15117)	0.48506 ± 0.03953*** (-6.10917)
PS	S1_S1	-	-
PS	S1_M0	-	-
PS	S1_M1	-	-
PS	S1_MX	-	-
PS	S1_L0	-	-
PS	S1_L1	-	-
PS	S1_LX	-	-
PS	M0_M0	0.00000 ± 0.00000*** (-1.86838)	0.00000 ± 0.00000*** (-1.00703)
PS	M0_M1	1.84737 ± 0.06467*** (11.70595)	1.65572 ± 0.10097*** (7.41227)
PS	M0_MX	0.18668 ± 0.08754*** (-7.91792)	0.30237 ± 0.14794*** (-3.12353)
PS	M0_L0	-	0.00000 ± 0.00000*** (-1.86496)
PS	M0_L1	0.00000 ± 0.00000*** (-0.05126)	0.00000 ± 0.00000*** (-0.04063)
PS	M0_LX	-	0.00000 ± 0.00000*** (-0.3691)
PS	M1_M1	0.78891 ± 0.12725 (-5.39032)	0.85243 ± 0.13472 (-4.68143)
PS	M1_MX	0.96914 ± 0.06097 (-1.11081)	0.70730 ± 0.05358*** (-7.35529)
PS	M1_L0	-	1.77548 ± 0.52956 (8.11716)

PS	M1_L1	1.97893 ± 1.52369 (0.1855)	1.77548 ± 1.77548 (0.17686)
PS	M1_LX	-	1.48844 ± 0.45092 (1.01185)
PS	MX_MX	1.36124 ± 0.06338*** (4.58114)	2.50109 ± 0.15448*** (7.47046)
PS	MX_L0	-	0.00000 ± 0.00000*** (-4.1459)
PS	MX_L1	0.00000 ± 0.00000*** (-0.13354)	0.00000 ± 0.00000*** (-0.09033)
PS	MX_LX	-	0.72471 ± 0.23698 (-0.22588)
PS	L0_L0	-	0.00000 ± 0.00000*** (-0.86345)
PS	L0_L1	-	0.00000 ± 0.00000*** (-0.03763)
PS	L0_LX	-	0.00000 ± 0.00000*** (-0.34178)
PS	L1_L1	0.00000 ± 0.00000*** (-0.00035)	0.00000 ± 0.00000*** (-0.00041)
PS	L1_LX	-	0.00000 ± 0.00000*** (-0.00745)
PS	LX_LX	-	0.00000 ± 0.00000*** (-0.03382)

Table S14: Differences between observed and expected frequencies of combinations of acyl chains in the three chain length categories in each ester lipid class. Differences in the percentages are first expressed as ratios (± SE) of the percentages of observed vs expected, with the absolute difference given in brackets. Category abbreviations are as defined in the text. * Bonferroni-corrected P < 0.05; ** Bonferroni-corrected P < 0.01; *** Bonferroni-corrected P < 0.001.

Class	Category	Day1	Day19
		**Neutral lipids**	
**DG**	SS	0.00000 ± 0.00000*** (-0.13313)	0.00000 ± 0.00000*** (-0.17581)
**DG**	SM	0.00000 ± 0.00000*** (-6.0917)	0.00000 ± 0.00000*** (-6.60798)
**DG**	SL	7.76704 ± 3.07119 (6.35797)	5.87916 ± 0.79636*** (6.95961)
**DG**	MM	1.06554 ± 0.06683 (4.56719)	1.06266 ± 0.09041 (3.89065)
**DG**	ML	0.85845 ± 0.12051 (-3.0427)	0.95623 ± 0.20291 (-1.17332)
**DG**	LL	0.00000 ± 0.00000*** (-1.65764)	0.00000 ± 0.00000*** (-2.89314)
**TG**	SSS	3.23574 ± 0.83619* (0.43213)	6.70719 ± 1.26990*** (0.40417)
**TG**	SSM	1.60860 ± 0.23782 (2.34651)	0.68046 ± 0.07645*** (-0.67163)
**TG**	SSL	2.60334 ± 0.41647*** (0.35199)	2.13598 ± 0.70599 (0.07865)
**TG**	SMM	0.77971 ± 0.06886** (-5.64765)	1.03873 ± 0.07727 (0.80542)
**TG**	SML	0.05412 ± 0.01336*** (-2.76153)	0.00299 ± 0.00087*** (-1.36583)
**TG**	SLL	21.64245 ± 7.04447* (1.71579)	24.65879 ± 2.39124*** (0.53384)
**TG**	MMM	1.10977 ± 0.05317 (6.23768)	1.00072 ± 0.02721 (0.04918)
**TG**	MML	0.55305 ± 0.03578*** (-4.33829)	1.01629 ± 0.12212 (0.11041)
**TG**	MLL	4.02856 ± 0.80821*** (1.67385)	1.26087 ± 0.16886 (0.05824)
**TG**	LLL	0.00000 ± 0.00000*** (-0.01049)	0.00000 ± 0.00000*** (-0.00245)
		**Phospholipids**	
**CL**	SSSS	0.00000 ± 0.00000*** (-0.00063)	0.00000 ± 0.00000*** (-0.00026)
**CL**	SSSM	0.00000 ± 0.00000*** (-0.04403)	0.00000 ± 0.00000*** (-0.02372)
**CL**	SSSL	0.00000 ± 0.00000*** (-0.00383)	0.00000 ± 0.00000*** (-0.0014)
**CL**	SSMM	0.00000 ± 0.00000*** (-1.15117)	0.00000 ± 0.00000*** (-0.80013)
**CL**	SSML	0.00000 ± 0.00000*** (-0.20044)	0.00000 ± 0.00000*** (-0.09435)
**CL**	SSLL	0.00000 ± 0.00000*** (-0.00873)	0.00000 ± 0.00000*** (-0.00278)
**CL**	SMMM	0.00000 ± 0.00000*** (-13.37761)	0.00000 ± 0.00000*** (-11.99735)
**CL**	SMML	5.73892 ± 0.37988*** (16.5574)	7.59575 ± 0.55510*** (13.99581)
**CL**	SMLL	0.00000 ± 0.00000*** (-0.30418)	0.00000 ± 0.00000*** (-0.1251)
**CL**	SLLL	0.00000 ± 0.00000*** (-0.00883)	0.00000 ± 0.00000*** (-0.00246)
**CL**	MMMM	1.19339 ± 0.03042*** (11.27402)	1.16556 ± 0.02682*** (11.16878)
**CL**	MMML	0.51118 ± 0.02290*** (-9.92366)	0.33029 ± 0.04138*** (-10.65409)
**CL**	MMLL	0.00000 ± 0.00000*** (-2.65109)	0.00000 ± 0.00000*** (-1.40685)
**CL**	MLLL	0.00000 ± 0.00000*** (-0.15387)	0.00000 ± 0.00000*** (-0.05529)
**CL**	LLLL	0.00000 ± 0.00000*** (-0.00335)	0.00000 ± 0.00000*** (-0.00081)
**PC**	SS	0.00000 ± 0.00000*** (-0.01128)	0.00000 ± 0.00000*** (-0.02024)
**PC**	SM	0.89737 ± 0.01753*** (-0.21541)	0.75568 ± 0.02105*** (-0.68129)
**PC**	SL	94.15673 ± 7.77655*** (0.23798)	45.60892 ± 5.89867*** (0.72176)
**PC**	MM	1.00232 ± 0.00055*** (0.22655)	1.00727 ± 0.00164*** (0.69829)
**PC**	ML	0.00000 ± 0.00000*** (-0.23768)	0.35832 ± 0.09954*** (-0.71529)
**PC**	LL	0.00000 ± 0.00000*** (-0.00014)	0.00000 ± 0.00000*** (-0.00323)

**PE**	SS	0.00000 ± 0.00000*** (-5e-05)	0.00000 ± 0.00000*** (-5e-05)
**PE**	SM	1.00431 ± 0.05085 (0.00061)	0.46728 ± 0.02075*** (-0.07105)
**PE**	SL	0.00000 ± 0.00000*** (-0.00051)	15.71056 ±10.80578 (0.07114)
**PE**	MM	0.99998 ± 0.00035 (-0.00185)	0.99945 ± 0.02081 (-0.05119)
**PE**	ML	1.00431 ± 0.05300 (0.00308)	1.02571 ± 0.28354 (0.17343)
**PE**	LL	0.00000 ± 0.00000*** (-0.00128)	0.00000 ± 0.00000*** (-0.12229)
**PG**	SS	0.00000 ± 0.00000*** (-1.05563)	0.00000 ± 0.00000*** (-0.89723)
**PG**	SM	0.00000 ± 0.00000*** (-16.32624)	0.00000 ± 0.00000*** (-15.35552)
**PG**	SL	9.73295 ± 0.86605*** (18.4375)	10.55719 ± 0.74180*** (17.14997)
**PG**	MM	1.25863 ± 0.02897*** (16.32624)	1.23372 ± 0.02026*** (15.35552)
**PG**	ML	0.00000 ± 0.00000*** (-16.32624)	0.00000 ± 0.00000*** (-15.35552)
**PG**	LL	0.00000 ± 0.00000*** (-1.05563)	0.00000 ± 0.00000*** (-0.89723)
**PI**	MM	1.00000 ± 0.00000 (0)	1.00000 ± 0.00000 (0)
**PS**	SS	-	-
**PS**	SM	-	-
**PS**	SL	-	-
**PS**	MM	1.00000 ± 0.00290 (-0.00035)	0.98366 ± 0.08351 (-1.28453)
**PS**	ML	1.00188 ± 0.77140 (7e-04)	1.12782 ± 0.32667 (2.56906)
**PS**	LL	0.00000 ± 0.00000*** (-0.00035)	0.00000 ± 0.00000*** (-1.28453)

Table S15: Differences between observed and expected frequencies of combinations of acyl chains in the three double bond categories in each ester lipid class. Differences in the percentages are first expressed as ratios (± SE) of the percentages of observed vs expected, with the absolute difference given in brackets. Category abbreviations are as defined in the text. * Bonferroni-corrected P < 0.05; ** Bonferroni-corrected P < 0.01; *** Bonferroni-corrected P < 0.001.

Class	Category	Day1	Day19
		**Neutral lipids**	
**DG**	1	0.79408 ±0.10567 (-5.00591)	1.08783 ±0.17473 (1.60288)
**DG**	11	1.26611 ±0.19239 (7.09185)	0.63514 ±0.17630 (-6.51122)
**DG**	0	0.00000 ±0.00000*** (-5.54364)	0.00000 ±0.00000*** (-4.66522)
**DG**	0X	2.37631 ±0.66008 (16.0932)	1.49475 ±0.25870 (7.72756)
**DG**	1X	0.64202 ±0.19151 (-9.17777)	1.37382 ±0.15937~ (11.41955)
**DG**	XX	0.43922 ±0.38319 (-3.45771)	0.26768 ±0.03637*** (-9.57356)
**TG**	0	0.15327 ±0.01833*** (-7.17952)	0.22559 ±0.02374*** (-11.56106)
**TG**	1	1.32267 ±0.09840** (7.42426)	1.30175 ±0.09851* (9.73309)
**TG**	11	1.01225 ±0.09750 (0.25499)	0.99552 ±0.11932 (-0.10406)
**TG**	111	1.46233 ±0.19757~ (2.901)	1.05923 ±0.14315 (0.33032)
**TG**	00X	1.84638 ±0.25501** (8.0029)	2.30347 ±0.39306** (9.62392)
**TG**	01X	0.35320 ±0.07155*** (-11.06359)	0.71078 ±0.06038*** (-3.07579)
**TG**	11X	0.61138 ±0.08104*** (-3.00632)	0.11588 ±0.01822*** (-3.38583)
**TG**	0XX	1.42474 ±0.16492~ (1.49284)	0.30087 ±0.01896*** (-0.85096)
**TG**	1XX	1.13808 ±0.17759 (0.43898)	0.23748 ±0.02110*** (-0.66844)
**TG**	XXX	2.68652 ±0.61998* (0.73446)	0.38457 ±0.10284*** (-0.04117)
		**Phospholipids**	
**CL**	1111	6.74905 ±0.85826*** (4.15065)	2.65978 ±0.15057*** (11.16962)
**CL**	0	0.00000 ±0.00000*** (-0.00048)	0.00000 ±0.00000*** (-0.00027)
**CL**	1	0.00000 ±0.00000*** (-0.01204)	0.00000 ±0.00000*** (-0.01345)
**CL**	11	0.00000 ±0.00000*** (-0.11224)	0.00000 ±0.00000*** (-0.25427)
**CL**	111	0.00000 ±0.00000*** (-0.46485)	0.00000 ±0.00000*** (-2.13611)
**CL**	011X	0.72214 ±0.06586*** (-0.87946)	1.14605 ±0.04660* (0.8274)
**CL**	111X	0.65055 ±0.07935*** (-2.29047)	0.57056 ±0.04302*** (-10.219)
**CL**	000X	0.00000 ±0.00000*** (-0.02734)	0.00000 ±0.00000*** (-0.01189)
**CL**	001X	0.00000 ±0.00000*** (-0.50948)	0.00000 ±0.00000*** (-0.44955)
**CL**	01XX	1.18614 ±0.06018* (1.33718)	1.55508 ±0.13516*** (2.77987)
**CL**	11XX	1.09329 ±0.03333* (2.08173)	0.84597 ±0.01640*** (-4.86021)
**CL**	00XX	0.00000 ±0.00000*** (-0.57816)	0.00000 ±0.00000*** (-0.19871)
**CL**	0XXX	1.46496 ±0.20184 (2.52697)	1.27849 ±0.17275 (0.41097)
**CL**	1XXX	0.66436 ±0.02192*** (-11.33236)	0.92709 ±0.03118 (-1.3559)
**CL**	XXXX	1.31895 ±0.06814*** (6.11036)	2.04904 ±0.13671*** (4.31147)
**PC**	0	0.38819 ±0.01506*** (-9.29519)	0.61548 ±0.04256*** (-2.67204)
**PC**	1	1.32013 ±0.04203*** (8.84368)	1.06177 ±0.02204* (2.14245)
**PC**	11	1.12737 ±0.07220 (1.5994)	1.04025 ±0.02217 (1.74155)
**PC**	0X	1.48867 ±0.03220*** (9.7467)	1.77290 ±0.07873*** (3.20163)
**PC**	1X	0.33588 ±0.02361*** (-12.04248)	0.45578 ±0.03319*** (-5.62556)

**PC**	XX	1.17535 ±0.15553 (1.1479)	2.96329 ±0.17271*** (1.21197)
**PE**	0	0.05856 ±0.00428*** (-9.7669)	0.03970 ±0.00186*** (-9.48051)
**PE**	1	1.45890 ±0.02835*** (10.57245)	0.92601 ±0.02961* (-1.94593)
**PE**	11	0.87770 ±0.03644** (-1.56429)	1.46585 ±0.09651*** (8.15919)
**PE**	0X	1.43436 ±0.02383*** (8.96134)	2.24470 ±0.05924*** (20.90696)
**PE**	1X	0.67505 ±0.03329*** (-7.44388)	0.35758 ±0.03047*** (-14.37244)
**PE**	XX	0.92603 ±0.06938 (-0.75873)	0.54268 ±0.03134*** (-3.26726)
**PG**	1	1.50640 ±0.11932*** (4.57865)	1.81370 ±0.05459*** (13.36311)
**PG**	11	0.26952 ±0.04212*** (-1.51473)	0.84019 ±0.07065~ (-1.06727)
**PG**	0	0.00000 ±0.00000*** (-9.85612)	0.00000 ±0.00000*** (-10.09599)
**PG**	0X	1.44465 ±0.04655*** (15.13359)	1.25354 ±0.04623*** (6.82888)
**PG**	1X	0.90076 ±0.05059 (-1.54921)	0.48742 ±0.04026*** (-11.22857)
**PG**	XX	0.76884 ±0.05424*** (-6.79219)	1.12246 ±0.06855 (2.19984)
**PI**	1	1.10747 ±0.07262 (1.69616)	1.43260 ±0.04013*** (9.24911)
**PI**	11	0.17905 ±0.01803*** (-5.42596)	0.67257 ±0.02693*** (-3.03677)
**PI**	0	0.00000 ±0.00000*** (-9.42136)	0.00000 ±0.00000*** (-12.3215)
**PI**	0X	1.64067 ±0.05456*** (17.14657)	1.63661 ±0.06156*** (15.39389)
**PI**	1X	1.40844 ±0.09265*** (9.15577)	0.84863 ±0.07598 (-3.17556)
**PI**	XX	0.30809 ±0.02278*** (-13.15117)	0.48506 ±0.03953*** (-6.10917)
**PS**	1	1.84054 ±0.06443*** (11.65469)	1.70716 ±0.21594** (15.45118)
**PS**	11	0.79766 ±0.12545 (-5.20517)	0.85900 ±0.13590 (-4.50498)
**PS**	0	0.00000 ±0.00000*** (-1.86838)	0.00000 ±0.00000*** (-3.73544)
**PS**	0X	0.18668 ±0.08754*** (-7.91792)	0.14504 ±0.07097*** (-7.98031)
**PS**	1X	0.96555 ±0.06074 (-1.24435)	0.76405 ±0.03920*** (-6.44121)
**PS**	XX	1.36124 ±0.06338*** (4.58114)	2.23662 ±0.11027*** (7.21076)

Table S16: Gene-enzyme systems in the Kennedy, ACYL-DHAP and Lands' pathways with direct effects on the identities of the acyl chains at the *sn1*, *sn2* and *sn3* positions. Enzymes are primarily classified on the basis of their Enzyme Commission (EC) numbers. Mammalian and insect gene-enzyme systems are represented by *H. sapiens* and *D. melanogaster*, respectively, and the top blastp matches for the latter in the *B. tryoni* reference proteins are also given. Human reference sequences for GPATs, LPLATs and DGAT1 reported in Valentine *et al*. [7] were used to identify their top blastp matches in *D. melanogaster*. Additional members of these enzyme classifications in *D. melanogaster* were extracted from the "Gene Group Lists" in Flybase (https://flybase.org/lists/FBgg/; Garapati *et al*. [132]). Dashes indicate the absence of identifiable orthologous sequences. Interspecific pairwise amino acid identity was calculated based on the optimal global alignment between two full-length homologues using EMBOSS Needle (https://www.ebi.ac.uk/Tools/psa/emboss_needle/).

		H. sapiens	***D. melanogaster***				***B. tryoni***			
**EC classification**	***sn* position**	***Hsap* gene: isoform**	***Dmel* protein ID**	***Dmel* gene-isoform**	***Dmel* gene-isoform name**	***Dmel/Hsap* % aa identity**	***Btry* gene**	***Btry* isoform**	***Dmel/Btry* % aa identity**	***Btry* LOC co-ordinates**
GPAT, EC 2.3.1.15	1	GPAT4: NM_178819	FBpp0072088	Gpat4-PB	Glycerol-3-phosphate acyltransferase 4	46.2	LOC120770141	XP_039953264.1	76.5	NC_052501.1 (87275142..87279648, complement)
		GPAT3: NM_001256421	FBpp0291375	Gpat4-PC		45.8		XP_039953263.1	74.3	
			FBpp0076972	CG15450-PA	-	38.6		XP_039953264.1	37	
		GPAT1: NM_001244949	FBpp0084618	mino-PA	minotaur	25.1	LOC120777159	XP_039964647.1	61.8	NC_052499.1 (72000502..72008223)
		GPAT2: NM_207328	FBpp0084619	mino-PC		20.5		XP_039965461.1	63	
DHAPAT, EC 2.3.1.42	1	GNPAT: NM_001316350	FBpp0303401	Gnpat-PC	Glyceronephosphate O-acyltransferase (Gnpat)	23.3	LOC120775111	XP_039961065.1	56.6	NC_052499.1 (15161167..15166004, complement)
ADHAP-S, EC 2.5.1.26	1	AGPS: NM_003659	FBpp0086558	ADPS-PA	Alkylglycerone phosphate synthase	47.0	LOC120771366	XP_039955259.1	65.8	NC_052501.1 (85684093..85690822)
AGPAT/ AAGPAT, EC 2.3.1.51	2	LPLAP1 (APGAT1): NM_032741	FBpp0073521	Agpat1-PA	1-Acylglycerol-3-phosphate O-acyltransferase 1 (AGPAT1)	31.9	LOC120775056	XP_039960970.1	67.9	NC_052502.1 (73419464..73427966, complement)
		LPLAP2 (APGAT2): NM_006412				27.5				
		-	FBpp0309216	Agpat2-PD	1-Acylglycerol-3-phosphate O-acyltransferase 2 (AGPAT2)	-	LOC120781541	XP_039969707.1	62.2	NW_024396366.1 (2307280..2319102, complement)
		LPLAP3 (APGAT3): NM_001037553	FBpp0300976	Agpat3-PE	1-Acylglycerol-3-phosphate O-acyltransferase 3 (AGPAT3)	38.7	LOC120777449	XP_039964700.1	61	NC_052503.1 (49161000..49165656, complement)
		LPLAP4 (APGAT4): NM_020133				36.9				
		LPLAP5 (APGAT5): NM_018361 *	FBpp0075179	Agpat4-PB	1-Acylglycerol-3-phosphate O-acyltransferase 4 (AGPAT4)	21.6			57	
DGAT, EC 2.3.1.20	3	DGAT1: NM_012079.6**	FBpp0080472	mdy-PE	midway	33.6	LOC120767901	XP_039950180.1	75.9	NC_052500.1 (4540873..4570684, complement)
		MOGAT2: NM_025098	FBpp0088002	Dgat2-PA	Diacylglycerol O-acyltransferase 2	38.2	LOC120772092	XP_039956430.1	61.9	NC_052501.1 (28216009..28228763, complement)
			FBpp0308260	CG1941-PC	-	36.1			62.2	
			FBpp0088003	CG1946-PA	-	38.3			61.9	
Δ-1-desaturase, EC 1.14.19.77	1	PEDS1 (TMEM189): NM_199129	FBpp0080868	Kua-PA	Kua	48.4	LOC120768982	XP_039951731.1	69.2	NC_052500.1 (46956250..47012429, complement)

PLA1, EC 3.1.1.32		1	DDHD2: NM_015214	FBpp0308730	PAPLA1-PD	Phosphatidic Acid Phospholipase A1	13.7	LOC120775130	XP_039961092.1	42.9	NC_052502.1 (7159998..7224717)
PLA2, EC 3.1.1.4	Calcium independent	2	PLB1: NM_001170585	FBpp0074636	CG7365-PA	-	10.3	LOC120778470	XP_039966215.1	62.9	NC_052503.1 (63671062..63680534)
	Calcium independent			FBpp0078767	CG11029-PA	-	9.8	LOC120769296	XP_039952159.1	45.7	NC_052500.1 (63865092..63866509, complement)
	Calcium independent		PLA2G6: NM_001004426	FBpp0076092	iPLA2-VIA-PB	calcium-independent phospholipase A2 VIA	31.6	LOC120777471	XP_039964729.1	77.1	NC_052503.1 (10166941..10173262)
	secreted		-	FBpp0304595	CG3009-PD	-	-	LOC120775860	XP_039962164.1	79.3	NC_052502.1 (64778775..64811493)
	secreted		PLA2G1B: NM_000928	FBpp0112217	CG14507-PC	-	12.6	LOC120773488	XP_039958366.1	44.1	NC_052499.1 (89377336..89382673)
	secreted		-	FBpp0088075	CG30503-PA	-	-	LOC120771404	XP_039955313.1	40.3	NC_052501.1 (64238879..64239765, complement)
	secreted		-	FBpp0073553	CG42237-PA	-	-	LOC120775514	XP_039961656.1	60.1	NC_052502.1 (5127858..5145619, complement)
	secreted		PLA2G3: NM_015715	FBpp0292509	GIIIspla2-PB	Phospholipase A2 group III	15.0	LOC120774912	XP_039960718.1	31.4	NC_052502.1 (75394866..75396937)
	secreted		PLA2G12A: NM_030821	FBpp0075264	GXIVsPLA2-PA	GXIVsPLA2	21.7	LOC120779188	XP_039967384.1	71.7	NC_052503.1 (81112015..81113752)
	secreted		-	FBpp0099485	sPLA2-PB	secretory Phospholipase A2	-	LOC120771404	XP_039955313.1	37	NC_052501.1 (64238879..64239765, complement)
PLB, EC 3.1.1.5		1 & 2	OVCA2: NM_080822	FBpp0083317	CG5412-PA	-	27.8	LOC120773391	XP_039958163.1	69.5	NC_052499.1 (56768634..56769852, complement)
			ABHD12: NM_015600	FBpp0085765	CG15111-PA	-	30.6	LOC120772418	XP_039956961.1	61.2	NC_052501.1 (87181102..87187318, complement)
			PNPLA7: NM_152286	FBpp0071077	sws-PA	swiss cheese	40.0	LOC120773795	XP_039958830.1	63.9	NC_052502.1 (7246178..7273079, complement)
LPLAT, EC 2.3.1.-***		1	LPLAP6 (LCLAT1): NM_182551 *	FBpp0075179	Agpat4-PB	1-Acylglycerol-3-phosphate O-acyltransferase 4 (AGPAT4)	23.6	LOC120777449	XP_039964700.1	57	NC_052503.1 (49161000..49165656, complement)
			LPLAP7 (LPGAT1): NM_014873	-	-	-	-	-	-		-
			LPLAP8 (LPCAT1): NM_024830	FBpp0110147	LPCAT-PB	Lysophosphatidylcholine acyltransferase (LPCAT)	33.4	LOC120775046	XP_039960955.1	66.6	NC_052502.1 (36652172..36675735)
			LPLAP9 (LPCAT2): NM_017839				33.2				
			LPLAP10 (LPCAT4): NM_153613				29.7				
			LPLAP11 (MBOAT7): NM_024298	FBpp0078889	frj-PB	farjavit (frj)	31.7	LOC120769360	XP_039952255.1	69.2	NC_052500.1 (70673140..70675562)
			LPLAP12 (LPCAT3): NM_005768	FBpp0074786	nes-PA	nessy (nes)	34.1	LOC120776802	XP_039963730.1	52.4	NC_052503.1 (61891175..61894977, complement)
			LPLAP13 (MBOAT2): NM_138799	FBpp0087520	oys-PA	oysgedart (oys)	29.0	LOC120772337	XP_039956816.1	68.5	NC_052501.1 (122534..142182)
			LPLAP14 (MBOAT1): NM_001080480				27.4				

* Best match in *D. melanogaster* for both LPLAP5 and LPLAP6 from *H. sapiens*

** Human DGAT1 reference deduced from mouse DGAT1 reported in Valentine *et al*. [7]

*** EC numbers for several LPLATs are simply given as EC 2.3.1. because their level four number has not yet been formally assigned (https://www.brenda-enzymes.org/)

Figure S1: Correlations between the abundances and diversities of the various lipid classes in Day 1 and Day 19 males.

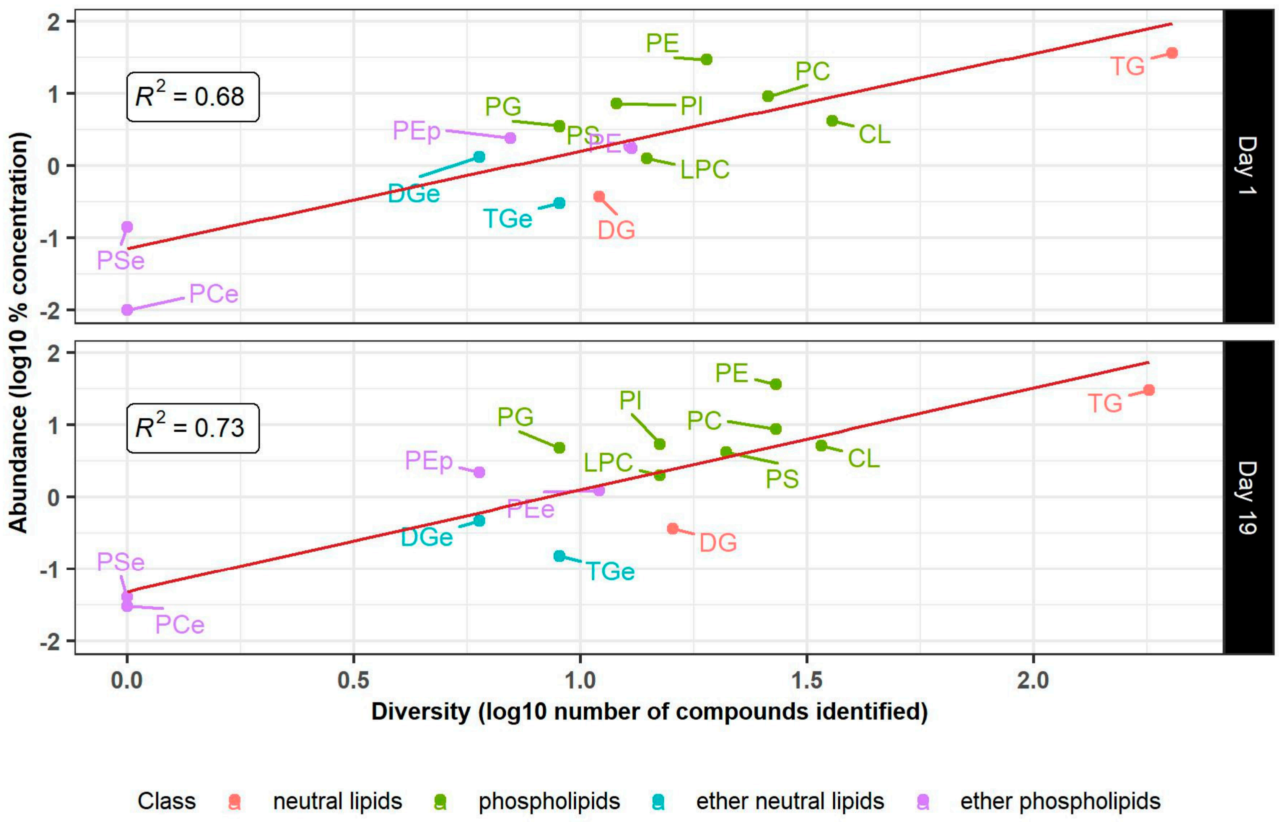

Supplement: Supplementary file 1 [file insects-14-00873-s001.zip › insects-2628162-supplementary materials.pdf]
